# The Complete Female- and Male-Transmitted Mitochondrial Genome of *Meretrix lamarckii*

**DOI:** 10.1371/journal.pone.0153631

**Published:** 2016-04-15

**Authors:** Stefano Bettinazzi, Federico Plazzi, Marco Passamonti

**Affiliations:** Department of Biological, Geological and Environmental Sciences (BiGeA), University of Bologna, Bologna, BO, Italy; Ben-Gurion University of the Negev, ISRAEL

## Abstract

Bivalve mitochondrial genomes show many uncommon features, like additional genes, high rates of gene rearrangement, high A-T content. Moreover, Doubly Uniparental Inheritance (DUI) is a distinctive inheritance mechanism allowing some bivalves to maintain and transmit two separate sex-linked mitochondrial genomes. Many bivalve mitochondrial features, such as gene extensions or additional ORFs, have been proposed to be related to DUI but, up to now, this topic is far from being understood. Several species are known to show this unusual organelle inheritance but, being widespread only among Unionidae and Mytilidae, DUI distribution is unclear. We sequenced and characterized the complete female- (F) and male-transmitted (M) mitochondrial genomes of *Meretrix lamarckii*, which, in fact, is the second species of the family Veneridae where DUI has been demonstrated so far. The two mitochondrial genomes are comparable in length and show roughly the same gene content and order, except for three additional tRNAs found in the M one. The two sex-linked genomes show an average nucleotide divergence of 16%. A 100-aminoacid insertion in *M*. *lamarckii* M-*cox2* gene was found; moreover, additional ORFs have been found in both F and M Long Unassigned Regions of *M*. *lamarckii*. Even if no direct involvement in DUI process has been demonstrated so far, the finding of *cox2* insertions and supernumerary ORFs in *M*. *lamarckii* both strengthens this hypothesis and widens the taxonomical distribution of such unusual features. Finally, the analysis of inter-sex genetic variability shows that DUI species form two separate clusters, namely Unionidae and Mytilidae+Veneridae; this dichotomy is probably due to different DUI regimes acting on separate taxa.

## Introduction

Doubly Uniparental Inheritance (DUI) [1–4] is an interesting alternative to the common Strict Maternal Inheritance (SMI) for cytoplasmic organelles in Eukaryotes.

Species with DUI are characterized by the presence of two different sex-linked mitochondrial lineages, being transmitted independently by the two sexes. One lineage is called F (from Female-transmitted) and it is transmitted by females through eggs, while the other is called M (Male-transmitted) and it is transmitted by males through sperm.

After fertilization, the heteroplasmic zygote contains both F and M mitochondrial lineages. During embryonic development, females become essentially homoplasmic for F, whereas males remain heteroplasmic, the M lineage being localized in germ line and (often in traces) in soma, and the F one in soma only [5]. As a result, in adult DUI bivalves, somatic tissues of both sexes are dominated by the F-mtDNA lineage, while germ-line cells contain the sex-specific mtDNA lineage [6–8]. The two sex-linked mtDNAs are therefore inherited separately, and thus they evolve independently. This results in a high level of sequence divergence between the two genomes, comprised between 10% and 50% (see, f.i., [9, 8, 5, 10]).

Up to now, DUI has only been found in ten bivalve families: Arcticidae, Donacidae, Hyriidae, Margaritiferidae, Mactridae, Mytilidae, Nuculanidae, Solenidae, Unionidae and Veneridae ([11, 6, 12, 7, 9, 8, 13, 10, 14–15]; and reference therein). With the exception of mytilids and unionids, where several DUI species have been discovered, in other families only few DUI species have been found, thus affecting the possibility of comparisons between phylogenetically related species.

Until very recently, among the Veneridae, DUI has been detected in *Venerupis philippinarum* only [16]. Although it was suggested for the species *Cyclina sinensis* [12], this claim was based solely on three GenBank sequences that were recently questioned [10]. In a recent paper, we investigated the presence of DUI in seven species of the subclass Heterodonta and we found DUI only in the venerid clam *Meretrix lamarckii* [10]. *M*. *lamarckii*, also known as the Korean hard clam, is a medium size clam widespread around coasts of Pacific Ocean, including China, Korea, Japan and South-East Asia [17–18]. This economically important mollusk lives in sandy sediments on subtidal flats [19]. A complete mitochondrial genome of *M*. *lamarckii* has already been sequenced from somatic cells by [18]: this is most likely the F-type, as indeed confirmed by phylogenetic analysis [10]. In the present work, we sequenced the complete M and F mitochondrial genomes of *M*. *lamarckii*.

## Materials and Methods

### Samples Collection, DNA Extraction and Dilution

24 individuals of *Meretrix lamarckii* were commercially purchased at the Tsukiji Wholesale Fish Market (Tokyo, Japan) in June 2012. All specimens were screened alive by microscopic inspection of gonadal extract to confirm sexual maturity and to determine the sex. A standard phenol:chloroform protocol [20] was used to extract total nucleic acid from gametes; samples were re-suspended in TE 1× and are conserved in the Mozoo Lab at the Department of Biological, Geological and Environmental Sciences (Bologna, Italy).

Total nucleic acid was quantified using a Nanodrop spectrophotometer and further diluted to reach optimal Long-PCR concentration (125 ng/μL). Two individuals, whose specimen numbers are BES:TKJ:004 (female) and BES:TKJ:009 (male), were selected because of yield results for further sequencing.

### PCR Amplifications, Electrophoresis and Sequencing

The two complete genomes were amplified in three large overlapping fragments using Long-PCR technique paired with primer-walking on a Gene Amp® PCR System 2720 (Applied Biosystem). To perform Long PCR amplification from 2,000 bp up to 10,000 bp we used the Herculase® II Fusion Enzyme kit (Stratagene). Reaction volume of 50 μL was composed as follows: 2 μL DNA template (125 ng/μL), 0.5 μL Herculase II Fusion DNA Polymerase, 10 μL 5× Herculase II reaction Buffer (containing Mg^2+^ 10 mM), 0.5 μL dNTPs mix (25 mM for each dNTP), 1.25 μL each Primer (10 μM) and 34.5 μL of sterilized distilled water.

Reaction conditions (S1 Table) were first set up according to manufacturer’s instruction and further modified whenever necessary. After the initial denaturation step (92°C for 2’), 30 cycles were used as follows: denaturation at 92°C for 20”, annealing at 48–56°C for 20–30” and extension at 68°C for 10’. The final extension step was carried out at 68°C for 8’. Primers were designed using the Primer3 online tool [21].

For amplification of fragments < 2,000 bp we used a standard PCR approach using the GoTaq® Flexi DNA Polymerase kit (Promega). The reaction volume was 30 μL composed of 10.85 μL of sterilized distilled water, 6 μL of 5× Green GoTaq® Flexi Buffer, 2.4 μL of dNTPs (2.5 mM for each dNTP), 3.6 μL of MgCl_2_ (25 mM), 1.5 μL of each primer (10 μM), 0.15 μL of *Taq* Polymerase (5u/μL) and 4 μL of appropriately diluted DNA template. Typically, the cycle (S1 Table) was composed by an initial denaturation step (95°C for 2’), then 35 cycles as follows: denaturation at 95°C for 1’, annealing at 48–56°C for 1’ and extension at 72°C for 1–2’ depending on amplicons length. The final extension was carried out at 72°C for 5’.

PCR results were visualized by electrophoresis onto a 1% agarose gel stained with ethidium bromide and then purified through a standard isopropanol protocol, Wizard® SV Gel and PCR Clean-Up System (Promega) and, in some cases, with an empirically modified PEG precipitation protocol [22]. Successfully purified products were sequenced with the Sanger method thanks to Macrogen Europe facility (Amsterdam, The Netherland).

### Data analysis and genome annotation

MEGA 6.06 [23] was used to examine and edit electropherograms and further merge the entire mitochondrial genomes according to overlapping sequences. This software was also used to compute codon usage and nucleotide percentages.

Nucleotide trends and A-T skew at four-fold degenerate sites were used to identify the Control Region (CR) and the Origin of Replication (OR) of either strand. A dedicated R [24] script was written to (i) compute these statistics over a sliding window, (ii) plot results and (iii) test for significance of the linear correlation. Any size and step for the sliding window can be specified: for the present work, we used a 700-bp sliding window with a step of 300 bp. Autocorrelograms for each nucleotide are also produced in order to evaluate the amount of autocorrelation between sliding windows and therefore the validity of the linear model approach. The user is allowed to analyze several genomes at the same time and to set any gene as starting point for the four-fold degenerate sites analysis; the script is available as S1 Script along with test files and a detailed tutorial. A GitHub repository was created, which can be found at the URL https://github.com/mozoo/4F.git.

Protein Coding Genes (PCGs) were predicted using both MITOS [25] and NCBI's ORF Finder online tool [26], using invertebrate mitochondrial genetic code and alternative start codons. Potential PCGs were identified through homologous sequence similarity using BLAST [27–28]. F- and M-*cox2* sequences were aligned using the software MUSCLE [29] and the alignment was graphically edited thanks to the TeXshade package [30]. Putative tRNA genes were detected using MITOS, tRNAScan-SE [31–32] and ARWEN v1.2 [33] online softwares, using default settings. rRNA sequences have been identified by comparisons with other rRNAs present in GenBank using BLAST. In both F and M genomes, the rRNAs sequences were then annotated assuming that the first base comes immediately after the last base of the previous gene and that the last base comes immediately before the first base of the following gene. Finally, the two whole-genome maps were created using GenomeVx online tool [34], conventionally setting *cox1* as the starting point of the mtDNA. All putative secondary structures of rRNAs and non-coding regions were predicted using Mfold server [35] and then graphically edited through VARNA 3.7 [36].

Repeats were identified through the online software Tandem Repeat Finder [37]. Phobius [38], InterProScan [39], TMPred [40] and HMMTOP [41] online softwares were all used to predict additional Trans Membrane Helices (TMHs). Introns were searched using @TOME2 [42]. The analysis of additional putative ORFs was done using the software Glimmer3 [43]. The EMBOSS [44] package was used to extract all the possible ORFs from all available bivalve complete mitochondrial genomes (GenBank consulted in August, 2014). An alignment was computed for F_ORF141 and M_ORF138 using HHBlits [45] and the last UniProt release. The computed hidden Markov model was used as a query against the database of all bivalve mitochondrial ORFs, which was built using HHBlits and the Pdb70 database.

### Phylogeny and evolutionary comparisons

A phylogenetic analysis was carried out using other complete mitochondrial genomes from the family Veneridae that were available at August, 2014. Three heterodonts, *Coelomactra antiquata* (Mactridae), *Hiatella arctica* (Hiatellidae), and *Acanthocardia tuberculata* (Cardiidae) were selected as outgroups; the complete dataset is available as S2 Table. The 13 PCGs and the 2 rRNAs were extracted and aligned with PSI-BLAST [46], MUSCLE, ProbconsRNA [47], RNAplfold [48], and MAFFT [49] through the T-Coffee algorithm [50–51], using the pipeline PSI-Coffee > Expresso > **accurate** for PCGs and the MR-Coffee mode for rRNAs. Alignments were masked using BMGE 1.1 [52]; the best partitioning scheme was selected using PartitionFinder 1.1.0 [53] under the Bayesian Information Criterion and a greedy approach. The final Maximum Likelihood (ML) tree search was carried out with RAxML 8.2.0 [54] performing 1,000 bootstrap replicates. The consensus tree was computed with PhyUtility 2.2 [55] and graphically edited with Dendroscope 3 [56].

Finally, we compared sex-linked divergence in different DUI systems; all the DUI species whose complete mitochondrial genomes were available in January, 2015 were selected for this analysis. M and F coding sequences of *M*. *lamarckii* and other DUI species were aligned gene by gene using the T-Coffee algorithm: as above, the **accurate** method was used for PCGs, while MR-Coffee was chosen for rRNAs and tRNAs. To account for multiple substitutions, nucleotide Jin-Nei [57] and aminoacid Kimura [58] corrected distances were then computed using the EMBOSS suite along with uncorrected *p*-distances. Principal Component Analysis was carried out through R on concatenated Jin-Nei and Kimura distances using the packages FactoMineR [59] for computations and ggplot2 [60] for graphics. We also computed average distances within Unionidae, within Amarsipobranchia *sensu* [61] (i.e., Pteriomorphia + Heterodonta; in this case, Mytilidae + Veneridae), and within the complete dataset. Nucleotide single-gene average values were ranked and rankings within Unionidae and Amarsipobranchia were compared through R using the Spearman ρ and the Kendall's τ.

## Results

### Overall genomic features

**Fig 1:**
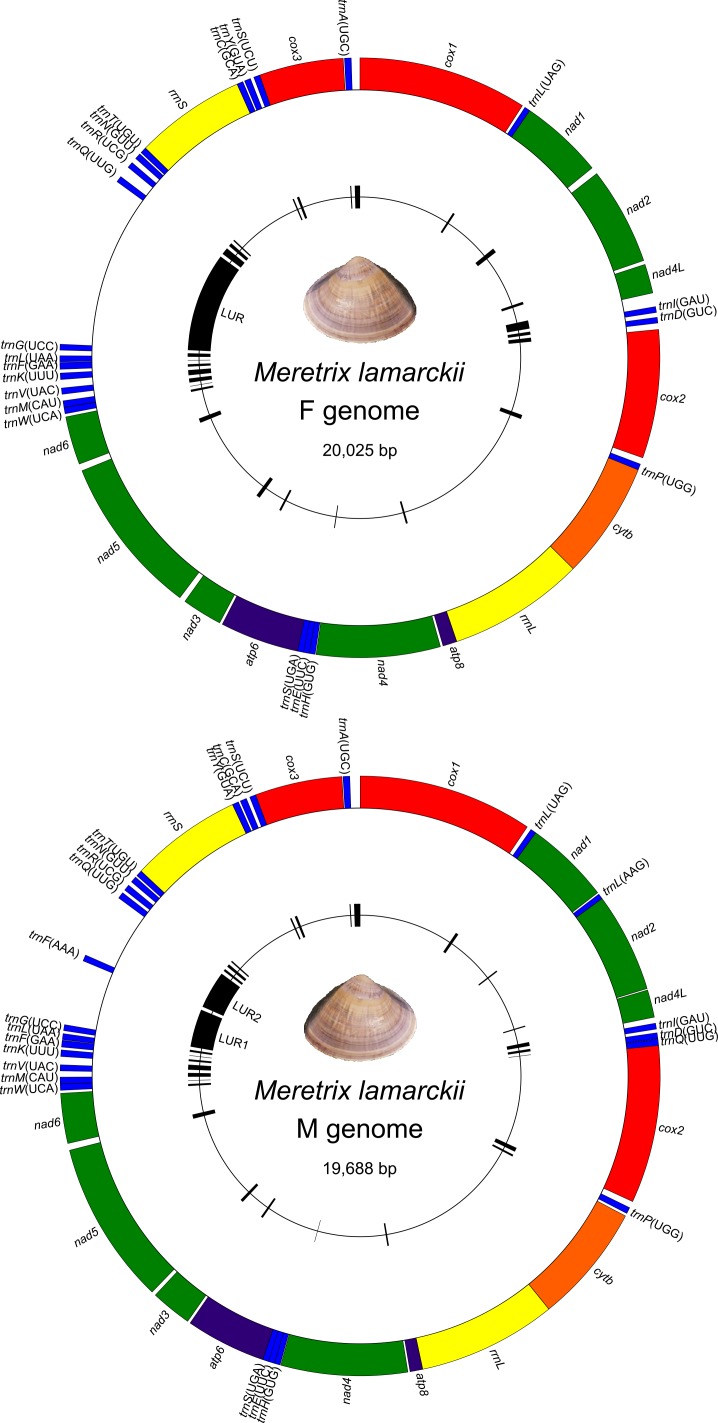
*Meretrix lamarckii* sex-linked mitochondrial genomes. Genomic map of F- (above) and M- (below) mtDNA of *Meretrix lamarckii* starting from *cox1*. Genes are all on "+" strand; genome lengths are shown in the middle of each map. Unassigned Regions (URs) are reported in black in the internal circle.

The *Meretrix lamarckii* complete F and M mitochondrial genomes are 20,025 bp and 19,688 bp long, respectively (Fig 1). Sequences are available in GenBank under the accession numbers KP244451 and KP244452, respectively. All genes are located on the same “+” strand and the two lineages share the same gene order. The only exceptions are three tRNAs, which were found only in M-mtDNA: *trnL*(AAG), an additional copy of *trnQ*(UUG), and *trnF*(AAA). Genome annotations are shown in Table 1.

**Table 1:** Annotation of F and M mitochondrial genomes of *Meretrix lamarckii*.

Name		Type^a^	Start^b^	End	Lenght (bp)	UNs^c^	Anticodon	Start Codon	Stop Codon
*cox1*	F	PCG	1	1,824	1,824	32		GTG	TAG
	M	PCG	1	1,851	1,851	46		GTG	TAA
*trnL*(NAG)	F	tRNA	1,857	1,918	62	0	TAG		
	M	tRNA	1,898	1,959	62	0	TAG		
*nad1*	F	PCG	1,919	2,821	903	77		ATC	TAA
	M	PCG	1,960	2,862	903	19		ATT	TAA
*trnL*(NAG)	M	tRNA	2,882	2,939	58	0	AAG		
*nad2*	F	PCG	2,899	3,954	1,056	42		GTG	TAA
	M	PCG	2,940	3,995	1,056	15		ATG	TAA
*nad4L*	F	PCG	3,997	4,311	315	148		GTG	TAG
	M	PCG	4,011	4,304	294	63		ATT	TAG
*trnI*	F	tRNA	4,460	4,523	64	51	GAT		
	M	tRNA	4,368	4,430	63	38	GAT		
*trnD*	F	tRNA	4,575	4,636	62	68	GTC		
	M	tRNA	4,469	4,533	65	4	GTC		
*trnQ*	M	tRNA	4,538	4,604	67	-2	TTG		
*cox2*	F	PCG	4,705	6,090	1,386	71		ATG	TAG
	M	PCG	4,603	6,276	1,674	70		ATG	TAA
*trnP*	F	tRNA	6,162	6,227	66	-1	TGG		
	M	tRNA	6,347	6,414	68	29	TGG		
*cytb*	F	PCG	6,227	7,492	1,266	0		ATG	TAG
	M	PCG	6,444	7,712	1,269	0		TTG	TAG
*rrnL*	F	rRNA	7,493	8,964	1,472	0			
	M	rRNA	7,713	9,185	1,473	0			
*atp8*	F	PCG	8,965	9,117	153	28		ATA	TAA
	M	PCG	9,186	9,320	135	22		ATG	TAG
*nad4*	F	PCG	9,146	10,480	1,335	9		ATA	TAA
	M	PCG	9,343	10,683	1,341	7		GTG	TAA
*trnH*	F	tRNA	10,490	10,551	62	0	GTG		
	M	tRNA	10,691	10,752	62	0	GTG		
*trnE*	F	tRNA	10,552	10,617	66	-3	TTC		
	M	tRNA	10,753	10,818	66	-3	TTC		
*trnS*(NGA)	F	tRNA	10,615	10,679	65	0	TGA		
	M	tRNA	10,816	10,880	65	0	TGA		
*atp6*	F	PCG	10,680	11,531	852	31		ATG	TAG
	M	PCG	10,881	11,732	852	31		GTG	TAG
*nad3*	F	PCG	11,563	11,997	435	65		ATG	TAG
	M	PCG	11,764	12,198	435	46		GTG	TAG
*nad5*	F	PCG	12,063	13,787	1,725	76		GTG	TAG
	M	PCG	12,245	13,981	1,737	72		GTG	TAA
*nad6*	F	PCG	13,864	14,385	522	34		ATA	TAA
	M	PCG	14,054	14,584	531	31		ATA	TAA
*trnW*	F	tRNA	14,420	14,487	68	2	TCA		
	M	tRNA	14,616	14,682	67	2	TCA		
*trnM*	F	tRNA	14,490	14,559	70	70	CAT		
	M	tRNA	14,685	14,754	70	64	CAT		
*trnV*	F	tRNA	14,630	14,696	67	90	TAC		
	M	tRNA	14,819	14,883	65	86	TAC		
*trnK*	F	tRNA	14,787	14,856	70	39	TTT		
	M	tRNA	14,970	15,038	69	30	TTT		
*trnF*	F	tRNA	14,896	14,969	74	7	GAA		
	M	tRNA	15,069	15,140	72	6	GAA		
*trnL*(NAA)	F	tRNA	14,977	15,040	64	59	TAA		
	M	tRNA	15,147	15,210	64	31	TAA		
*trnG*	F	tRNA	15,100	15,163	64	1,855	TCC		
	M	tRNA	15,242	15,305	64	694	TCC		
*trnF*	M	tRNA	16,000	16,066	67	690	AAA		
*trnQ*	F	tRNA	17,019	17,086	68	125	TTG		
	M	tRNA	16,757	16,825	69	35	TTG		
*trnR*	F	tRNA	17,212	17,277	66	57	TCG		
	M	tRNA	16,861	16,926	66	45	TCG		
*trnN*	F	tRNA	17,335	17,400	66	19	GTT		
	M	tRNA	16,972	17,037	66	20	GTT		
*trnT*	F	tRNA	17,420	17,487	68	0	TGT		
	M	tRNA	17,058	17,122	65	0	TGT		
*rrnS*	F	rRNA	17,488	18,680	1,193	0			
	M	rRNA	17,123	18,310	1,188	0			
*trnC*	F	tRNA	18,681	18,747	67	19	GCA		
	M	tRNA	18,311	18,378	68	21	GCA		
*trnY*	F	tRNA	18,767	18,834	68	38	GTA		
	M	tRNA	18,400	18,469	70	37	GTA		
*trnS*(NCT)	F	tRNA	18,873	18,939	67	0	TCT		
	M	tRNA	18,507	18,573	67	0	TCT		
*cox3*	F	PCG	18,940	19,848	909	12		ATG	TAA
	M	PCG	18,574	19,497	924	11		ATG	TAA
*trnA*	F	tRNA	19,861	19,933	73	92	TGC		
	M	tRNA	19,509	19,579	71	109	TGC		

^a^ PCG, Protein Coding Gene.

^b^ All genes are located on the same strand.

^c^ Unassigned nucleotides after the gene (negative values for overlapping nucleotides).

Nucleotide composition is reported in Table 2. *M*. *lamarckii* A-T content reaches 66.01% in F-mtDNA and 67.17% in M-mtDNA. This value increases considering only the 3^rd^ base nucleotide composition of PCG codons (73.31% for F-mtDNA and 75.08% for M-mtDNA).

**Table 2:** Nucleotide composition (%) of *Meretrix lamarckii* F and M genomes.

Name	Sex	Length	T	C	A	G	A-T	T3^a^	C3^a^	A3^a^	G3^a^	A-T3^a^
*cox1*	F	1,824	43.10	12.10	22.10	22.70	65.20	56.00	4.90	18.90	19.70	74.90
	M	1,851	44.30	11.30	22.70	21.60	67.00	58.35	4.21	20.10	17.34	78.44
*nad1*	F	903	45.96	11.52	19.27	23.26	65.23	55.81	4.98	18.94	20.27	74.75
	M	903	47.07	10.63	18.83	23.48	65.89	57.81	3.32	17.61	21.26	75.42
*nad2*	F	1,056	44.41	7.39	23.20	25.00	67.61	46.02	3.98	30.68	19.32	76.70
	M	1,056	44.89	6.63	21.69	26.80	66.57	48.01	2.27	23.58	26.14	71.59
*nad4L*	F	315	50.16	8.25	19.37	22.22	69.52	52.38	3.81	17.14	26.67	69.52
	M	294	51.70	9.52	18.71	20.07	70.41	53.06	7.14	16.33	23.47	69.39
*cox2*	F	1,386	38.89	9.52	25.69	25.90	64.57	56.28	5.63	18.83	19.26	75.11
	M	1,674	37.48	9.15	28.21	25.16	65.69	53.23	4.84	22.40	19.53	75.63
*cytb*	F	1,266	43.68	13.03	21.88	21.41	65.56	54.27	7.11	21.33	17.30	75.59
	M	1,269	44.52	13.00	20.72	21.75	65.25	54.85	6.62	20.80	17.73	75.65
*atp8*	F	153	45.10	12.42	22.22	20.26	67.32	43.14	3.92	27.45	25.49	70.59
	M	135	50.37	9.63	19.26	20.74	69.63	51.11	0.00	17.78	31.11	68.89
*nad4*	F	1,335	45.39	10.49	19.78	24.34	65.17	51.24	5.62	21.80	21.35	73.03
	M	1,341	44.74	10.07	22.22	22.97	66.96	49.66	5.37	25.28	19.69	74.94
*atp6*	F	852	43.78	10.92	23.12	22.18	66.90	45.07	6.34	25.00	23.59	70.07
	M	852	44.13	10.21	23.47	22.18	67.61	46.83	3.87	29.93	19.37	76.76
*nad3*	F	435	43.45	8.97	25.75	21.84	69.20	45.52	4.83	29.66	20.00	75.17
	M	435	42.99	7.13	25.52	24.37	68.51	44.14	2.76	24.83	28.28	68.97
*nad5*	F	1,725	46.09	10.96	17.74	25.22	63.83	49.39	8.70	16.00	25.91	65.39
	M	1,737	47.67	8.52	19.80	24.01	67.47	54.06	3.63	20.03	22.28	74.09
*nad6*	F	522	47.51	10.34	18.20	23.95	65.71	56.32	6.32	16.67	20.69	72.99
	M	531	48.96	8.10	18.27	24.67	67.23	58.76	4.52	12.99	23.73	71.75
*cox3*	F	909	44.66	11.88	19.47	23.98	64.14	62.05	4.62	15.51	17.82	77.56
	M	924	46.10	9.63	19.59	24.68	65.69	62.34	1.95	15.91	19.81	78.25
*rrnS*	F	1,193	36.46	10.73	28.50	24.31	64.96					
	M	1,188	37.29	9.76	28.96	23.99	66.25					
*rrnL*	F	1,472	39.27	9.71	29.82	21.20	69.09					
	M	1,473	39.24	9.91	30.69	20.16	69.93					
All PCGs^b^	F	12,681	44.21	10.79	21.30	23.70	65.51	52.78	5.82	20.53	20.87	73.31
	M	13,002	44.67	9.75	22.05	23.52	66.73	53.88	4.15	21.20	20.77	75.08
All rRNAs	F	2,665	38.01	10.17	29.23	22.59	67.24					
	M	2,661	38.37	9.85	29.91	21.87	68.28					
All tRNAs	F	1,464	37.70	10.93	29.71	21.65	67.42					
	M	1,653	39.21	9.76	29.45	21.58	68.67					
All coding DNA^c^	F	16,810	42.66	10.70	23.29	23.35	65.95					
	M	17,316	43.18	9.77	23.97	23.08	67.15					
All URs^d^	F	3,216	40.14	7.37	26.18	26.31	66.32					
	M	2,374	41.29	6.59	26.20	25.91	67.50					
Complete genome	F	20,025	42.26	10.17	23.75	23.83	66.01					
	M	19,688	42.95	9.39	24.23	23.43	67.19					

^a^ Computed only on third codon positions.

^b^ PCGs, Protein Coding Genes.

^c^ PCGs + rRNAs + tRNAs

^d^ URs, Untranslated Regions.

Both F-type and M-type mtDNAs contain a large numbers of Unassigned Regions (URs; 27 in F-mtDNA and 29 in M-mtDNA), which are detailed in S3 Table.

### Protein Coding Genes (PCGs)

We found all 13 canonical protein coding genes, including the *atp8* gene, reported as missing in several bivalve species [7, 62–63]. ATG start codon is used in *cox2*, *cytb*, *atp6*, *nad3*, and *cox3* of the F genome and in *nad2*, *cox2*, *atp8* and *cox3* of the M one. Like most invertebrate mitochondrial genomes, the two *M*. *lamarckii* mtDNAs show alternative start codons: GTG, ATC, ATA, ATT and TTG (see Table 1). Observed stop codons are TAG and TAA, as expected. Overall, TAA is the most common stop codon, while TAG is used in F- *cox1*, *nad4L*, *cox2*, *cytb*, *atp6*, *nad3*, and *nad5* and in M- *nad4L*, *cytb*, *atp8*, *atp6*, and *nad3*. Truncated TA-/T—stop codons ([8, 63]; and reference therein) were not found in *M*. *lamarckii* mtDNAs.

*M*. *lamarckii* F and M protein coding genes (PCGs) contain 4,227 codons and 4,333 codons, respectively (Table 3). In both F and M mtDNAs, the most used codons is UUU (410 and 434 hits, respectively). Less used codons are CGC and ACC (both 7 hits) in F-mtDNA and UGC (2 hits) in M-mtDNA. The most common aminoacid in both F- and M-mtDNA is leucine, while the rarest is glutamic acid.

**Table 3:** *Meretrix lamarckii* codon count (#) and usage (%).

aa	codon^a^		#	%	aa	codon^a^		#	%	aa	codon^a^		#	%	aa	codon^a^		#	%
Phe(F)	UUU^b^	F	410	9.70	Ser(S)	UCU	F	161	3.81	Ala(A)	GCU	F	134	3.17	Asp(D)	GAU	F	93	2.20
		M	434	10.02			M	158	3.65			M	122	2.82			M	114	2.63
	UUC	F	42	0.99		UCC	F	9	0.21		GCC	F	9	0.21		GAC	F	19	0.45
		M	17	0.39			M	11	0.25			M	7	0.16			M	15	0.35
Leu(L)	UUA	F	230	5.44		UCA	F	16	0.38		GCA	F	26	0.62	Glu(E)	GAA	F	46	1.09
		M	244	5.63			M	21	0.48			M	26	0.60			M	67	1.55
	UUG	F	190	4.49		UCG	F	15	0.35		GCG	F	28	0.66		GAG	F	78	1.85
		M	193	4.45			M	18	0.42			M	21	0.48			M	70	1.62
	CUU^b^	F	82	1.94		AGU	F	88	2.08	Tyr(Y)	UAU	F	152	3.60	Cys(C)	UGU	F	84	1.99
		M	91	2.10			M	103	2.38			M	171	3.95			M	99	2.28
	CUC	F	9	0.21		AGC	F	13	0.31		UAC	F	25	0.59		UGC	F	10	0.24
		M	3	0.07			M	11	0.25			M	18	0.42			M	2	0.05
	CUA	F	25	0.59		AGA	F	66	1.56	STOP(*)	UAA	F	6	0.14	Trp(W)	UGA	F	58	1.37
		M	11	0.25			M	52	1.20			M	8	0.18			M	52	1.20
	CUG	F	17	0.40		AGG	F	51	1.21		UAG	F	7	0.17		UGG	F	66	1.56
		M	17	0.39			M	67	1.55			M	5	0.12			M	72	1.66
Ile (I)	AUU	F	198	4.68	Pro(P)	CCU	F	89	2.11	His(H)	CAU	F	51	1.21	Arg(R)	CGU	F	40	0.95
		M	235	5.42			M	95	2.19			M	51	1.18			M	39	0.90
	AUC	F	14	0.33		CCC	F	11	0.26		CAC	F	20	0.47		CGC	F	7	0.17
		M	9	0.21			M	8	0.18			M	16	0.37			M	4	0.09
Met(M)	AUA	F	84	1.99		CCA	F	16	0.38	Gln(Q)	CAA^c^	F	27	0.64		CGA	F	23	0.54
		M	114	2.63			M	18	0.42			M	25	0.58			M	15	0.35
	AUG	F	117	2.77		CCG	F	8	0.19		CAG	F	30	0.71		CGG	F	13	0.31
		M	90	2.08			M	11	0.25			M	29	0.67			M	14	0.32
Val(V)	GUU	F	275	6.51	Thr(T)	ACU	F	87	2.06	Asn(N)	AAU	F	102	2.41	Gly(G)	GGU	F	185	4.38
		M	266	6.14			M	90	2.08			M	102	2.35			M	165	3.81
	GUC	F	14	0.33		ACC	F	7	0.17		AAC	F	24	0.57		GGC	F	13	0.31
		M	11	0.25			M	6	0.14			M	21	0.48			M	21	0.48
	GUA	F	71	1.68		ACA	F	18	0.43	Lys(K)	AAA	F	85	2.01		GGA	F	71	1.68
		M	95	2.19			M	13	0.30			M	100	2.31			M	57	1.32
	GUG	F	116	2.74		ACG	F	20	0.47		AAG	F	47	1.11		GGG	F	79	1.87
		M	120	2.77			M	16	0.37			M	53	1.22			M	104	2.40

^a^ Codons that match a corresponding mtDNA-encoded tRNA are underlined.

^b^ The corresponding tRNA is present only in the M genome.

^c^ Two *trnQ*(UUG) tRNAs were detected in the M genome.

Post-transcriptional cleavage sites could be indicated by the presence of a tRNA between two PCGs [64]. In absence of a tRNA, the cleavage role can be played by intergenic non-coding sequences that form a stem-loop secondary structure ([8]; and reference therein). According to the previous statement, for each *M*. *lamarckii* unassigned region located between a pair of PCGs, the predicted hairpin was determined and reported in S1 Fig.

**Fig 2:**
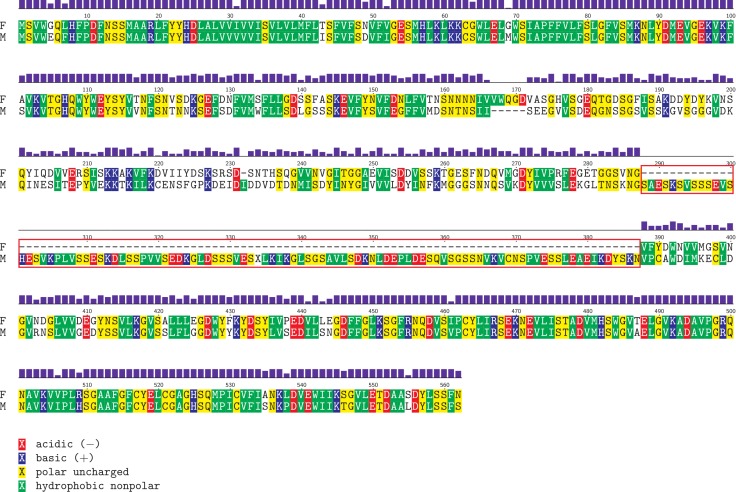
*Meretrix lamarckii cox2* gene alignment. Aminoacid alignment between female-type (F) and male-type (M) *Meretrix lamarckii* sequences of the *cox2* gene. Identical aminoacids are shaded following their hydropathy (see the legend below the figure for the meanings of the different colors); purple bars show aminoacid similarity. The 100-aminoacid insertion found in M-mtDNA is boxed in red. Sites are numbered above the sequences, conventionally starting at 1.

Finally, M-*cox2* gene is significantly different from the F one. More specifically, it includes a 100-aminoacid long region in the middle of the gene, which is not present in F-*cox2* (Fig 2).

### rRNAs and tRNAs

Standard rRNAs were found in both genomes: *rrnS* is located between *trnT* and *trnC*, while *rrnL* between *cytb* and *atp8*. The F and the M rRNAs predicted secondary structures are reported in S2 Fig.

F-mtDNA shows all 22 canonical tRNAs, with two serine-encoding tRNAs and two leucine-encoding tRNAs. They may differ from each other in terms of anticodon. Like many other metazoan taxa (see, f.i., [65, 8, 63]), both F- and M-*trnS*(UCU) present a shortened DHU arm.

In addition, the M genome presents three sex-specific tRNAs, totaling 25 tRNAs: supernumerary *trnL*(AAG) (between *nad1* and *nad2*), *trnQ*(UUG) (between *trnD* and *cox2*), and *trnF*(AAA) (within M Long Unassigned Region). All secondary structures of tRNAs are reported in S3 and S4 Figs.

To better understand their origin, M supernumerary tRNAs were compared with the URs mapped in the same position of F-mtDNA. In all cases, a very similar sequence was found, albeit the canonical cloverleaf structure is essentially unrecoverable (S5 Fig).

### Long Unassigned Region (LUR)

A Long Unassigned Region (LUR) is located between *trnG* and *trnQ*. F-LUR measures 1,855 bp, whereas M-LUR is apparently divided in two regions (LUR1 and LUR2 of 694 bp and 690 bp, respectively) by the putative supernumerary *trnF*(AAA) (see above).

A complex secondary structure was found in both M and F mtDNAs in the middle LUR sequence. This highly folded structure is comprised between bases 15,698 and 15,952 in F-LUR and between bases 15,838 and 16,512 in M-LUR. In the F-LUR two tandem-repeated motifs were also found, both with two tandem copies. The first motif is 15 bp long (positions 15,722–15,736 and 15,737–15,751) and the second one is 109 bp long (positions 16,767–16,875 and 16,880–16,988, at the end of F-LUR region) (S6 Fig). In M-mtDNA only the 15 bp-long motif was found (positions 15,845–15,858 and 15,860–15,873). A BLAST search of the Termination-Associated Sequence (TAS; [66–67]) element found a significant hit (S7 Fig) in the F-LUR (positions 16,340–16,354), but not in the M-LUR.

**Fig 3:**
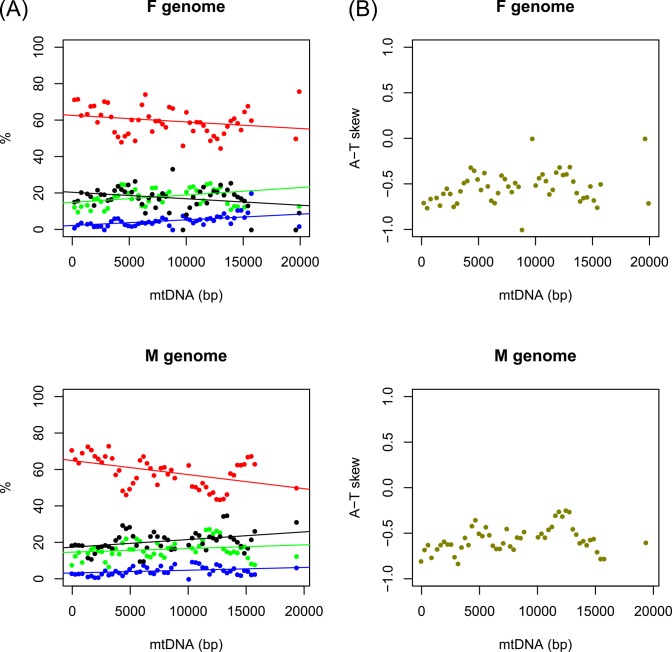
Origins of Replication. A, nucleotide composition at four-fold degenerate sites, using a sliding window of 700 bp with a step of 300 bp. The starting point is the first PCG after the LUR, i.e. *cox3*. Equations are as follows, for F/M, respectively. A (green): *y = 0*.*0004x+14*.*84*; *R*^*2*^ *= 0*.*0681; p = 0*.*0616* / *y = 0*.*0002x+14*.*62*; *R*^*2*^ *= 0*.*0409; p = 0*.*1546*. C (blue): *y = 0*.*0003x+2*.*15*; *R*^*2*^ *= 0*.*2263; p = 0*.0004*** / *y = 0*.*0002x+3*.*17*; *R*^*2*^ *= 0*.*1087; p = 0*.0181*. G (black): *y = −0*.*0004x+20*.*39*; *R*^*2*^ *= 0*.*0796; p = 0*.0428* / *y = 0*.*0004x+17*.*33*; *R*^*2*^ *= 0*.*1448; p = 0*.0059**. T (red): *y = −0*.*0004x+62*.*61*; *R*^*2*^ *= 0*.*0595; p = 0*.*0815* / *y = −0*.*0008x+64*.*88*; *R*^*2*^ *= 0*.*2123; p = 0*.0007***. B, A-T skew at four-fold degenerate sites, using a sliding window as for (A); the starting point is again *cox3*.

The first PCG downstream of the LUR is *cox3*; therefore, we set *cox3* as the starting point of the sliding window computing nucleotide composition at four-fold degenerate sites. We found 1,911 degenerate sites in the F *M*. *lamarckii* genome (15.01% of PCG sites) and 1,907 in the M one (14.67%). In both cases, four-fold degenerate sites are highly T-rich (59.65% for F and 59.20% for M) and definitely weak, but significant trends were uncovered (Fig 3A): while a significant A trend was never found, we detected a significant increase in C in both sexes, even if with very low R^2^ values. A negative trend for G was found in F-mtDNA, while a positive one for G and T was found in M-mtDNA, again with very low R^2^ values. The autocorrelograms show a significant value of the autocorrelation function (acf) only at lag-1 for F genomes, or at lag-1 and lag-2 (or lag-5) for three M nucleotides (S8 Fig). Given the T-richness of four-fold degenerate sites, the A-T skew is always negative or equal to 0, but two peaks were found, corresponding to the LUR and to the *atp6*/*nad3* boundary (Fig 3B).

The structure of the F-LUR is comparable to the LUR of the published genome of *M*. *lamarckii* (GenBank Accession Number NC_016174), with some differences. The LUR of the available *M*. *lamarckii* mtDNA is found at positions 14,982–18,044; again, a highly folded region can be inferred (15,470–16,433). At the 5' side of the highly folded region there is a sequence very similar to that of the F-ORF (15,242–15,392); this sequence would be a putative ORF located on the reverse strand, were it not for a stop codon (TAA) right after the start one and for an insertion of a G, which triggers a frameshift mutation leading to the loss of the stop codon (S9 Fig). Conversely, at the 3' side of the highly folded region a 100-bp repeated motif was found; the repeat unit shows some similarities with the 109-bp motif of our F genome (S10 Fig). However, in the GenBank *M*. *lamarckii* mtDNA it is repeated 13 times; this higher number of repeats accounts for the great difference in length between the two LURs (1,855 against 3,063 bp).

### Supernumerary Open Reading Frames (ORFs)

Several additional putative Open Reading Frames (ORFs) were found within the LUR of both F and M *M*. *lamarckii* mtDNAs. Among all these sequences, we found only one ORF in each genome (F_ORF141 and M_ORF138) that does not overlap with the highly folded structure revealed in the LUR (see above). To better understand whether these putative ORFs are expressed or not, the prediction software Glimmer3 was used. At first, the software was trained with *M*. *lamarckii* standard gene data. All mitochondrial PCGs were given a score comprised between 8.71 and 16.34 (for F) and between 10.47 and 18.08 (for M). According to these values, the two potential supernumerary ORFs should not be considered as expressed, because they showed extremely low scores (i.e., 2.34 for F_ORF141 and 3.36 for M_ORF138; see S4 Table).

The presence of F_ORF141 was also searched for in all available bivalve complete mitochondrial genomes using HHBlits. In all the other available *Meretrix* species, a homolog was found in the reverse strand, within the LUR. All homologous ORFs have a probability over 90%, while *E*-value and *p*-value were always lower than 0.05; this holds also for the F_ORF141/M_ORF138 comparison (S5 Table).

### Phylogenetic analysis

**Fig 4:**
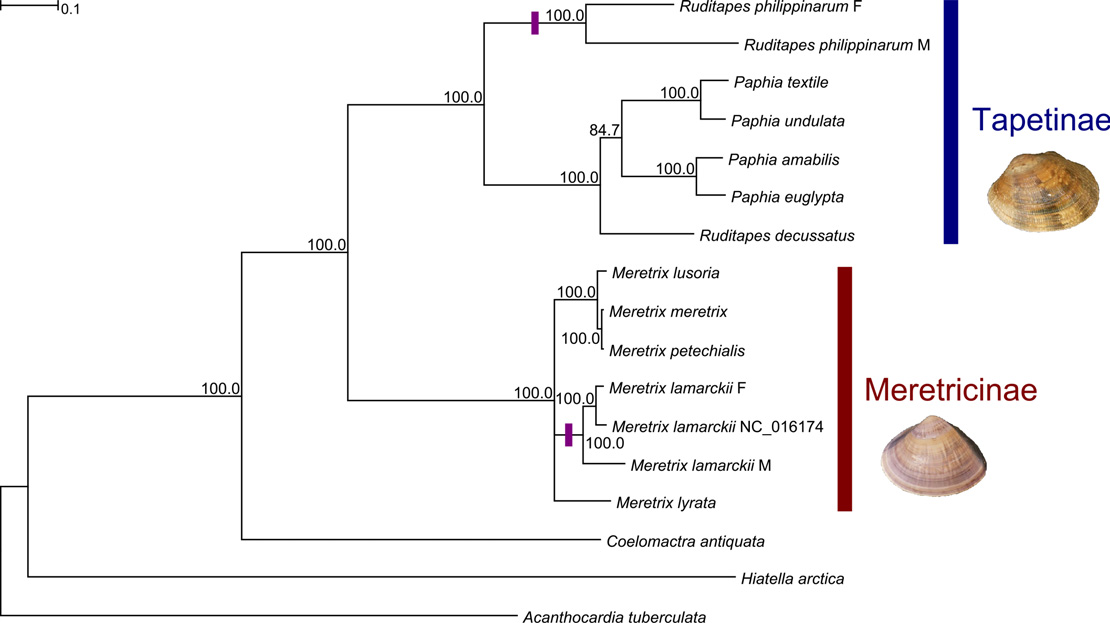
Phylogenetic analysis. Maximum Likelihood phylogenetic analysis of the family Veneridae using complete mitochondrial genomes and *Acanthocardia tuberculata* (Cardiidae), *Hiatella arctica* (Hiatellidae), and *Coelomactra antiquata* (Mactridae) as outgroups. Shown is the consensus of ML trees obtained from 1,000 bootstrap replicates; number at the nodes are bootstrap proportions. Purple bars mark known DUI species.

The complete dataset was composed by 5,035 aminoacids (PCGs) and 4,554 nucleotides (rRNAs). 3,841 aminoacids and 1,232 nucleotides were left for phylogenetic analysis (69.14% and 27.05%, respectively) after masking with BMGE; the most affected PCG was *cox2* (only 29.82% aminoacids were selected), while the least affected was *nad4* (85.57%); *rrnL* and *rrnS* were similarly affected by the masking phase (28.20% and 25.57%, respectively). PartitionFinderProtein suggested the partition of PCGs in two clusters, namely ATP synthase/NADH dehydrogenase subunits (*atp6*, *atp8*, *nad1*, *nad2*, *nad3*, *nad4*, *nad4L*, *nad5*, and *nad6*) and cytochrome c oxidase subunits/cytochrome b (*cox1*, *cox2*, *cox3*, and *cytb*); conversely, PartitionFinder suggested to keep together the two ribosomal genes (*rrnL* and *rrnS*). Best-fitting molecular evolution models were JTT [68], LG [69], and GTR [70], respectively. The consensus tree computed over 1,000 bootstrap replicates is highly supported (Fig 4), being the bootstrap proportion equal to 100% for all nodes, with one exception (the *Paphia* clade). Veneridae were recovered as monophyletic, being the mactrid *Coelomactra antiquata* the sister taxon. Tapetinae and Meretricinae are also monophyletic; within Tapetinae, the deepest split separates the DUI species *R*. *philippinarum* from *R*. *decussatus* + *Paphia*; within Meretricinae, only the genus *Meretrix* is represented in our tree and the available *M*. *lamarckii* mtDNA clusters with our F genome.

### Genetic variability

Nucleotide Jin-Nei distances and aminoacid Kimura distances were calculated between *M*. *lamarckii* F-mtDNA and M-mtDNA for each PCG, tRNA, rRNA, UR and for concatenations of these, up to the whole genome. Jin-Nei and Kimura distance values are reported in Table 4 and S6 Table (for single tRNAs).

**Table 4:** Bivalves nucleotide and aminoacid (boldface) distances.

		Reference	All coding DNA^a^	PCGs^b^	rRNAs	tRNAs	*atp6*	*atp8*^c^	*cox1*	*cox2*	*cox3*	*cytb*	*nad1*	*nad2*	*nad3*	*nad4*	*nad4L*	*nad5*	*nad6*	*rrnL*	*rrnS*
*Anodonta anatina*	F	NC_022803	92.24	90.02	98.76	112.28	114.22	109.70	80.86	88.62	78.97	85.97	90.20	100.78	90.00	96.48	48.03	88.90	119.64	98.99	98.70
	M	KF030963		**81.46**			**87.98**	**186.71**	**42.34**	**82.92**	**54.98**	**54.35**	**66.80**	**133.98**	**98.29**	**150.76**	**92.06**	**101.01**	**194.11**		
*Hyriopsis cumingii*	F	NC_011763	10.09	11.59	5.80	4.85	10.70	4.32	7.04	14.78	9.96	12.80	13.82	16.56	5.59	10.23	5.47	15.95	7.32	5.17	6.76
	M	HM347668		**1.98**			**0.43**	**4.20**	**0.20**	**2.71**	**1.17**	**1.58**	**2.39**	**3.18**	**1.72**	**1.03**	**2.96**	**3.57**	**0.00**		
*Hyriopsis schlegelii*	F	NC_015110	7.52	8.18	3.51	7.17	6.94	11.77	8.58	26.10	4.57	10.03	7.32	6.79	5.46	5.79	3.26	6.26	7.32	4.53	1.93
	M	HQ641407		**1.34**			**1.29**	**7.81**	**0.39**	**3.63**	**0.39**	**1.05**	**2.04**	**0.94**	**0.00**	**1.03**	**1.12**	**1.40**	**2.51**		
*Meretrix lamarckii*	F	KP244451	49.65	55.03	37.86	27.41	61.65	43.82	45.83	59.95	45.08	51.38	41.22	52.56	51.58	58.08	40.76	70.81	62.34	37.47	38.34
	M	KP244452		**19.57**			**13.55**	**9.71**	**12.95**	**43.17**	**15.42**	**17.81**	**4.82**	**13.75**	**18.90**	**9.93**	**16.90**	**33.45**	**28.73**		
*Musculista senhousia*	F	GU001953	64.73	71.53	44.63	45.51	80.66		54.79	71.26	74.24	36.14	72.38	94.98	76.24	82.73	63.26	90.98	73.89	61.15	24.44
	M	GU001954		**23.19**			**27.21**		**5.52**	**43.66**	**17.40**	**6.02**	**25.84**	**39.22**	**23.54**	**21.05**	**29.18**	**34.45**	**31.22**		
*Mytilus californianus*	F	GQ527172	86.82	90.97	73.94	76.00	97.13		61.28	93.29	78.24	79.71	75.76	89.64	106.51	124.15	127.89	105.55	135.27	77.64	69.09
	M	GQ527173		**39.82**			**35.96**		**11.87**	**35.05**	**21.50**	**26.53**	**35.36**	**72.89**	**46.92**	**43.94**	**49.80**	**79.85**	**79.85**		
*Mytilus edulis*	F	NC_006161	62.25	71.85	39.72	32.36	73.33		60.52	64.70	68.87	62.87	72.10	79.52	82.80	80.57	88.82	75.66	82.04	43.20	35.14
	M	AY823623		**16.66**			**17.47**		**4.70**	**11.60**	**9.24**	**19.40**	**14.85**	**27.86**	**12.17**	**16.85**	**16.94**	**20.60**	**56.50**		
*Mytilus galloprovincialis*	F	NC_006886	64.68	73.22	45.18	36.47	74.60		53.95	76.90	69.80	67.62	77.26	92.88	38.73	82.16	87.21	81.51	83.56	45.76	44.43
	M	AY363687		**15.20**			**14.86**		**4.50**	**10.66**	**11.91**	**15.28**	**16.07**	**25.51**	**4.44**	**22.44**	**16.42**	**22.26**	**25.29**		
*Mytilus trossulus*	F	HM462080	65.35	75.63	36.44	36.82	67.12	99.83	55.70	70.59	75.06	66.15	71.69	78.44	89.01	85.48	97.43	91.61	90.81	37.64	34.79
	M	HM462081		**19.12**			**13.84**	**67.99**	**5.86**	**12.11**	**12.61**	**13.61**	**14.24**	**27.31**	**19.65**	**22.44**	**17.58**	**46.48**	**23.50**		
*Pyganodon grandis*	F	NC_013661	102.42	101.92	102.53	117.28	119.56	32.19	75.66	91.70	90.26	108.94	118.28	131.56	149.81	99.09	97.67	101.23	106.48	100.56	106.33
	M	FJ809755		**85.18**			**88.68**	**111.47**	**43.24**	**78.68**	**56.89**	**57.66**	**100.86**	**132.61**	**118.81**	**138.89**	**97.42**	**116.94**	**143.55**		
*Quadrula quadrula*	F	NC_013658	113.61	115.31	100.34	113.18	83.49	377.36	76.67	83.03	84.97	108.53	97.81	112.58	87.45	80.06	120.22	92.09	125.57	109.20	88.44
	M	FJ809751		**84.23**			**88.77**	**n/a**^d^	**40.23**	**62.47**	**61.38**	**52.44**	**93.92**	**141.42**	**79.85**	**223.27**	**109.69**	**103.39**	**175.18**		
*Ruditapes philippinarum*	F	NC_003354	75.33	81.28	59.10	64.07	86.49		63.97	101.96	77.73	74.37	65.14	99.00	80.88	44.73	50.78	84.46	85.83	62.20	54.80
	M	AB065374		**47.04**			**46.36**		**12.46**	**87.16**	**47.50**	**39.61**	**25.59**	**66.01**	**38.69**	**259.28**	**44.58**	**46.13**	**99.67**		
*Solenaia carinatus*	F	NC_023250	96.23	96.22	98.70	107.45	104.10	100.16	94.19	84.21	78.87	115.07	101.08	120.46	101.90	88.66	122.68	91.81	98.03	100.43	96.16
	M	KC848655		**79.16**			**86.13**	**140.88**	**40.45**	**75.17**	**55.77**	**58.77**	**73.80**	**135.10**	**89.38**	**156.15**	**81.05**	**101.35**	**141.96**		
*Utterbackia peninsularis*	F	HM856636	101.63	100.76	102.52	122.15	106.38	100.69	86.15	99.73	90.59	91.34	105.68	121.58	102.85	99.22	149.86	95.98	144.58	103.13	102.13
	M	NC_015477		**86.40**			**95.57**	**186.71**	**41.77**	**79.85**	**58.33**	**60.88**	**78.05**	**137.17**	**112.12**	**193.71**	**101.15**	**108.45**	**175.68**		
*Venustaconcha ellipsiformis*	F	FJ809753	97.71	96.96	103.10	109.14	98.23	85.24	73.69	96.01	87.25	97.83	143.08	106.92	71.45	99.65	142.04	91.67	130.07	101.71	105.26
	M	NC_013659		**83.34**			**81.05**	**137.48**	**38.50**	**68.98**	**50.66**	**52.83**	**96.40**	**137.10**	**90.95**	**150.13**	**103.84**	**113.59**	**231.95**		
Unionoidea^e^ (N = 6)			100.64	100.20	100.99	113.58	104.33	134.22	81.20	90.55	85.15	101.28	109.36	115.65	100.58	93.86	113.42	93.61	120.73	102.34	99.50
				**83.30**			**88.03**	**152.65**	**41.09**	**74.68**	**56.34**	**56.16**	**84.97**	**136.23**	**98.23**	**168.82**	**97.54**	**107.46**	**177.07**		
Amarsipobranchia^f^ (N = 7)			66.97	74.22	48.12	45.52	77.28	71.83	56.58	76.95	69.86	62.61	67.94	83.86	75.11	79.70	79.45	85.80	87.68	52.15	43.00
				**25.80**			**24.18**	**38.85**	**8.27**	**34.77**	**19.37**	**19.75**	**19.54**	**38.94**	**23.47**	**56.56**	**27.34**	**40.46**	**49.25**		
Overall^g^ (N = 15)			72.68	76.03	63.48	67.48	78.97	96.51	59.93	74.86	67.63	71.25	76.85	86.95	76.02	75.81	83.03	78.96	90.18	65.92	60.45
				**45.58**			**46.61**	**94.77**	**20.33**	**46.52**	**31.68**	**31.85**	**43.40**	**72.94**	**50.36**	**94.06**	**52.05**	**62.19**	**93.98**		

^a^ Complete genome excluding untranslated regions.

^b^ Protein Coding Genes.

^c^ *atp8* gene is not annotated in some species. See text for details.

^d^ The sequences are too divergent to compute the Kimura distance.

^e^ Average values of Unionoidea. *Hyriopsis cumingii* and *H*. *schlegelii* were excluded because we are most probably comparing two female genomes; see text for details.

^f^ Average values of Amarsipobranchia.

^g^ Average values of all species.

Between the F- and the M-mtDNA the nucleotide Jin-Nei distance of the complete genome (coding + non-coding) is 53.13, corresponding to a 16.19% divergence. Jin-Nei nucleotide distances are 81.53 (25.81%) for non-coding regions and 49.65 (14.89%) for coding genes. PCG concatenation has an aminoacid Kimura score of 19.57 and, within that, the highest values belong to *cox2* (43.17) and *nad5* (33.45), while lowest values are associated with *nad1* (4.82) and *atp8* (9.71). The average Jin-Nei distance between rRNAs is 37.86; the average Jin-Nei distance between tRNAs is 27.41.

We also compared the two *M*. *lamarckii* mitochondrial genomes obtained in this paper (F and M) to the already sequenced *M*. *lamarckii* mtDNA present in literature (GenBank Accession Number NC_016174). The uncorrected distance (*p*-distance) between this genome and our F genome is 9.81% for all the coding DNA, being 10.67% for all PCGs, 8.17% for all rRNAs and 5.33% for all tRNAs. On the other side, the divergence between this genome and our M genome scored is 14.65% for all coding DNA, scoring 15.76% for all PCGs, 11.43% for all rRNAs and 10.72% for all tRNAs. In both cases, the most divergent gene is *cox2* (15.09% and 21.72%, respectively), whereas the less divergent is *atp8* (4.17% and 9.17%, respectively). The aminoacid Kimura distance was also computed to account for synonymous substitutions (S7 Table): again, the NC_016174 sequence is always more similar to the F-mtDNA than to the M-mtDNA and the most divergent gene is *cox2* (19.28 and 39.86, respectively), while the less divergent genes are *atp8* (0.00 and 8.13) and *nad1* (0.67 and 4.12). Generally speaking, with the exception of *cox1* and *cox2*, the Kimura distance from M-mtDNA is always one order of magnitude higher than from F-mtDNA.

The F vs M distances were also computed for all known DUI species whose complete mitochondrial genomes have been published (see Table 4). *M*. *lamarckii* has lower divergence values, when compared to other DUI families such as Unionidae or Mytilidae. The Unionidae show, by far, the highest values, with divergence scores of coding DNA ranging from 92.24 of *Anodonta anatina* to 113.61 of *Quadrula quadrula*; *Hyriopsis* species show abnormally low distance values, not even comparable with average Unionidae family values. Unionidae are followed by Mytilidae with an all-coding Jin-Nei distance comprised between 62.25 of *Mytilus edulis* and 86.82 of *M*. *californianus*. Within the Veneridae, *Venerupis philippinarum* has a higher divergence value (75.33) with respect to *M*. *lamarckii* (49.65). The most divergent PCGs are *atp8*, *nad4*, and *nad6* with average Kimura distances of 94.77, 94.06, and 93.98, respectively; the most conserved is *cox1* (20.33).

**Fig 5:**
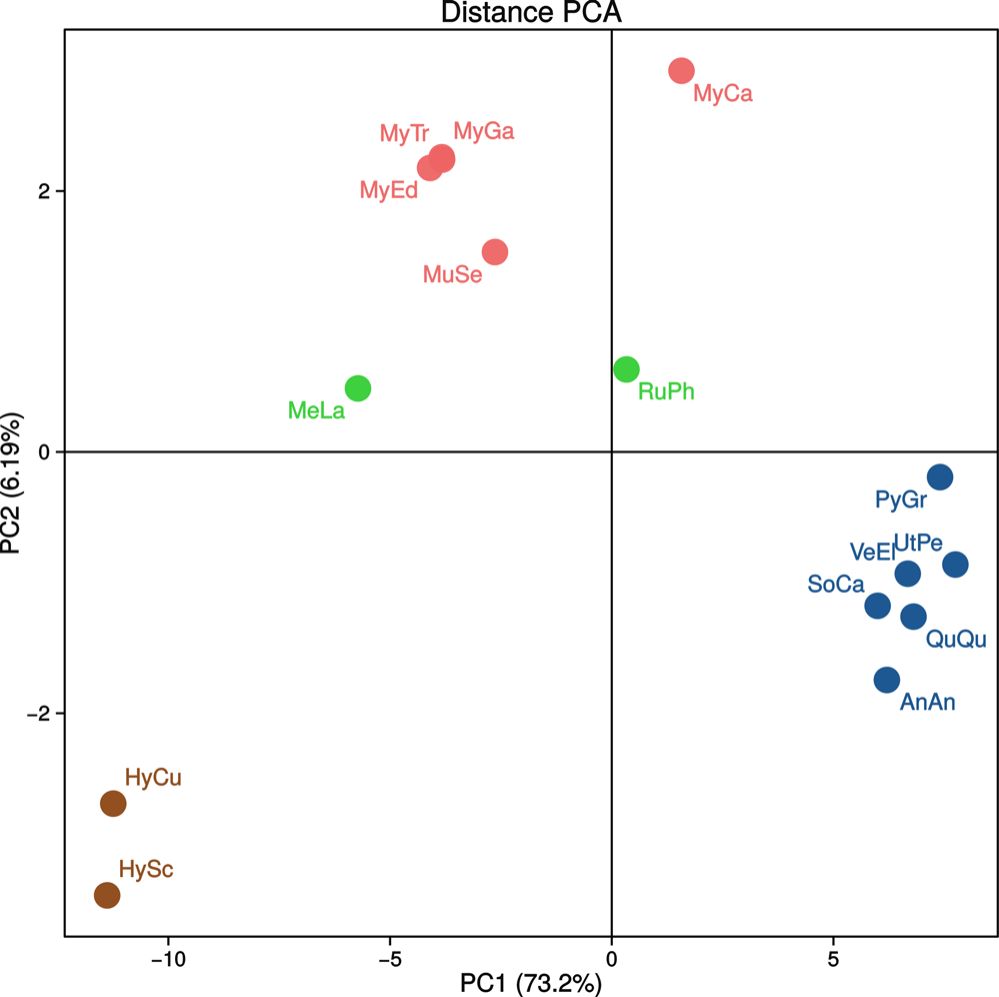
PCA plot. Principal Component Analysis (PCA) based on both Jin-Nei and Kimura distances reported in Table 4. Colors refer to different families: blue, Unionidae with the exception of *Hyriopsis* spp. (brown; see text for details); Indian red, Mytilidae; green, Veneridae. AnAn, *Anodonta anatina*; HyCu, *Hyriopsis cumingii*; HySc, *Hyriopsis schlegelii*; MeLa, *Meretrix lamarckii*; MuSe, *Musculista senhousia*; MyCa, *Mytilus californianus*; MyEd, *Mytilus edulis*; MyGa, *Mytilus galloprovincialis*; MyTr, *Mytilus trossulus*; PyGr, *Pyganodon grandis*; QuQu; *Quadrula quadrula*; RuPh, *Ruditapes philippinarum*; SoCa, *Solenaia carinatus*; UtPe; *Utterbackia peninsularis*; VeEl, *Venustaconcha ellipsiformis*.

The resulting PCA plot (Fig 5) uses the first two components to explain the 73.20% + 6.19% = 79.39% of distance variability (using Jin-Nei and Kimura distances together). Datasets are roughly arranged by overall divergence levels along the first principal component (*Hyriopsis* spp. < Mytilidae+Veneridae < Unionidae); the second principal component further separates venerids and mytilids from unionids. Finally, it is impossible to reject the null hypothesis that nucleotide distance rankings among single PCGs in Unionidae and Mytilidae + Veneridae are unrelated, using both the Spearman ρ (*p* = 0.2392) and the Kendall's τ (*p* = 0.2044). For example, *cytb* and *nad1* are highly divergent for Unionidae, but they are among the least variable for Amarsipobranchia, while the opposite is true for *nad4* and *nad5*.

## Discussion

### Comparison with the previously published *Meretrix lamarckii* mitogenome

The two mitochondrial genomes of *Meretrix lamarckii* (F and M) sequenced here are slightly shorter than the one previously reported in GenBank: 20,025 (F) and 19,688 (M) bp against 21,209 bp [18]. This genome was extracted from a somatic tissue (the foot muscle; [18]) and, indeed, it was previously attributed to the female type by Plazzi and colleagues [10]. The phylogenetic analysis of the present work further corroborates this hypothesis (Fig 4). It shares the same gene content and gene order, with the exception of *trnL*(AAG), *trnQ*(UUG) and *trnF*(AAA), which have been found only in M-mtDNA. This, again, strengthens the idea that the previously published genome is from the female lineage.

However, comparison between either sex and the published F genome showed surprisingly high divergence values. These were generally one order of magnitude higher when comparing it with our M genome, and, as in the case of our F/M comparison, divergence ranking is similar: f.i., highest values are obtained from *cox2*, while lowest scores are obtained from *atp8*. Significant divergence values are still observed at the aminoacid level for both sexes; the distances between our M-mtDNA and the published F genome are comparable to those computed between the two sexes in the present study (Table 4 and S6 Table).

It is possible to find similarities in the LUR structure between the two F genomes, a highly folded region being the divide between a supernumerary ORF and a region with tandem repeats of about 100 bp in length. However, in the published F genome it was not possible to find a functional ORF (S9 Fig). This may be due to sequencing errors; if the available sequence is confirmed, it is hard to say whether an ORF was originally present in the species and was subsequently pseudogenized in some populations or a novel ORF appeared in some others. Given the widespread presence of supernumerary mitochondrial ORFs in bivalves [71, 62, 72, 63, 5, 73–74], we largely favor the first hypothesis. On the other side, it is possible to align the 3' repeated motifs (S10 Fig). The great variation in length between the two LURs is due to the different number of repeats: 2 in our F genome, 13 in the published one. This difference, in turn, accounts for the aforementioned difference in length between the two genomes.

Interestingly, intra-specific variability in the number of repeats in a mitochondrial LUR has been reported elsewhere [75–76] for (DUI) bivalves. The two *M*. *lamarckii* specimens sampled for this research come from the Tokyo area (Japan), whereas the F genome available in GenBank comes from Zhejiang (China) [18]. Recall that the Chinese specimen was only tentatively identified as *M*. *lamarckii* due to its similar morphology, despite showing some differences in color, shell shape and thickness [18], we cannot completely rule out the hypothesis that these specimens belong to different species; however, it is not unconceivable that the differences found here simply reflect the high degree of taxonomic distinctness between Japanese and Chinese clams belonging to very distant populations.

### Nucleotide composition and codon usage

A-T content in *M*. *lamarckii* F and M genomes is slightly higher with respect to the average A-T content in bivalves, but it is comparable with those of other DUI organisms such as *V*. *philippinarum* and *M*. *senhousia* [63]. The coding strand (+) is G-T rich: this is expected [77–78] and in good agreement with [63], where it was stated that a higher G-T percentage is related with mtDNAs characterized by most (if not all) genes located on the same strand.

Codons usage reflects the general nucleotide composition of the two genomes, with a high presence of T in most used codons. In almost all cases, except for *trnL*(TAA), *trnK*(TTT), and *trnM*(CAT) in F-mtDNA and for *trnL*(TAA), *trnF*(AAA) and *trnK*(TTT) in M-mtDNA, within four-fold or two-fold degenerate codon families the most used codons do not have a complementary anticodon in mitochondrially-encoded tRNAs (Table 3). Moreover, they differ for only one base (the third one) with respect to the synonymous codon for which a complementary tRNA exists in the mitochondrion. The codon usage table demonstrates the presence of high degrees of third-base wobbling in *M*. *lamarckii*, as previously seen in other bivalves [8, 63]: a tRNA can have a non-standard base at the first anticodon position pairing with more than one base and allowing to bind codons that are not perfectly complementary.

### PCGs and the *cox2* insertion

With the exception of the supernumerary ORF, all genes are located on the same strand in both F and M mtDNAs of *M*. *lamarckii*. This is commonly found in all Amarsipobranchia, while unionids [71, 9, 63] and *Solemya velum* [63] encode genes on either strand. This finding reinforces the hypothesis that the one of *M*. *lamarckii* is a derived state, which evolved once in the common ancestor of Pteriomorphia and Heterodonta ([9]; and reference therein).

The *atp8* gene was declared as missing in several bivalve species [8], especially in the genus *Mytilus* [79], even if it was recently found in some bivalves like *Solemya velum* [63], *Musculista senhousia* [8], *Venerupis philippinarum* [80], and presently in *M*. *lamarckii*. In addition, recent studies [9, 81] found this gene in species in which it was not previously annotated.

The use of Jin-Nei corrected distance to evaluate nucleotide divergence unveiled that *atp8* is not less conserved than other mitochondrial genes (see Table 4). As suggested elsewhere [5, 82], there is a strong possibility that the absence off this gene is simply due to past annotation difficulties or inaccuracy. Up to now, the presence/absence of ATPase subunit 8 does not appear linked with DUI [9], but rather, if confirmed, to phylogeny [63].

It was suggested to be the commonest situation in metazoans that the two ATPase subunits, *atp8* and *atp6*, are adjacent and overlapping [79]. This especially holds if the co-translation of these genes from a bicistronic transcript (as is the case in mammals; [83]) is confirmed as a widespread rule. In fact, this association is present in the Unionidae [71] and it was recently found in *S*. *velum* [63]; however, a disjointed location of *atp8* and *atp6* has already been highlighted for some heterodont bivalves, like *Hiatella arctica* [80] and *Macoma balthica* [82]. Similarly, in *M*. *lamarckii atp8* and *atp6* are not neighboring in either F- and M-mtDNA: again, the contiguity of these genes may be an example of an ancestral state that was subsequently lost in derived bivalves.

M *M*. *lamarckii* genome presents an insertion of 100 codons in *cox2* gene, which is totally absent in the F counterpart (Fig 2). It is not the first time that a M-*cox2* gene is longer than F-*cox2*; generally, however, these extensions map to the 3’ end of the gene. In fact, the M-*cox2* 3’ tail is present in all three subfamilies of Unionidae [5]. This extension (M*cox2*e) has been found only in M mtDNAs and varies in length between 177 and 192 bp [84–85]. M*cox2*e has been found in poly-adenylated transcripts of *cox2* obtained from male gonads, and also proved to be translated and localized in both inner and outer mitochondrial membranes [84–86]. The structural analysis of unionid M*cox2*e sequences reveals the presence of the two canonical N-terminal trans-membrane helices (TMHs). In addition to that, several additional TMHs were found in M*cox2*e [87]. For the above mentioned reasons, a proposed hypothesis was that such extension may be a mitochondrial tag implicated in male mitochondria survival to elimination and differential segregation during development [87].

Outside from the unionid family, the pattern of *cox2* variations among DUI M and F lineages is unclear and not easy to unravel. In mytilids, no extensions were found in M genomes of *Mytilus* [5], but a duplicated *cox2* gene (*cox2b*) is found in M *M*. *senhousia* [8], with the duplicated gene being longer than the original one at 3’. A putative TMH of 41 residues was found in the *cox2b* tail [8], allowing the authors to hypothesize a correlation between the unionid M*cox2*e and the *cox2b* tail of *M*. *senhousia*.

*V*. *philippinarum* is with *M*. *lamarckii* the only known DUI species of the family Veneridae: a duplication of the *cox2* gene, similar to that of *M*. *senhousia* (i.e. longer at 3’), was found, but, contrastingly, it is located in the F genome [8]. However, additional TMHs (either in insertions or tails) are not detectable in *V*. *philippinarum cox2*, nor in *M*. *lamarckii* (S11 Fig). Moreover, @TOME analysis did not find any intron, and the coding frame is apparently kept (see Fig 2).

Concluding, it was impossible to properly assign a function *in silico* to this region, and further analyses are therefore mandatory in this regard. Non-canonical features in *cox2* gene are often coupled with DUI, but a general rule is still not evident and each DUI system seems to follow its own evolutionary pathway. However, despite the relationship between *cox2* variations and DUI phenomenon has not been demonstrated yet, the finding of a new M-*cox2* gene insertion (albeit differently located in the gene) in another DUI bivalve is an interesting clue.

### Supernumerary tRNAs in M-mtDNA

As mentioned above, M and F genomes basically share the same gene arrangement, the only difference being three tRNAs in M-mtDNA. As a consequence of the high variability of their mitochondrial genomes, additional tRNA copies are common in bivalves [88–90]. In fact, when aligning the M additional tRNAs with the region mapped in the same position in the F-mtDNA, high levels of sequence similarity were always detected (see S5 Fig). Therefore, we may hypothesize that the duplication of *trnL*, *trnQ*, and *trnF* took place before the separation of the two sex-linked lineages, and that, afterwards, the F copies became pseudogenes or remain functional tRNAs that the *in silico* methods are not able to retrieve. Anyway, it has also to be noted that the anticodon region of the F counterpart of M-*trnQ*(TTG) would be complementary to the stop codon TAA.

The presence of a tRNA in the middle of M-LUR (*trnF*(AAA)) is intriguing and deserves further investigation: possibly, the cloverleaf structure of a tRNA was co-opted as part of the signaling structure of the putative control region (see below) and, thus, would not correspond to a functional tRNA. However, it is noteworthy that the anticodon of the middle-LUR tRNA is AAA, which is complementary to TTT, the most used codon in both genomes (see Table 3).

The presence of a functional tRNA in the middle of a control region, where it may work also as a signaling sequence, would make of the *trnF*(AAA) gene of M-mtDNA of *M*. *lamarckii* a good example of an evolutionary spandrel [91] and/or a case of molecular exaptation: this region, being a tRNA, necessarily had a complex secondary structure, and this became useful in the wider context of the control region as well (or *vice versa*, even if the presence of a degenerated tRNA in the F-mtDNA makes us to prefer the first hypothesis). The presence of a tRNA-like structure was already signaled by [67] in the *Mytilus* spp. LUR, but in the case of *M*. *lamarckii* it seems that the tRNA maintained its functionality.

Other expected non-canonical tRNA structures are found in our genomes: f.i., in both F- and M-mtDNA two *trnS* were found and the DHU arm was not recovered in *trnS*(UCU). However, as mentioned by [8], this unusual tRNA has been found in several other animal groups and it evolved early in Metazoans group [92]. *In vitro* analysis further confirmed its functionality [93].

### Control Region (CR) and the Origin of Replication (OR)

Several parameters have been proposed to identify the mtDNA control region (CR). The most used are the presence of repetitive elements, palindromes, length, high A-T content and secondary structures with T-rich loops [67, 94, 71]. *M*. *lamarckii* Long Unassigned Region (LUR) is the longest UR in both F and M mtDNAs, although the M one is apparently split into two parts by a phenylalanine supernumerary tRNA (as mentioned above). A-T content is roughly the same found in the entire genomes, 64.2% for F and 65.8% for M, even if several poly-T have been found during (and heavily hampered) sequencing.

The short (15-bp long) repeat is essentially a stretch of G and A and may simply reflect the general G-T-/A-T-richness of both genomes in a region where less selective constraints are working; however, this repeat is conserved in both F and M genomes and it is known that similar G-rich sequences are present in *Mytilus* and human control regions, being related with replication and/or transcription [67].

The 109-bp long repeats are located near to the 3' end of the F-LUR sequence, and, due to their proximity with the putative origin of replication (see below), they may play a functional role in F mtDNA duplication (but recall that they are not detectable in M). Both M- and F-LUR present a central region which appears to be heavily folded (S6 Fig): again, this secondary structure may play some role for the replication/transcription process to begin [67, 71].

The nucleotide composition at fourfold degenerate sites is related with single-strand state duration during mtDNA replication. As detailed in ([95–98, 71]; and reference therein), the more the heavy (H) strand remains unpaired, the more the spontaneous hydrolytic deamination of C to U and A to hX (hypoxanthine) takes place. Such an increase of T and hX in the H strand leads to a corresponding increase in the percentages of A and C in the complementary lagging (L) strand where the H strand remains for longer time in the single-stranded condition, i.e. near to the OR. Moreover, single-stranded-guanine may spontaneously oxidize to 8-hydroxyguanine, which basepairs with adenine: thus, in this case, G decreases and T increases on the H strand. In a nutshell, T will only tend to accumulate near to the origin of replication of the H strand, while the opposite is true for A and C; finally, G may behave in either way [95, 98]. This asymmetrical composition can leave a neutral signature in fourfold degenerate sites, being them under no or weak selection.

The 700-bp sliding window analysis on these sites is in agreement with this model (Fig 3): with the exception of A (and of T in F-mtDNA), all correlations are significant, even if R^2^ values are very low (<25%). Setting *cox3* as the starting point of the pattern, T in M-mtDNA tends to decrease, C tends to increase, and G decreases in the F-mtDNA and increases in the M one, which would be expected if the OR is located upstream to *cox3*. This also point to the conclusion that the "+" strand is in fact the H strand (as predictable, being all genes located on it).

The A-T skew at four-fold degenerate sites is known to be correlated with the position of the ORs as well: extreme (i.e., closer to ±1) values are associated with PCGs located near to the OR of the H strand, while balanced (i.e., closer to 0) values are associated with PCGs located near to the OR of the L strand [95, 97, 99]. Given the overall high T-richness of these genomes, the A-T skew at four-fold degenerate sites is always negative: however, lowest values (i.e., closer to −1) are associated with the LUR and with the *cox3* gene (Fig 3B), while highest values (i.e., closer to 0), are associated with the *nad2*/*nad4L* and *atp6*/*nad3* regions.

Therefore, we have further evidence that the LUR contains the OR of the H strand; moreover, it is tempting to conclude that either the *nad2*/*nad4L* or the *atp6*/*nad3* region is the OR of the L strand. Both regions are neighbored by a two- or three-tRNA cassette, and it has been shown that an array of tRNAs on a strand may act as OR in the opposite one through alternative secondary structure [100]. If the OR of the L strand were located in the *atp6*/*nad3* region, that shows A-T skews closer to 0 (Fig 3B) and is near to three tRNAs in either sex (Fig 1), this would leave the OR of the L strand quite distant from the OR of the H strand, a situation very similar (if not more extreme) to that of *Mytilus* [98] and unionids [71]. However, it is possible that more complex patterns are shadowed by the presence of all genes on the same strand and by the high T-richness of both genomes (recall that the A-T skew for the third codon position of PCGs is −0.44 for both genomes; Table 2).

As a conclusion, we gathered seven pieces of evidence that the F-LUR and the M-LUR are the control regions of *M*. *lamarckii* mtDNAs; as detailed above, most of these features are shared with other DUI species, namely *Mytilus* spp. [67, 98] and Unionidae [71]. First of all, we have (i) a complex secondary structure, that, if the supernumerary ORFs are expressed (see below), would involve the complete LUR. Within that, we found, approximately from 5' to 3': (ii) the presence of G-rich elements; (iii) the presence of a tRNA (only in M); (iv) a sequence with some homology to the human TAS element (only in F); (v) the 109-bp long repeats (only in F). Finally, downstream from the LUR, we detected (vi-vii) the two above-mentioned nucleotide composition trends. Being these features unique to this region, we propose the LUR to act as the CR and to contain the OR of the H strand.

### Supernumerary ORFs

Many DUI species, like *M*. *senhousia* and *Mytilus* spp., present supernumerary ORFs with no known homologies with other proteins (i.e., ORFans; [101]), which are located in the LUR. *M*. *lamarckii* is no exception. For such ORFs a correlation with the DUI phenomenon has been suggested [71–72, 5], even if the opposite was also proposed, interpreting the RNA transcripts as degradation intermediates [102]. Supernumerary ORFs were also found in the basal species *S*. *velum*, leading to the hypothesis that they constitute a plesiomorphy among bivalves [63]. Although some of these ORFs have uncontrovertibly proved to be translated [71–73], it is uncertain whether the *M*. *lamarckii* ones are even transcribed. This issue can be assessed only by looking at expression data, but, currently, without an available transcriptome/proteome of *M*. *lamarckii*, we cannot confirm nor disprove the functionality of either ORF.

However, a precise homology between F and M ORFans was detected, which would not be expected if these sequences did not share a common ancestor; furthermore, an ORFan with high homology to F_ORF141 has been found in all species belonging to genus *Meretrix*. Interestingly, this would make of this supernumerary ORF the only gene located on the reverse strand in all the *Meretrix* genomes (S5 Table).

### Sex-linked mtDNA diversification and evolution in DUI bivalves

The two entire *M*. *lamarckii* genomes diverge by a 16% on average (see also [10]), hence the divergence between F and M mtDNAs is somewhat lower than other DUI species. On the other hand, the most diversified genomes belong to unionids (around 35%), followed by mytilids (around 25%). The other venerid, *V*. *philippinarum*, shows levels of divergence comparable to those of mytilids (26%).

In this work, nucleotide Jin-Nei and aminoacid Kimura distance values of all DUI species (whose complete mitochondrial genomes are available in GenBank) were calculated between M- and F-type mitogenomes to estimate divergences and give an idea of the rate of independent evolution between the two sex-linked genomes. We strongly advocate the use of corrected distance methods, like the Jin-Nei and Kimura formulae, over the uncorrected *p*-distance, because of the high divergence between sex-linked mtDNAs in many DUI species and the overall high variability of the molluscan mitochondrial genome, where significant level of saturation and multiple-hits events are quite common (see, f.i., [61]).

Both *Hyriopsis cumingii* and *H*. *schlegelii* Jin-Nei distance values are surprisingly low, in contrast with all other sequenced unionids (and, in general, DUI) mtDNAs. However, there is a chance that there was an error in assigning the paternal route of transmission to genomes retrieved from males. Actually, as reported in GenBank, *H*. *cumingii* M genome (GenBank Accession Number HM347668) was indeed extracted from mantle tissue, whereas the source of the F (GenBank Accession Number NC_011763) is not reported. Conversely, *H*. *schlegelii* F and M genomes (GenBank Accession Numbers NC_015110 and HQ641407, respectively) were extracted by gonad tissue–not from gametes–which is known to contain somatic cells carrying the F genome. Furthermore, the study of *cox2* gene reveals that M mtDNAs of both species do not have additional putative TMHs typical of all other unionids M-*cox2* (see above; S11 Fig). These evidences point to the fact that both genomes do belong to F-type and their minimal divergence is due to normal intraspecific variability.

More interestingly, in the PCA of distance scores (Fig 5), DUI species clustering follows the taxonomic arrangement of bivalves. Two large assemblages are visible: unionid species, one side, and Amarsipobranchia (i.e. Veneridae + Mytilidae), the other. In fact, the divergence of the two mtDNAs is higher in unionids (Table 4). This may point to the conclusion that DUI is somehow different in these two lineages, leading to distinct patterns of sequence evolution. This is not a new observation, since differences in many respects of DUI were repeatedly evidenced between Unionidae and Mytilidae + Veneridae (see, e.g., [5, 14]; and reference therein). In particular, the main difference is that unionids have established M- and F-mtDNA lineages earlier than species radiation, thus leading to a higher divergence between sex-linked lineages and, thus, to a very strict "gender-joining" phylogenetic pattern [84–85, 5].

Conversely, in Amarsipobranchia, two tentative, perhaps overlapping explanations were given to account for the observed "species-joining" pattern, with the only exception of the fairly recent *Mytilus edulis* species complex ([5]; see also Fig 4 for Veneridae): (i) multiple role reversal events, as well as reversions to SMI, may have blurred the phylogenetic and diversification pattern [103–104, 5], and/or (ii) DUI and the establishment of the two sex-linked mitogenomes may have happened many times in different lineages. This was a conceivable hypothesis given the model described in [105, 5], where a relatively simple switch, called factor Z, is proposed to trigger DUI/SMI swaps.

However, it is also worth pointing out that genetic divergence behaves differently in single-genes pairwise comparisons, and this is not expected if we consider the observed variability as a function of the DUI onset time only. For example, unionids show high distance values for *cytb* and *nad1*, which are among the most conserved within Amarsipobranchia, and *vice versa* for *nad4* and *nad5* (Table 4). Currently, it is not possible to speculate on the reasons of such divergence patterns, and more comparative and structural analyses have to be done.

## Conclusions

The present phylogenetic reconstruction (Fig 4) corroborates previous evolutionary trees of venerids [106, 10] and, above all, indicates future research lines: the detection of DUI in other genera of the family Veneridae and/or in other species of the genus *Meretrix* would add consistency in the single DUI origin hypothesis (at least for Heterodonta; [14]), while the direct observation of SMI in those groups would probably lead to a re-evaluation of the parsimony approach to the origin of DUI proposed in [14]. Furthermore, investigating the distribution of DUI within the genus *Meretrix* would open the field for comparisons with the *Mytilus* species complex, which is the only known case of a gender-joining pattern among Amarsipobranchia.

The great genome variability shown by bivalves at the mitochondrial level may somehow veil mtDNA similarities between distantly related DUI species, so that comparisons between taxonomically closer DUI species are needed to further characterize and understand the DUI mechanism and the related molecular machinery. This opportunity was unavailable for venerids so far. Therefore, the sequencing and characterization of *M*. *lamarckii* mtDNAs presented here makes this species a useful experimental counterpart of *V*. *philippinarum*, which in turn has been thoughtfully characterized in recent years (see, f.i., [107–111, 74]).

## Supporting Information

S1 Fig Stem-loop secondary structures of F and M *Meretrix lamarckii*. Inferred stem-loop secondary structures of all Unassigned Regions (URs) comprised between two neighboring protein coding genes (PCGs). The label of each structure is obtained by concatenating "UNs" (Unassigned Nucleotides) and the two PCG names.
(PDF)

S2 Fig *Meretrix lamarckii* F and M rRNA secondary structures. A, F-*rrnL*; B, F-*rrnS*; C, M-*rrnL*; D, M-*rrnS*.
(PDF)

S3 Fig *Meretrix lamarckii* F tRNA secondary structures. All aminoacids are reported with their one-letter code; anticodons are highlighted in yellow.
(PDF)

S4 Fig *Meretrix lamarckii* M tRNA secondary structures. All aminoacids are reported with their one-letter code; anticodons are highlighted in yellow.
(PDF)

S5 Fig Alignments of M additional tRNAs and corresponding F Unassigned Regions (URs). The M anticodons are highlighted, while stretches of nucleotides involved in tRNA stem-loop structures are underlined. Only the relevant part of the corresponding F-UR is shown.
(PDF)

S6 Fig *Meretrix lamarckii* F and M Long Unassigned Regions. F (A) and M (B) *M*. *lamarckii* Long Unassigned Region (LUR) inferred secondary structures. The 109-bp tandem repeat that was detected in F-LUR is detailed in the upper-right insert; yellow lines, first repeat; purple lines, second repeat.
(PDF)

S7 Fig Alignment of the F Large Unassigned Region (F-LUR) and the human Termination-Associated Sequence (TAS) element. Numbers refer to the positions on the mitochondrial genomes. The TAS element was taken from [66] and located on the revised Cambridge Reference Sequence (GenBank Accession Number NC_012920).
(PDF)

S8 Fig Autocorrelograms. Autocorrelograms for nucleotide trends shown in Fig 3: the autocorrelation function (acf) is plotted for lags from 0 to 17. Page 1, female mitochondrial genome; page 2, male mitochondrial genome; dashed lines, large-lag 95% standard errors.
(PDF)

S9 Fig Female unassigned ORFs. Alignment between the unassigned ORF found in the LUR of the female mitochondrial genome (MeLaF) and the corresponding region of the published *Meretrix lamarckii* mitochondrial genome (MeLaNC_016174); numbers refer to positions on the GenBank sequences. Regions of mutations pseudogenizing the putative lost ORF are shaded in black in the published sequence; asterisks mark identical nucleotides.
(PDF)

S10 Fig Female repeated motifs. MUSCLE alignment between the 109-bp repeated motif of the female LUR (MeLaF) and the 100-bp repeated motif of the published LUR (MeLaFNC_016174). Asterisks mark identical nucleotides.
(PDF)

S11 Fig Transmembrane helices (TMHs) of F- and M-*cox2* of all DUI species. Phobius predictions of *cox2* residue locations for all DUI species used for this work. The *cox2* length is reported in the *x* axis; the *y* axis refers to the posterior probability of a given position to be part of a TMH (gray), cytoplasmic (green), non-cytoplasmic (blue), or part of a signal peptide (red).
(PDF)

S1 Script R script used to compute nucleotide composition and A-T content at four-fold degenerate sites over a sliding window. The script is called 4F; example files and a tutorial are also provided. The same script can be downloaded at the GitHub repository https://github.com/mozoo/4F.git.
(GZ) Click here for additional data file.

S1 Table Primers used to amplify *Meretrix lamarckii* F and M mitochondrial genomes. Primers are listed by pairs, showing for each pair the forward (F) and the reverse (R) primers in the column "Strand". In the column "Sex" it is specified if a given pair was used for the female genome (F), for the male genome (M), or for both (both). For amplicons > 2,000 bp the *Herculase* enzyme was used (see text for details). Where two annealing temperatures are listed, the first one refers to the female genome and the second one to the male genome.
(PDF)

S2 Table Phylogenetic dataset. Sequences in boldface were obtained for this study. Taxonomy is taken from GenBank.
(PDF)

S3 Table *Meretrix lamarckii* F (A) and M (B) Unassigned Regions (URs).
(PDF)

S4 Table Genes located by Glimmer3 software in F and M mtDNAs. The canonical 13 PCGs (bold) and all other Open Reading Frames (ORFs) are reported along with their start base, stop base, frame, and Glimmer score. F_ORF141 and M_ORF138 are shown in bold as well.
(PDF)

S5 Table Putative supernumerary ORFs in *Meretrix* spp. Homologies of F_ORF141 with ORFs in other *Meretrix* mitochondrial genomes; the first entry is F_ORF141 itself.
(PDF)

S6 Table *Meretrix lamarckii* single tRNA Jin-Nei distances. (PDF)

S7 Table Present study vs published genome Kimura distances. The Kimura aminoacid distance is listed for each PCG. F, female genome; M, male genome; NC_016174, published *Meretrix lamarckii* mitochondrial genome. The F-*atp8* gene has 11 aminoacids at the 5' end that are lacking in the published *atp8* gene; as the remaining part of the peptide sequence is identical, the pairwise deletion led to a Kimura distance of 0.
(PDF)
