# Supplementary figures and images for: The Complete Female- and Male-Transmitted Mitochondrial Genome of Meretrix lamarckii
Source: PLoS One. 2016 Apr 15;11(4):e0153631. doi: 10.1371/journal.pone.0153631 (PMC4833323; doi:10.1371/journal.pone.0153631)

A

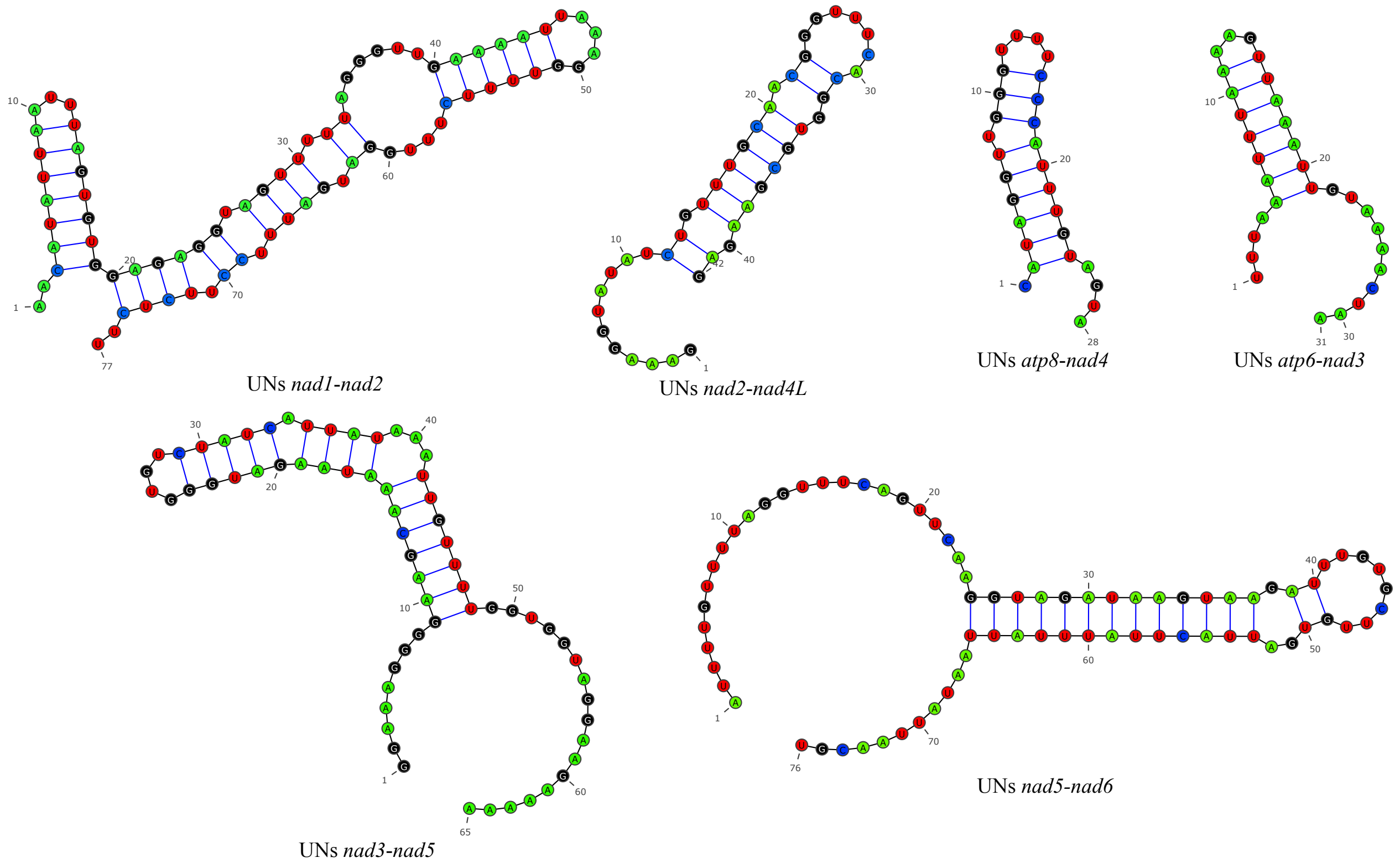

B

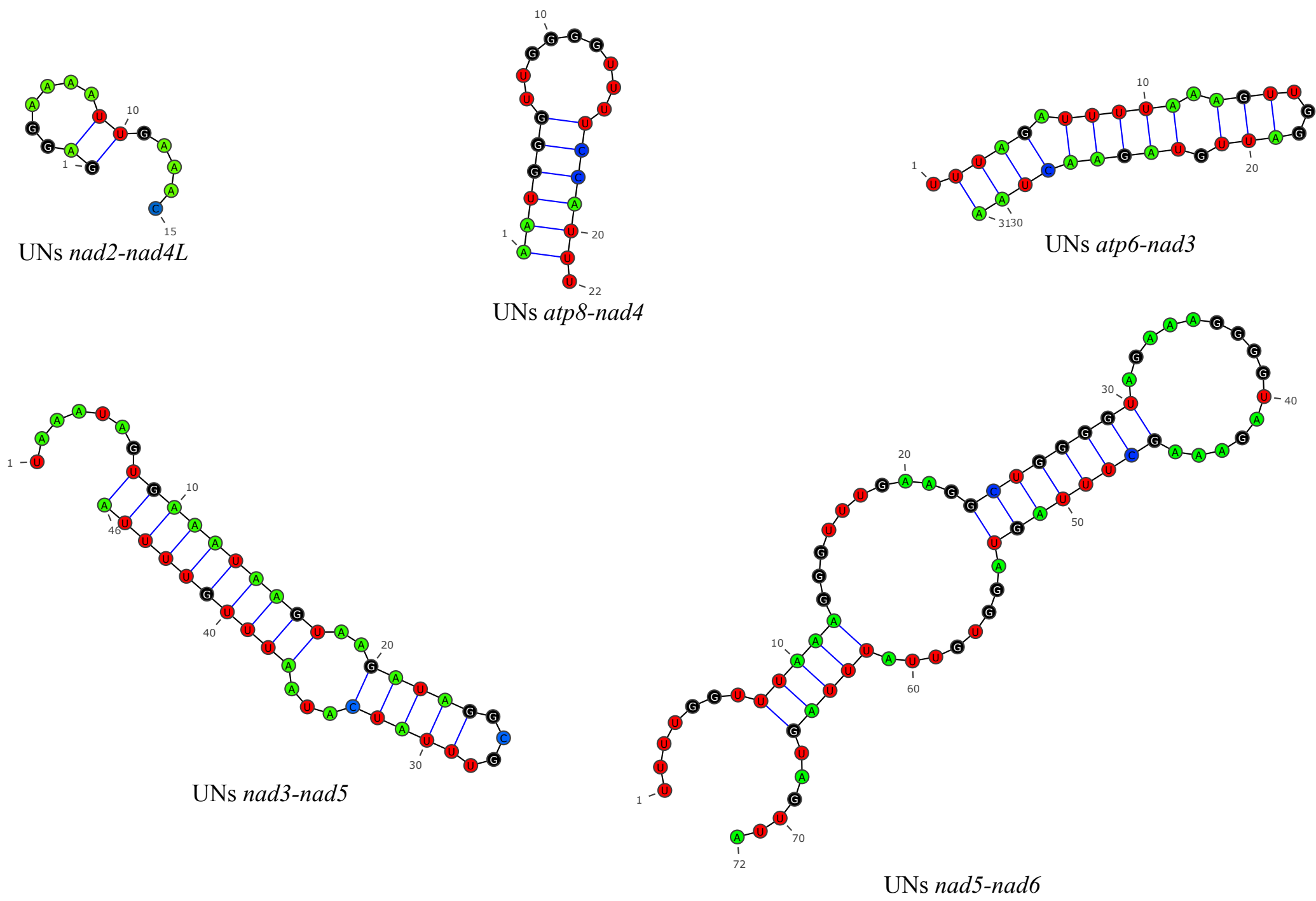

Supplement: S1 Fig — Inferred stem-loop secondary structures of all Unassigned Regions (URs) comprised between two neighboring protein coding genes (PCGs). The label of each structure is obtained by concatenating "UNs" (Unassigned Nucleotides) and the two PCG names. (PDF) [file pone.0153631.s001.pdf]

(A)

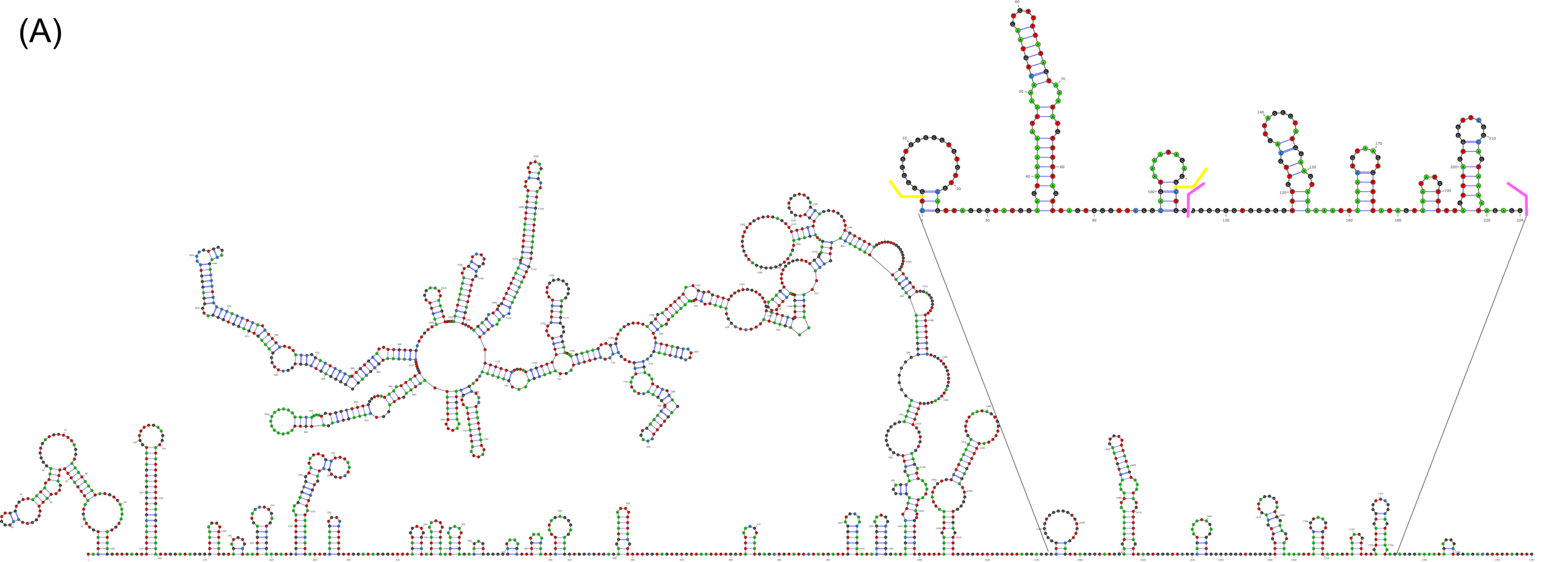

(B)

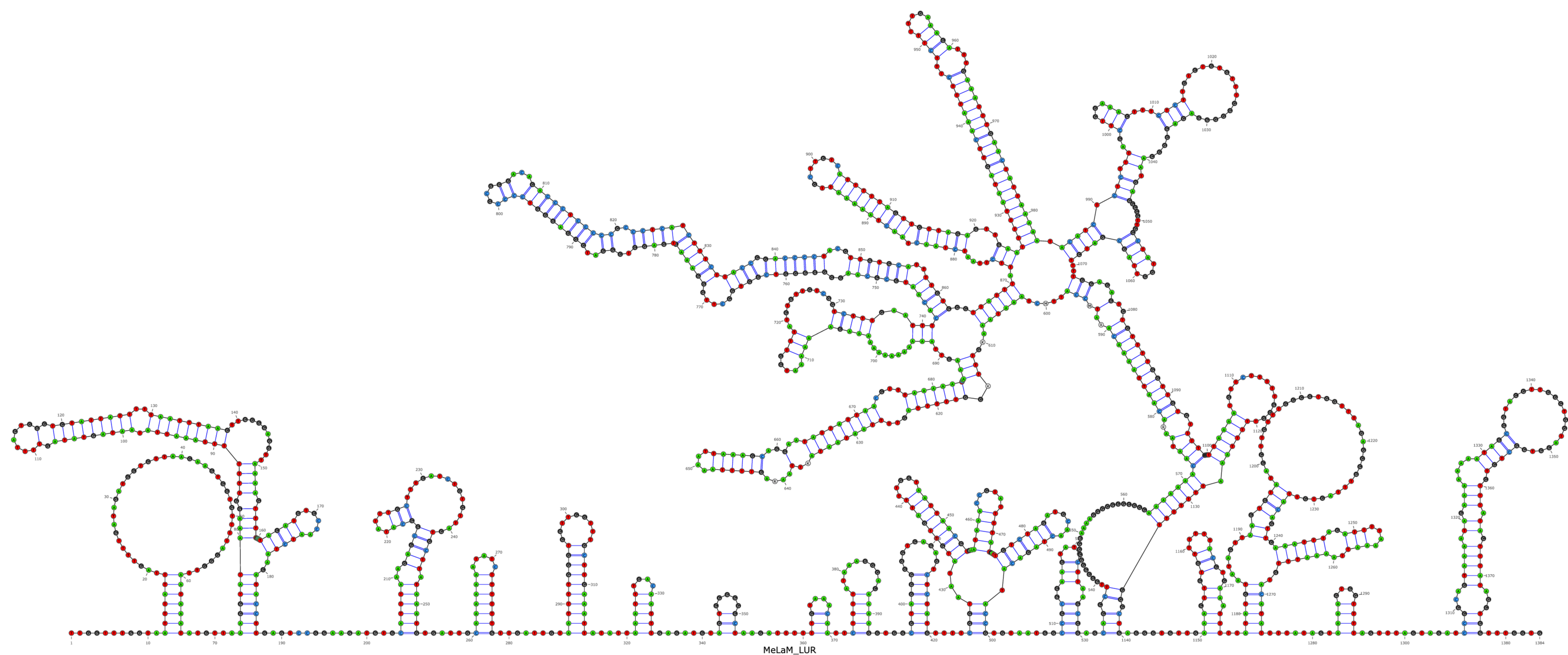

Supplement: S6 Fig — F (A) and M (B) M. lamarckii Long Unassigned Region (LUR) inferred secondary structures. The 109-bp tandem repeat that was detected in F-LUR is detailed in the upper-right insert; yellow lines, first repeat; purple lines, second repeat. (PDF) [file pone.0153631.s006.pdf]

# Autocorrelograms for MeLaF

## Adenosine

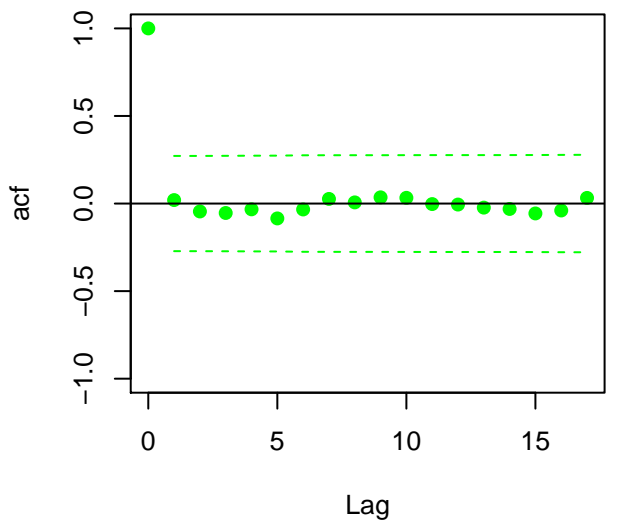

## Cytidine

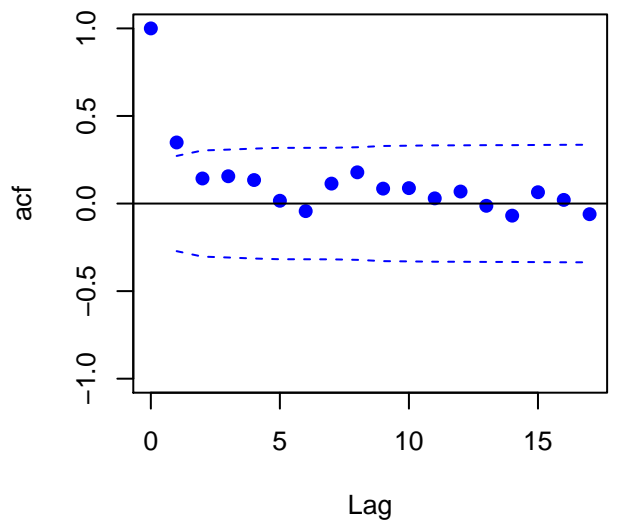

## Guanosine

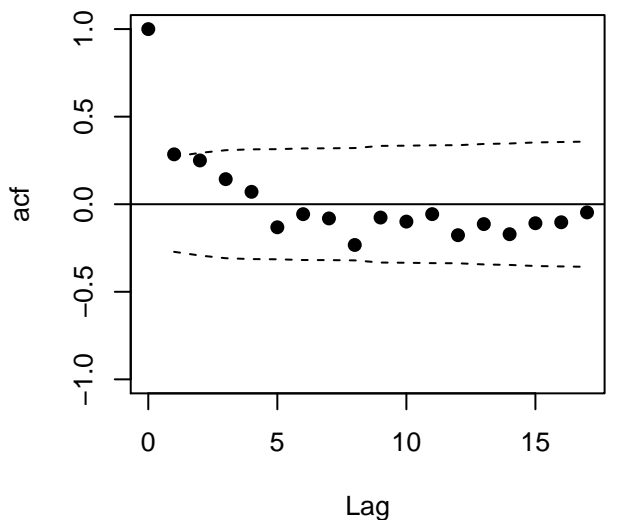

## Thymidine

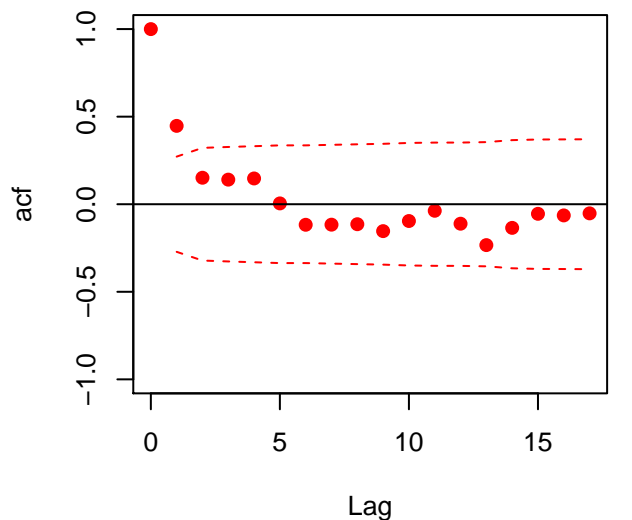

Supplement: S1 Script — The script is called 4F; example files and a tutorial are also provided. The same script can be downloaded at the GitHub repository https://github.com/mozoo/4F.git. (GZ) [file pone.0153631.s012.gz › MeLaF_acf.pdf]
