# Supplementary material for: The Complete Female- and Male-Transmitted Mitochondrial Genome of Meretrix lamarckii
Source: PLoS One. 2016 Apr 15;11(4):e0153631. doi: 10.1371/journal.pone.0153631 (PMC4833323; doi:10.1371/journal.pone.0153631)
Supplement: S3 Fig — All aminoacids are reported with their one-letter code; anticodons are highlighted in yellow. (PDF) [file pone.0153631.s003.pdf]

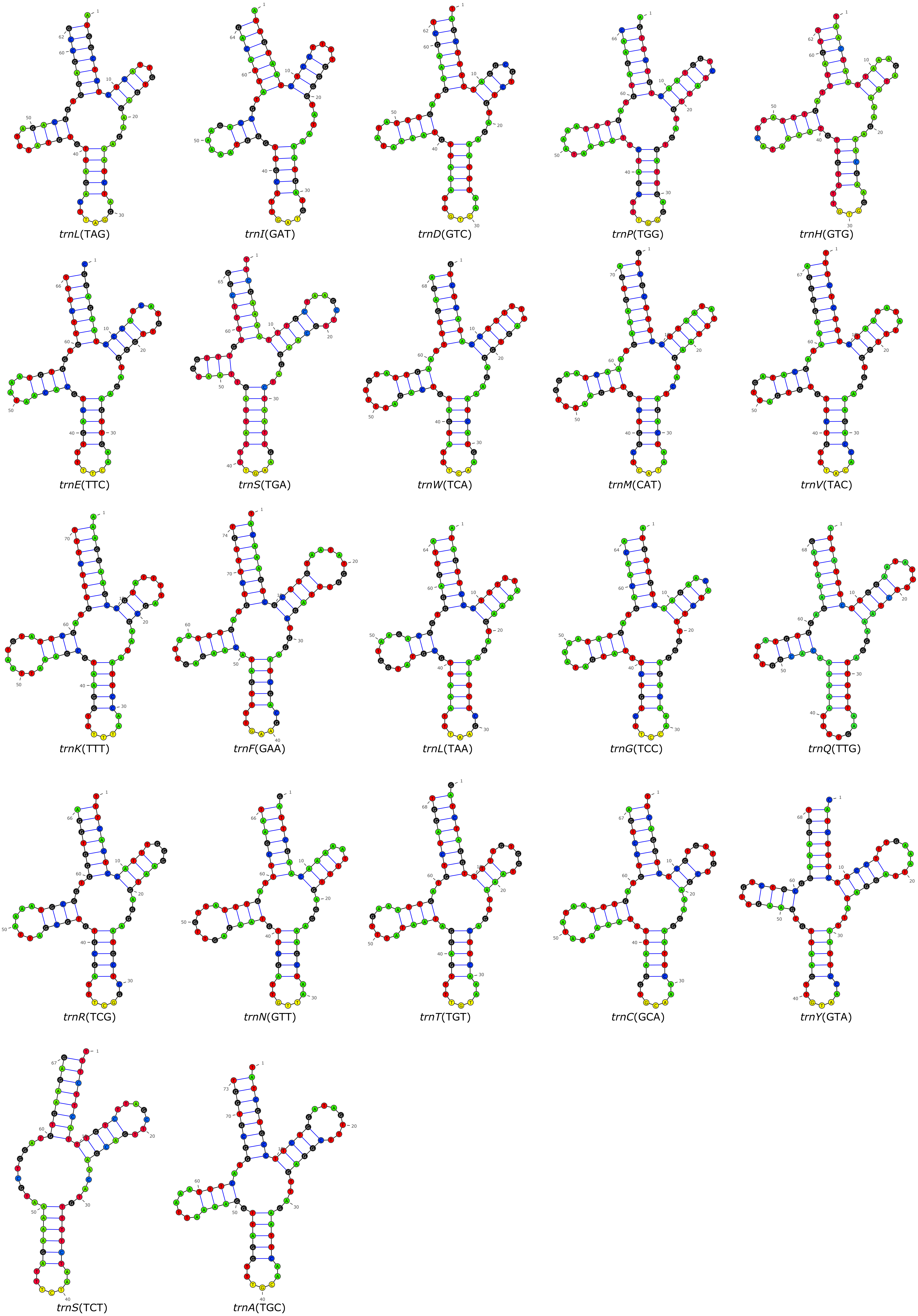
trnL(TAG)
trnI(GAT)
trnD(GTC)
trnP(TGG)
trnH(GTG)
trnE(TTC)
trnS(TGA)
trnW(TCA)
trnM(CAT)
trnV(TAC)
trnK(TTT)
trnF(GAA)
trnL(TAA)
trnG(TCC)
trnQ(TTG)
trnR(TCG)
trnN(GTT)
trnT(TGT)
trnC(GCA)
trnY(GTA)
trnS(TCT)
trnA(TGC)
