# Supplementary material for: The Complete Female- and Male-Transmitted Mitochondrial Genome of Meretrix lamarckii
Source: PLoS One. 2016 Apr 15;11(4):e0153631. doi: 10.1371/journal.pone.0153631 (PMC4833323; doi:10.1371/journal.pone.0153631)
Supplement: S4 Fig — All aminoacids are reported with their one-letter code; anticodons are highlighted in yellow. (PDF) [file pone.0153631.s004.pdf]

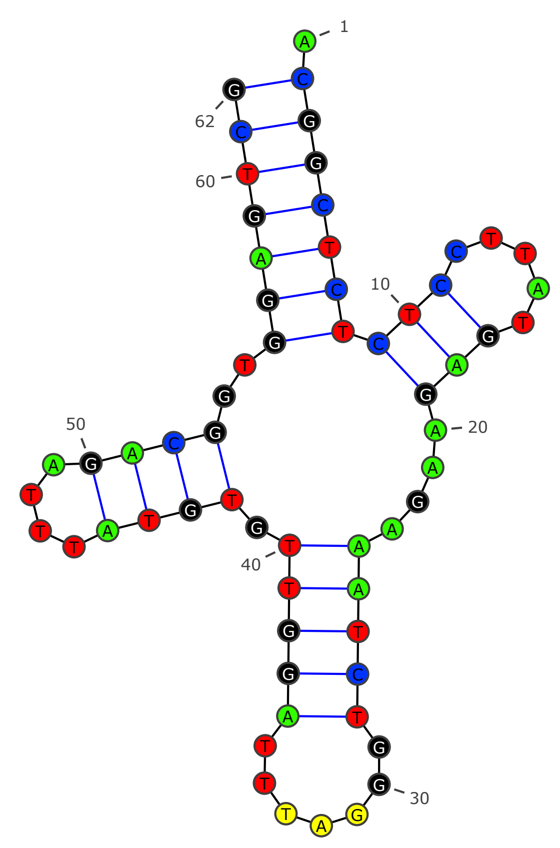

trnL(TAG)

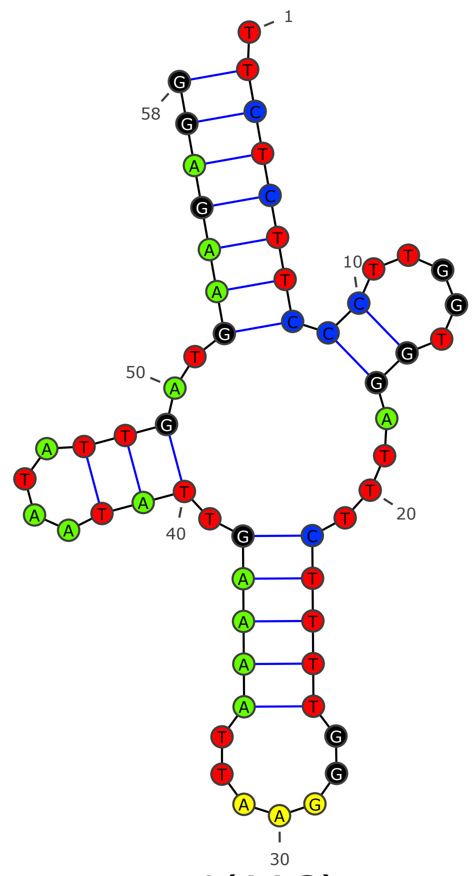

trnL(AAG)

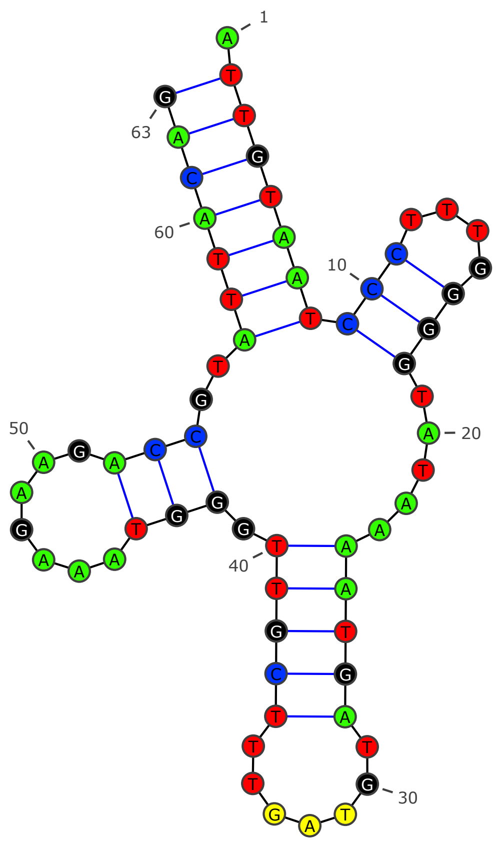

trnI(GAT)

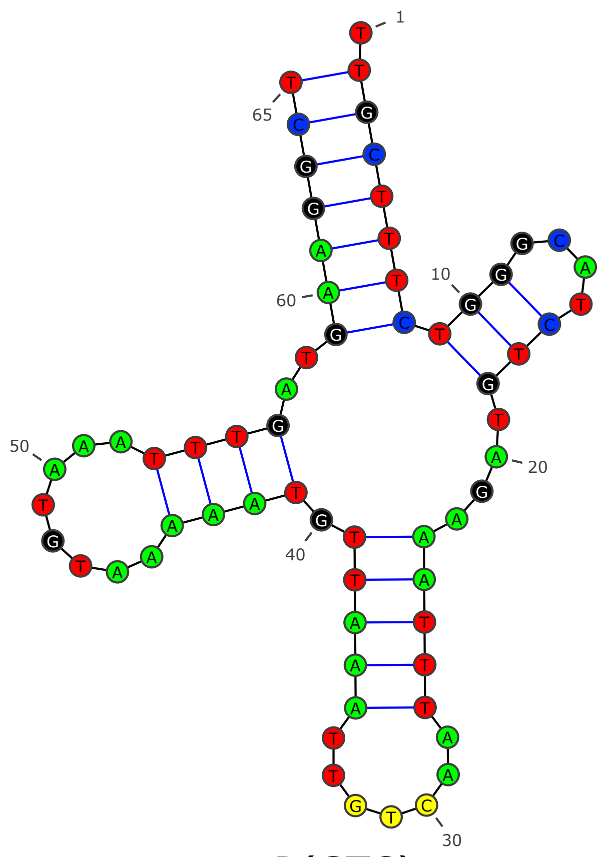

trnD(GTC)

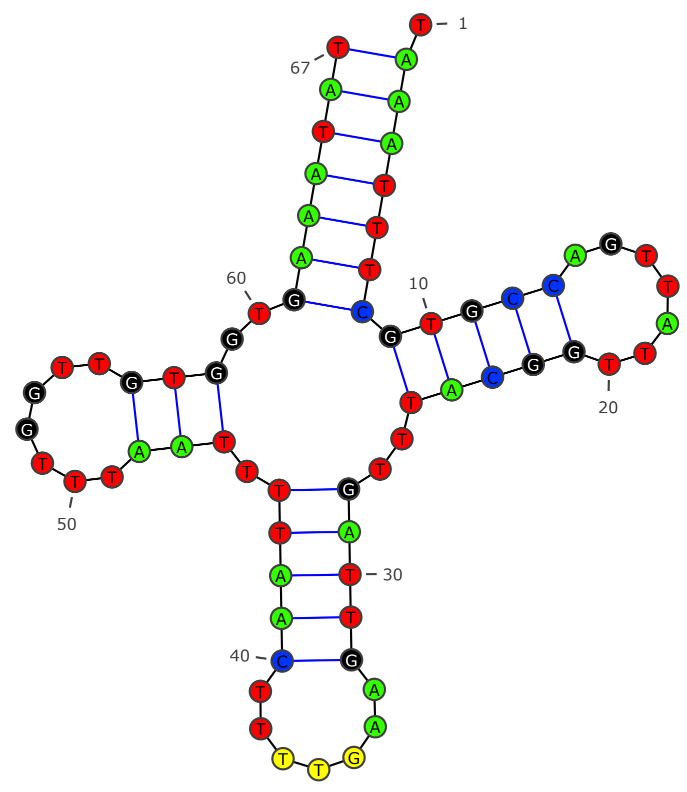

trnQ(TTG)

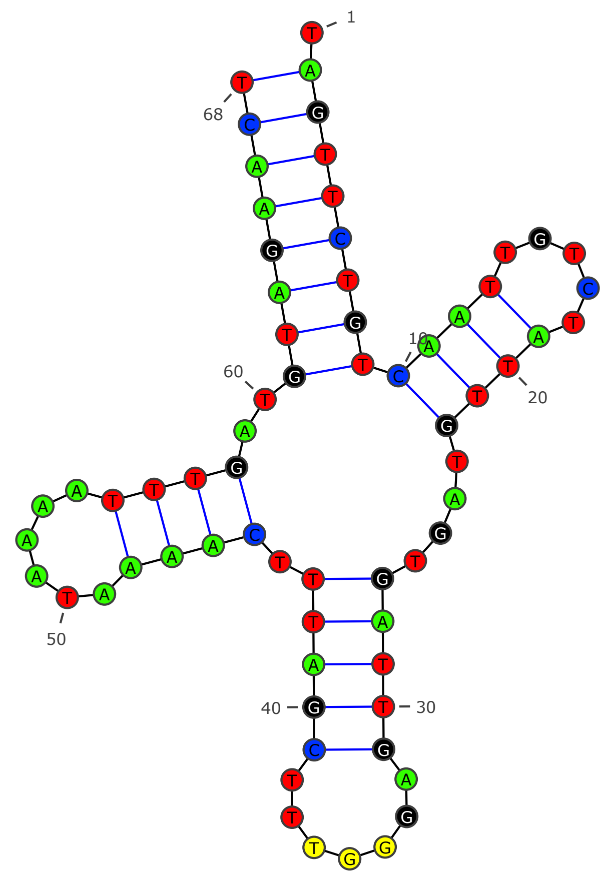

trnP(TGG)

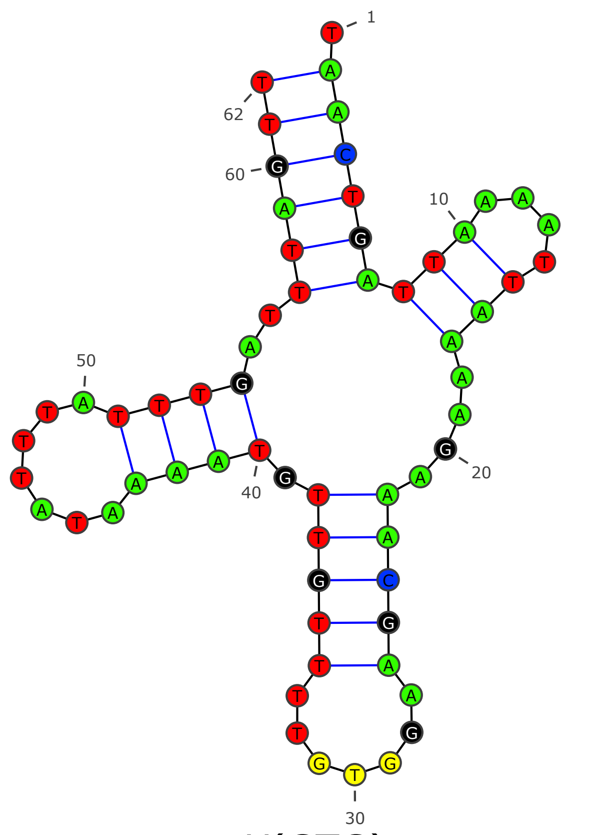

trnH(GTG)

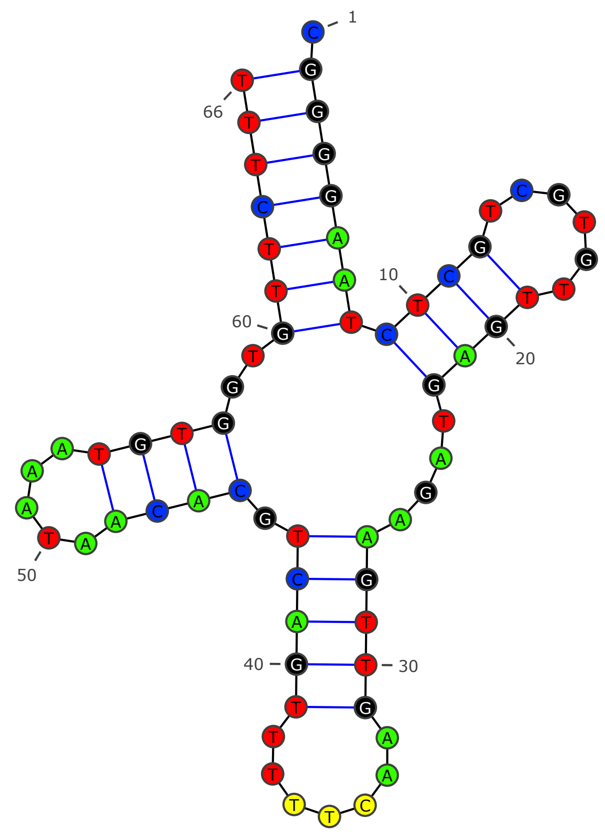

trnE(TTC)

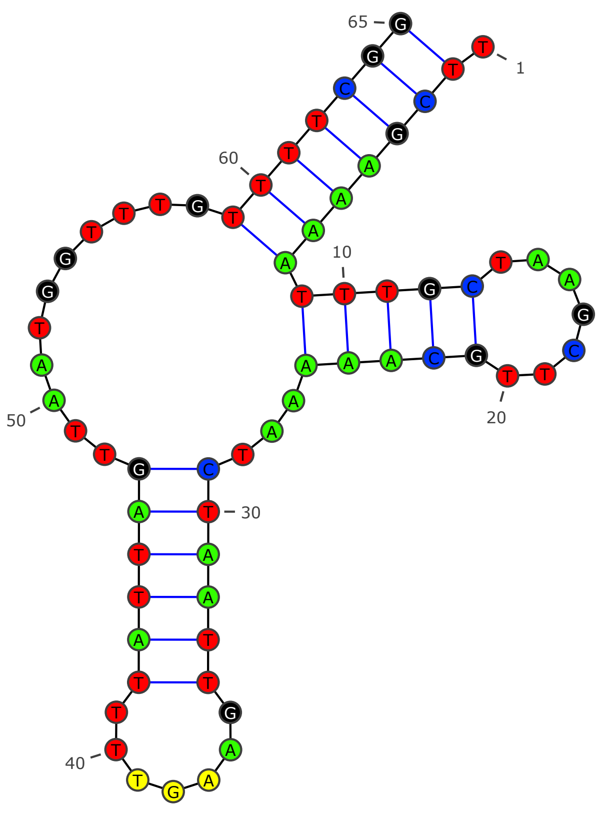

trnS(TGA)

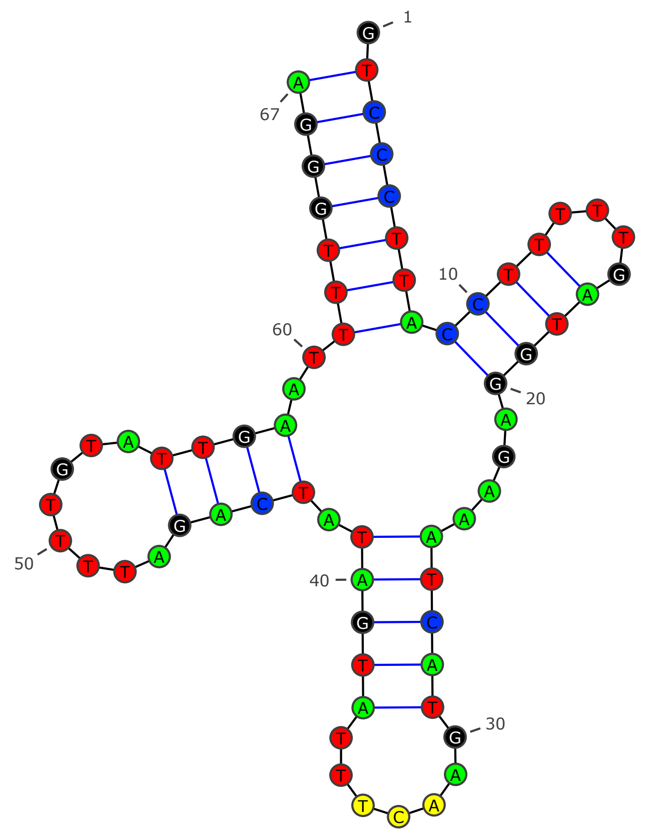

trnW(TCA)

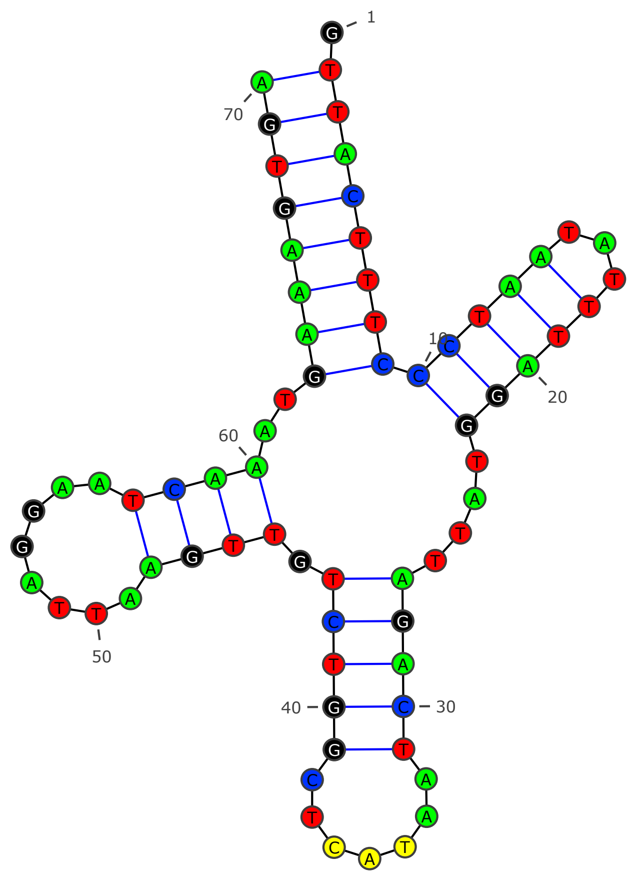

trnM(CAT)

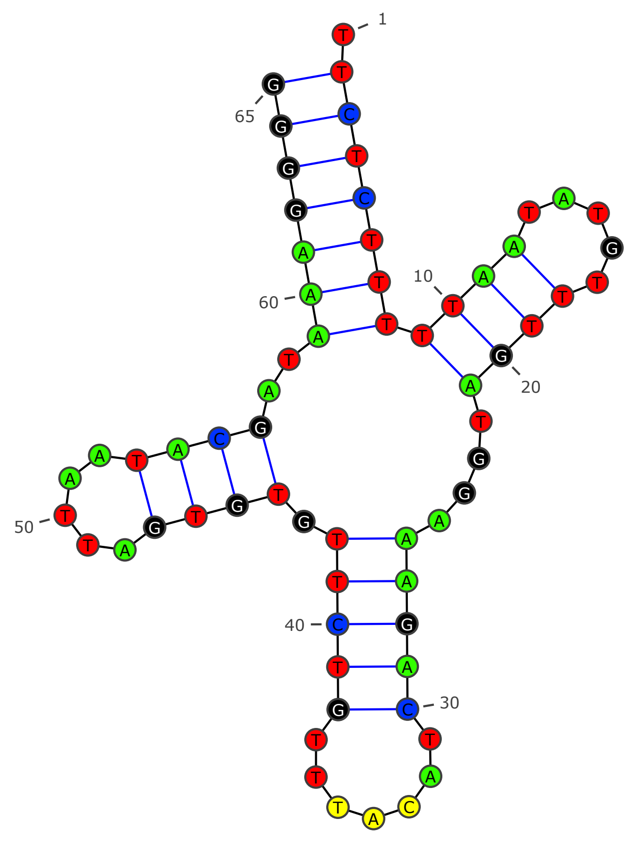

trnV(TAC)

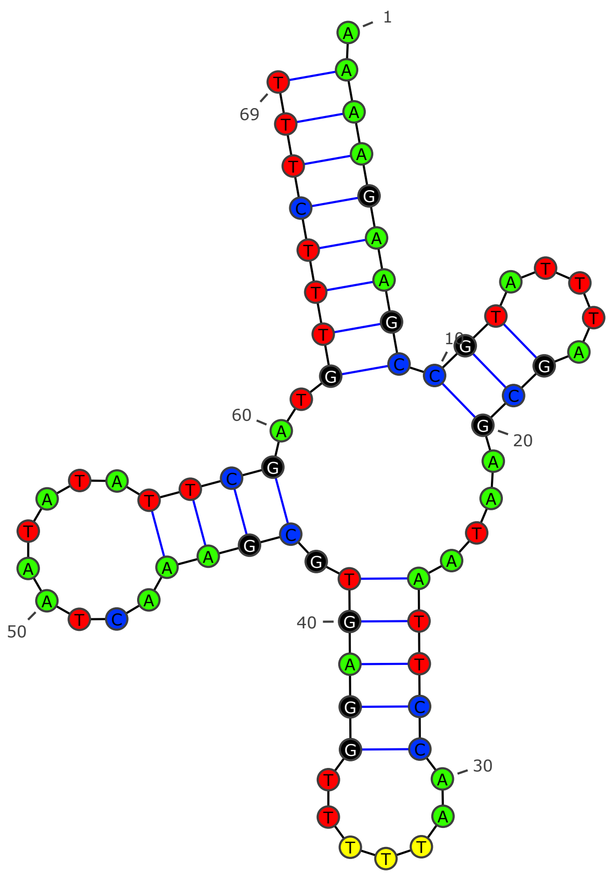

trnK(TTT)

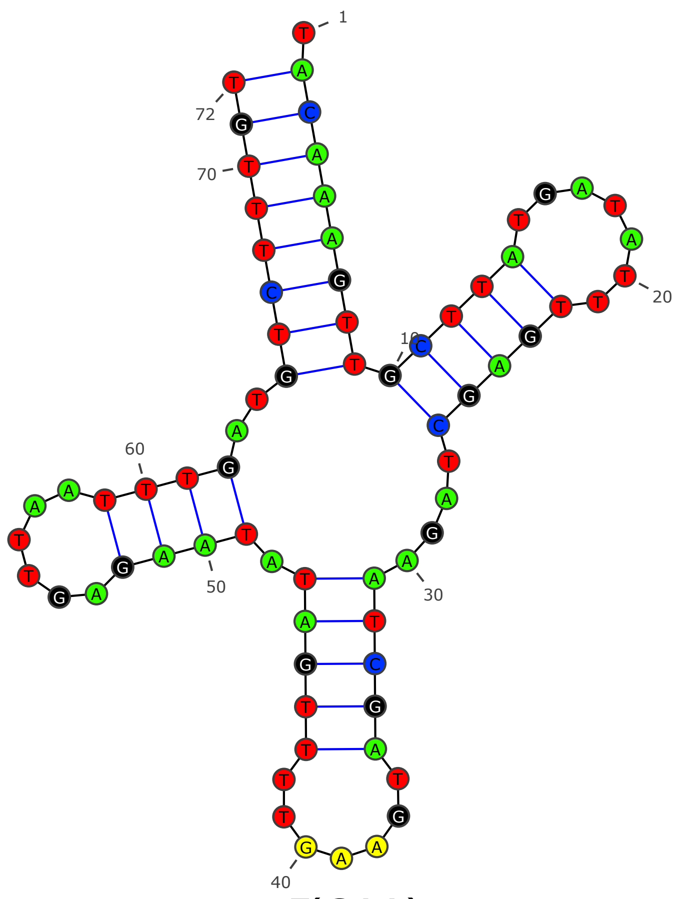

trnF(GAA)

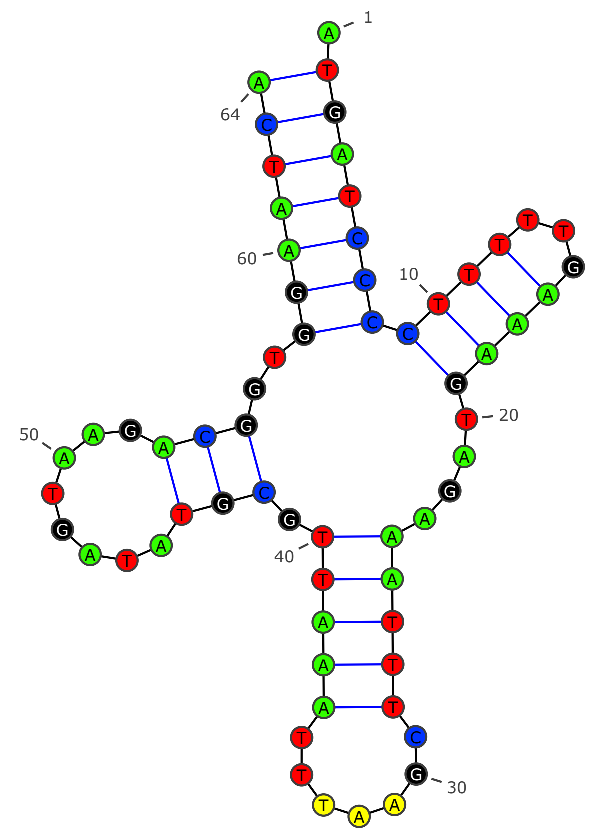

trnL(TAA)

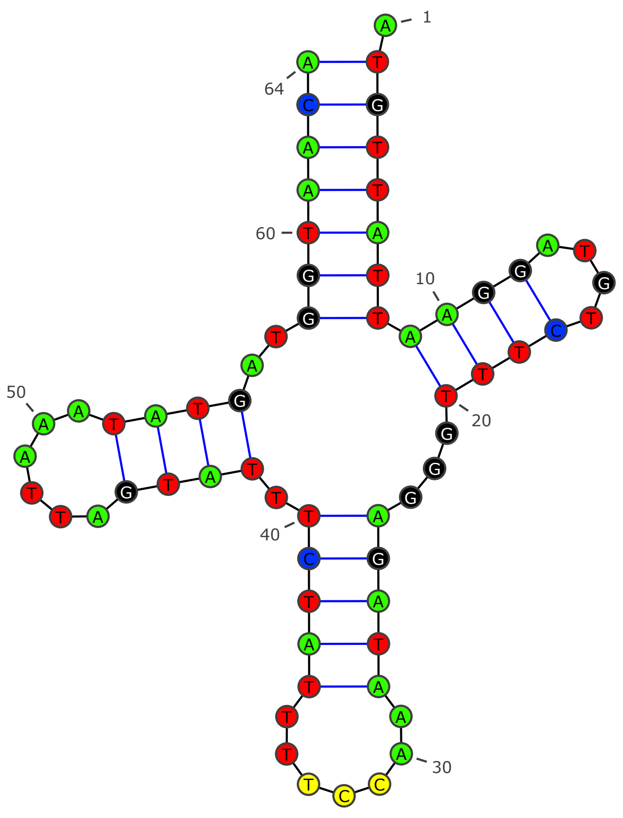

trnG(TCC)

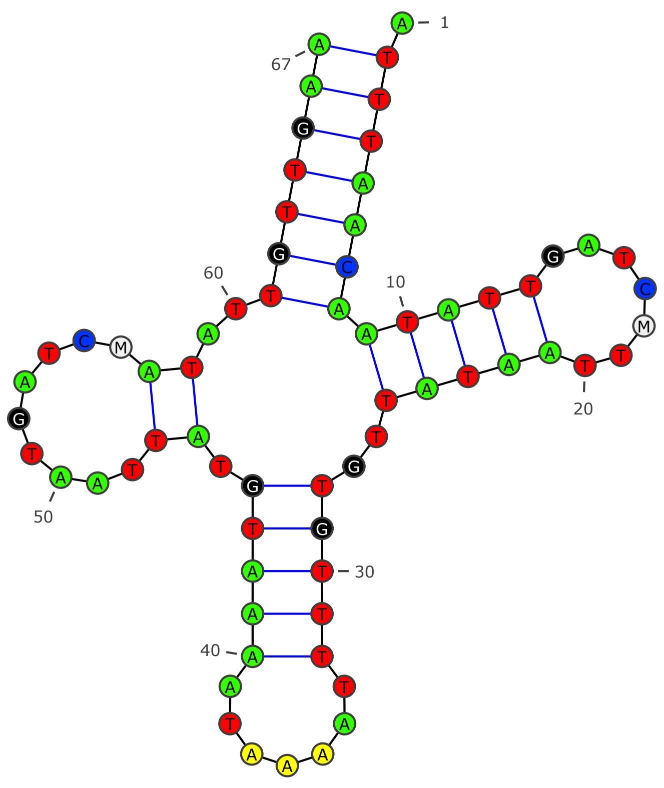

trnF(AAA)

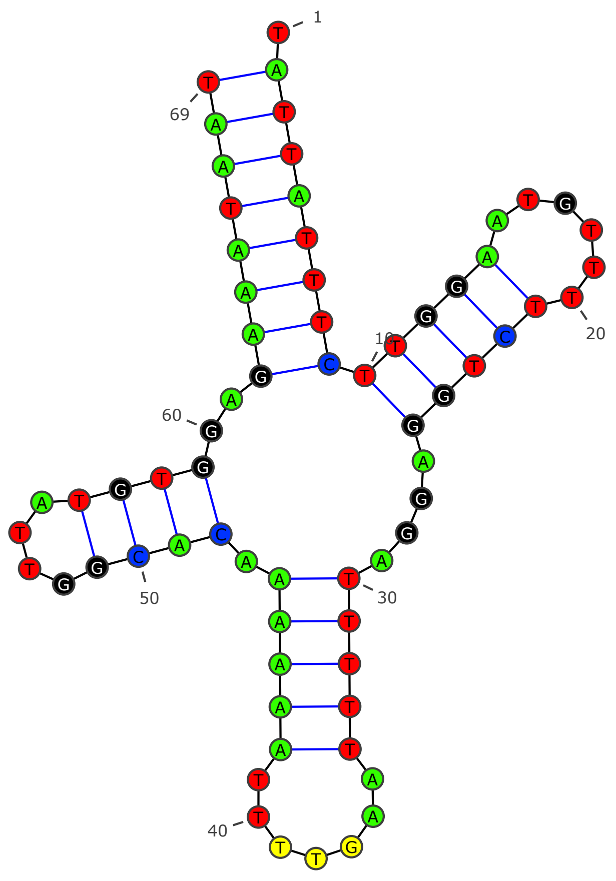

trnQ(TTG)

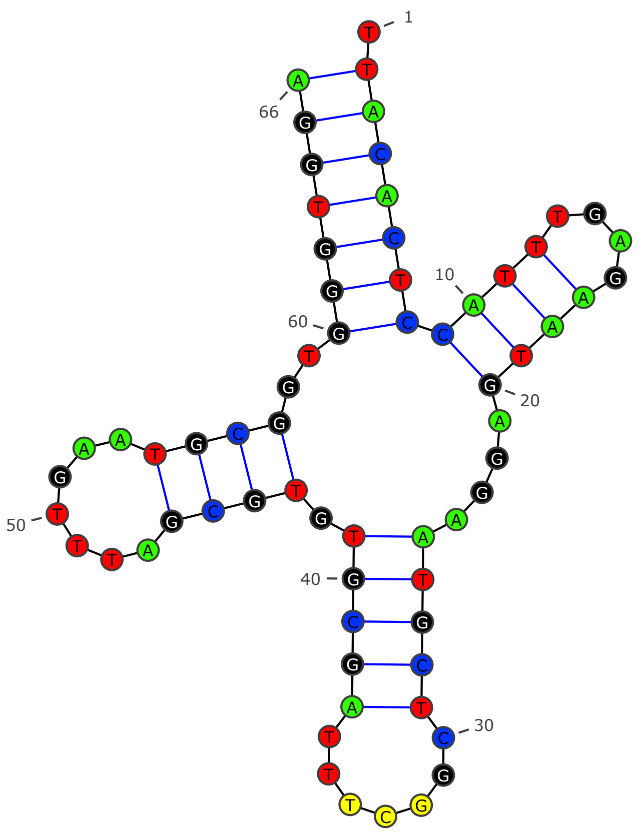

trnR(TCG)

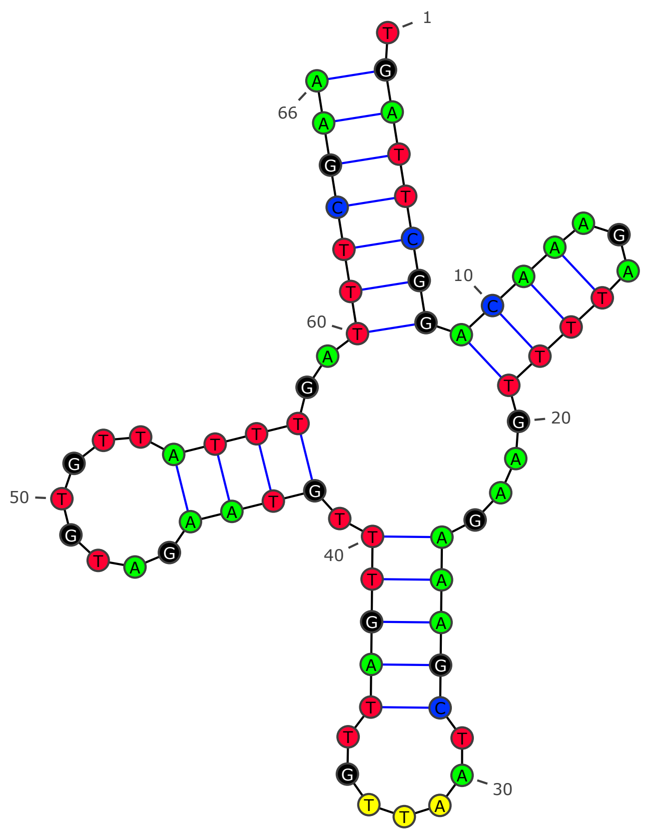

trnN(GTT)

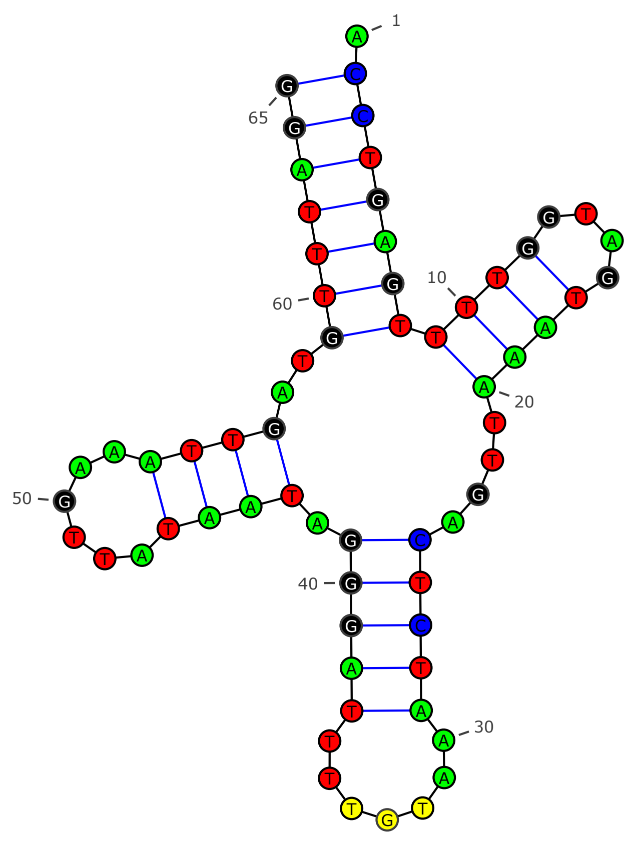

trnT(TGT)

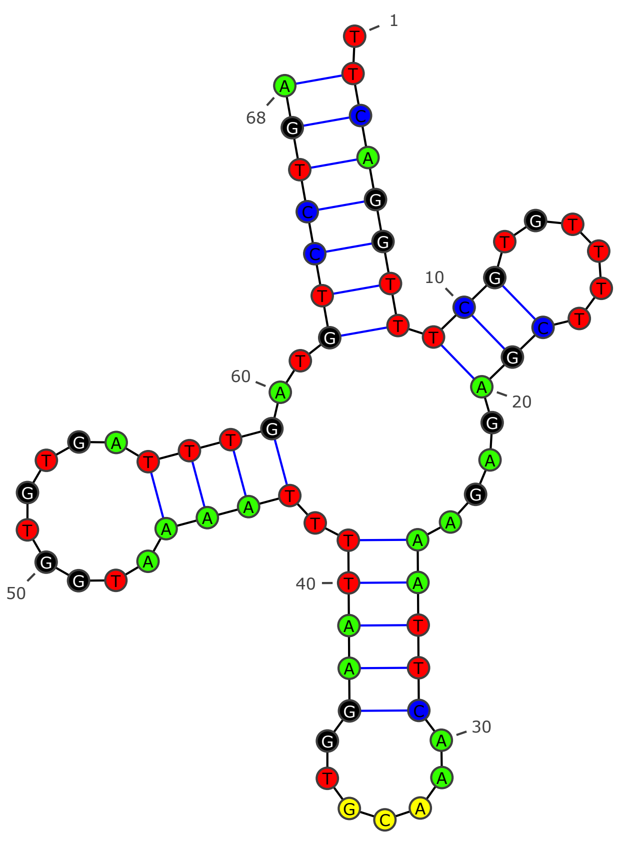

trnC(GCA)

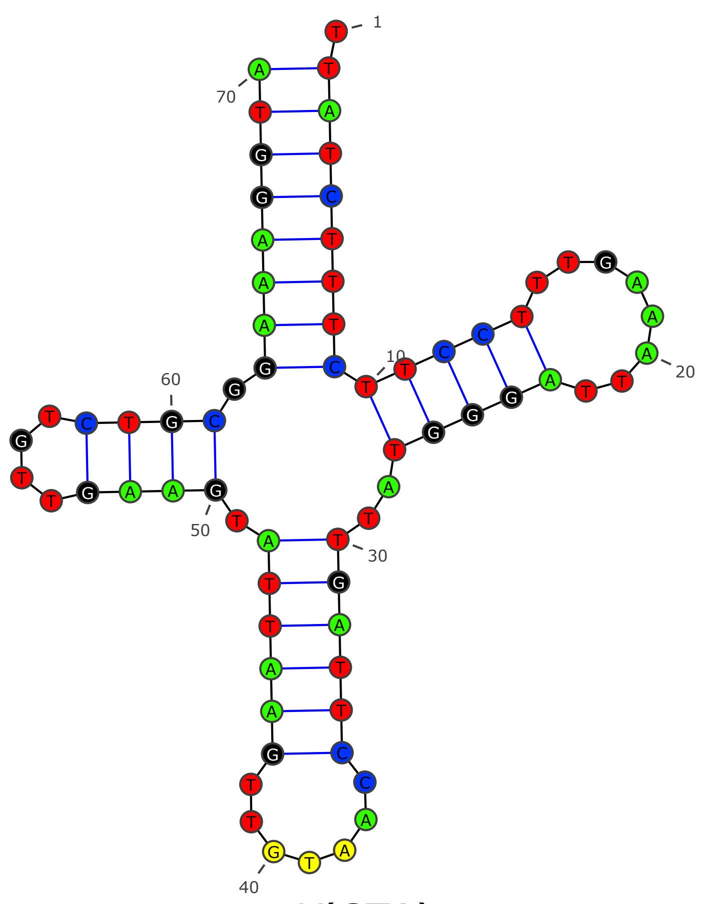

trnY(GTA)

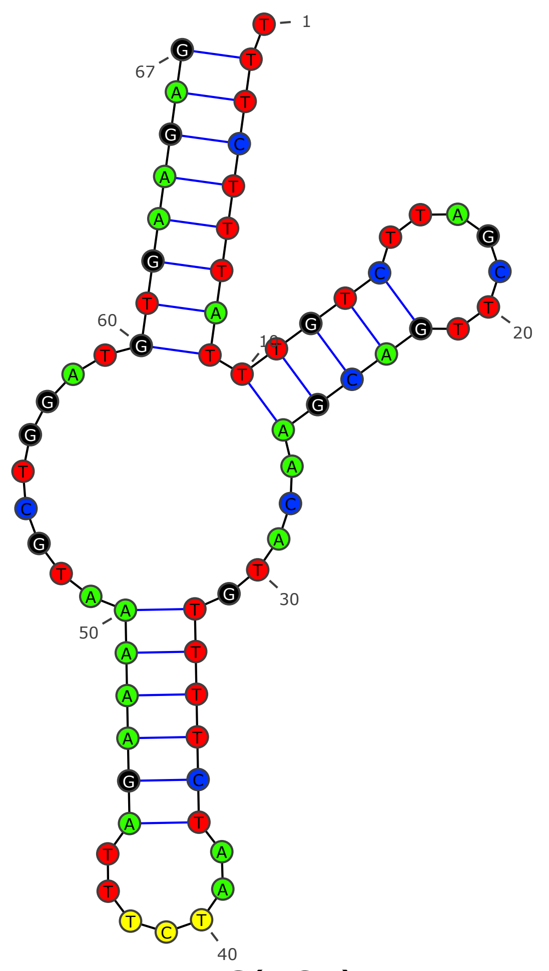

trnS(TCT)

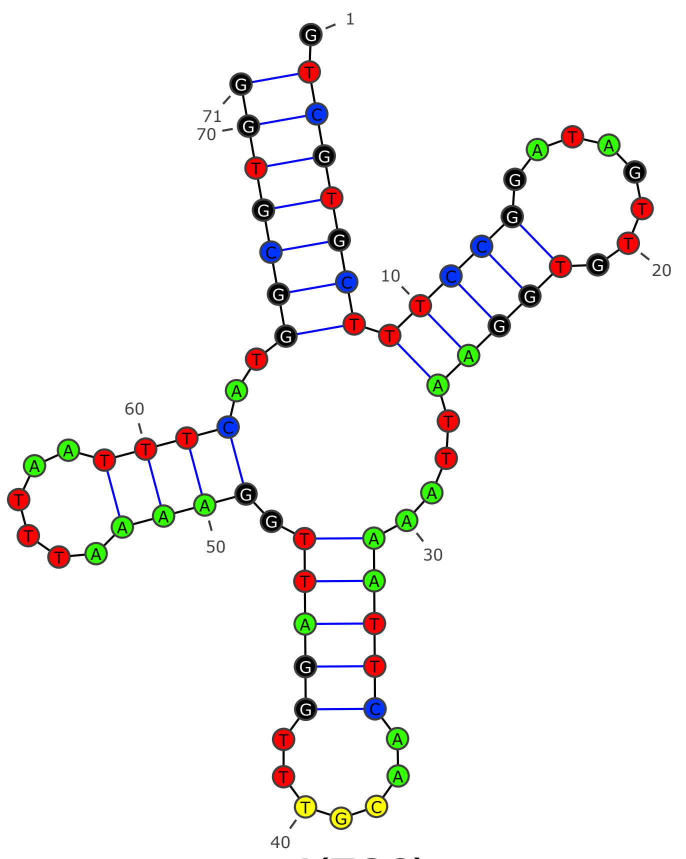

trnA(TGC)
