# Supplementary material for: The Complete Female- and Male-Transmitted Mitochondrial Genome of Meretrix lamarckii
Source: PLoS One. 2016 Apr 15;11(4):e0153631. doi: 10.1371/journal.pone.0153631 (PMC4833323; doi:10.1371/journal.pone.0153631)
Supplement: S5 Fig — The M anticodons are highlighted, while stretches of nucleotides involved in tRNA stem-loop structures are underlined. Only the relevant part of the corresponding F-UR is shown. (PDF) [file pone.0153631.s005.pdf]

```
           ***** **** ** **   *********** ********** * ** ** *********
UR2_F      GGAGAGGTAGTTTTTAGGGTTGAAAATTAAAGGTTTTCTTTGGATGATTTCCTTCTCTT
trnL_M     GGAGAAGTAG-TTATAATATTGAAAATTAAGGGTTTTCTTTAGGTGGTTCCCTTCTCTT

           *  ******* ******* ************* ******* *********  ** * ************
UR6_F      T--AAAGTGGCGTTGGTTGAATTTTAACTTTTAAAGTTAGCTTACGGTTAGGTTAATCGTGCTTTAAAT
trnQ_M     TATAAAGTGGTGTTGGTTTAATTTTAACTTTTGAAGTTAGTTTACGGTTA--TTGACCGTGCTTTAAAT

              ******** **********   ** * *****  ** *  * ***** *************
F-LUR      G--TTGTTATAACTAGTAATTAGAAAAGAGTAAAACATTTTTATCTAATTACTAGTTATAACAAC---
trnF_M     AAGTTGTTATAMCTAGTAATTATGTAA-AATAAAATTTTGTGTTATAATTMCTAGTTATAACAATTTA
```
