# Supplementary material for: The Complete Female- and Male-Transmitted Mitochondrial Genome of Meretrix lamarckii
Source: PLoS One. 2016 Apr 15;11(4):e0153631. doi: 10.1371/journal.pone.0153631 (PMC4833323; doi:10.1371/journal.pone.0153631)
Supplement: S7 Fig — Numbers refer to the positions on the mitochondrial genomes. The TAS element was taken from [66] and located on the revised Cambridge Reference Sequence (GenBank Accession Number NC_012920). (PDF) [file pone.0153631.s007.pdf]

```
                      ***********
MeLaF_LUR [16340] TGTTAAAAACCCAAC [16354]
Human_TAS [16158] ACATAAAAACCCAAT [16172]
```
