# Supplementary material for: The Complete Female- and Male-Transmitted Mitochondrial Genome of Meretrix lamarckii
Source: PLoS One. 2016 Apr 15;11(4):e0153631. doi: 10.1371/journal.pone.0153631 (PMC4833323; doi:10.1371/journal.pone.0153631)
Supplement: S8 Fig — Autocorrelograms for nucleotide trends shown in Fig 3: the autocorrelation function (acf) is plotted for lags from 0 to 17. Page 1, female mitochondrial genome; page 2, male mitochondrial genome; dashed lines, large-lag 95% standard errors. (PDF) [file pone.0153631.s008.pdf]

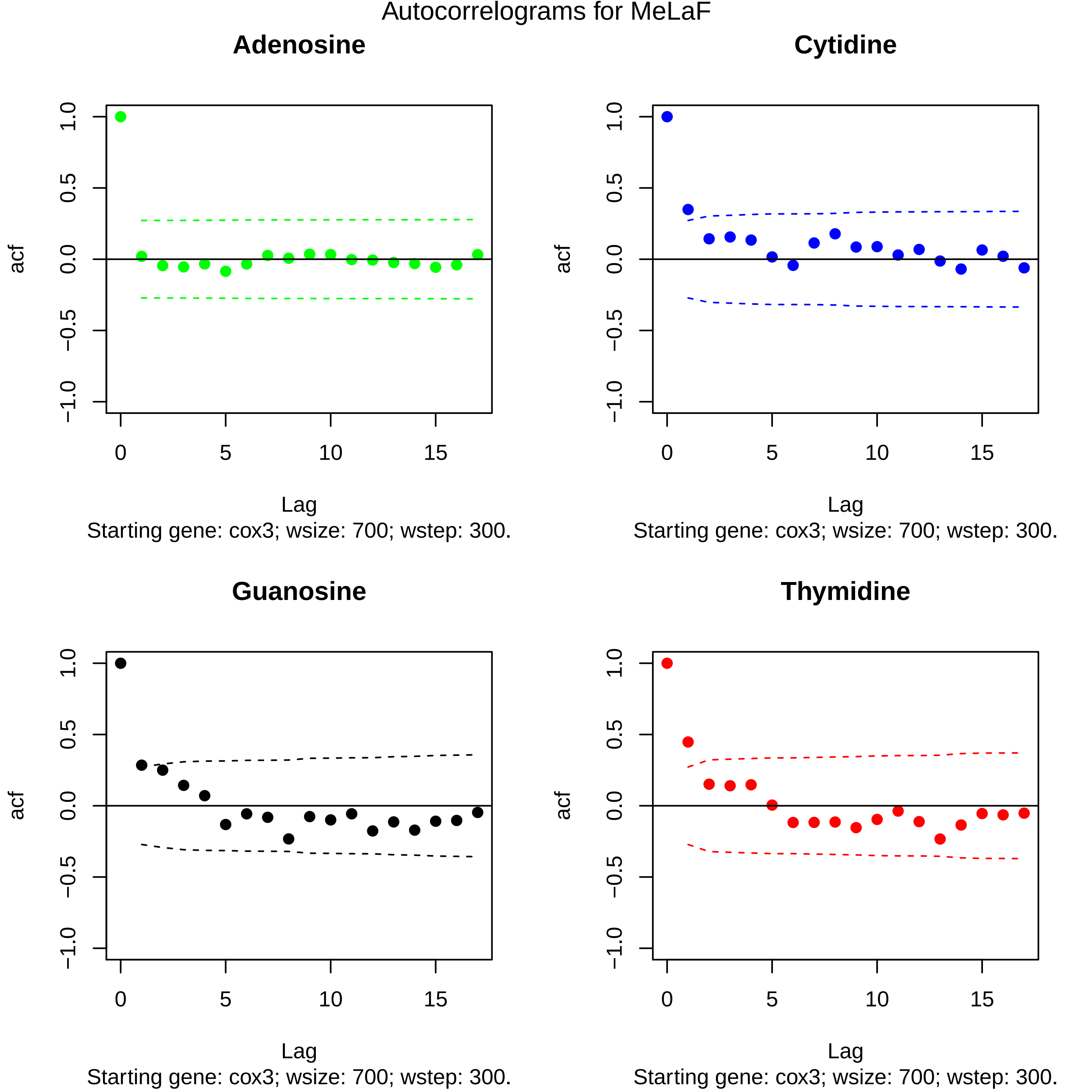
Autocorrelograms for MeLaF
Adenosine
Cytidine
Guanosine
Thymidine
acf
Lag
1.0
0.5
0.0
−0.5
−1.0
0
5
10
15
Starting gene: cox3; wsize: 700; wstep: 300.

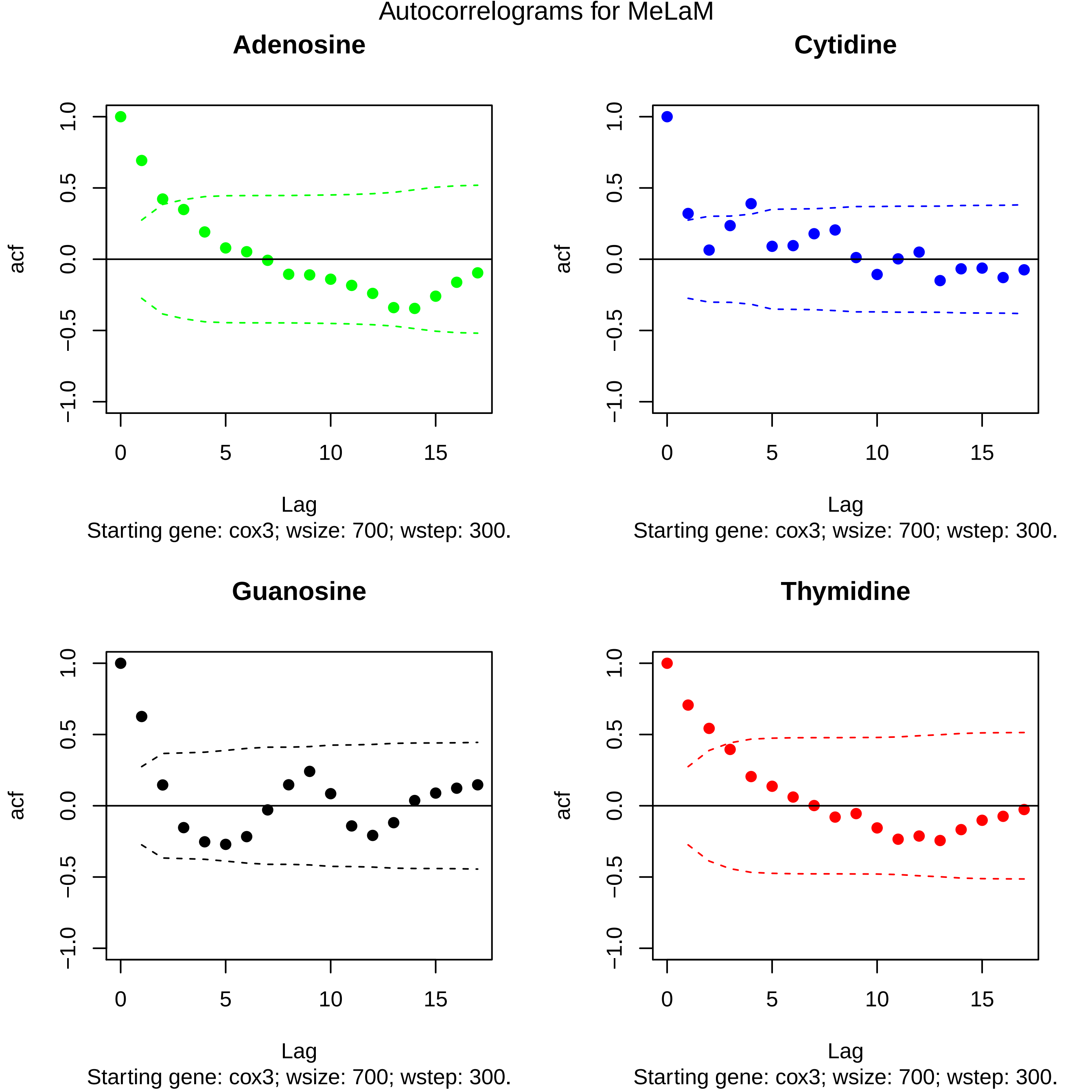
Autocorrelograms for MeLaM
Adenosine
Cytidine
Guanosine
Thymidine
acf
Lag
1.0
0.5
0.0
−0.5
−1.0
0
5
10
15
Starting gene: cox3; wsize: 700; wstep: 300.
