# Supplementary material for: The Complete Female- and Male-Transmitted Mitochondrial Genome of Meretrix lamarckii
Source: PLoS One. 2016 Apr 15;11(4):e0153631. doi: 10.1371/journal.pone.0153631 (PMC4833323; doi:10.1371/journal.pone.0153631)
Supplement: S9 Fig — Alignment between the unassigned ORF found in the LUR of the female mitochondrial genome (MeLaF) and the corresponding region of the published Meretrix lamarckii mitochondrial genome (MeLaNC_016174); numbers refer to positions on the GenBank sequences. Regions of mutations pseudogenizing the putative lost ORF are shaded in black in the published sequence; asterisks mark identical nucleotides. (PDF) [file pone.0153631.s009.pdf]

```
                           ******  *  *********  *************  ***********  ***  ************  ***************
MeLaF           [15487]  CTATTTAAAATTTTTAAAATCAAACAATTTGAGACATAGTGGGGGG-CTAAAGCTTTTGTAAACAATTCTTTTATAGAGG [15564]
MeLaNC_016174   [15242]  CTATTTTGAGCTTTTAAAACTAAACAATTTGAGATATAGTGGGGGGCTATAGCTTTTGTAAATAATTCTTTTATAGAGG [15321]

                           ** ******* ***   ************* *   ********* *************** *
MeLaF           [15565]  GAGAAAGGTGGCGTGAGAAGGTAAAATTTTTTACGTTTTTGGTAGTGTGTTAATTTTGGTAT--------- [15627]
MeLaNC_016174   [15322]  GAAAAAGGTGACGTAGAAAGGTAAAATTTTCTGTGTTTTTGGTGGTGTGTTAATTTTGGTGTTTAACACAA [15392]
```
