# Supplementary material for: The Complete Female- and Male-Transmitted Mitochondrial Genome of Meretrix lamarckii
Source: PLoS One. 2016 Apr 15;11(4):e0153631. doi: 10.1371/journal.pone.0153631 (PMC4833323; doi:10.1371/journal.pone.0153631)
Supplement: S10 Fig — MUSCLE alignment between the 109-bp repeated motif of the female LUR (MeLaF) and the 100-bp repeated motif of the published LUR (MeLaFNC_016174). Asterisks mark identical nucleotides. (PDF) [file pone.0153631.s010.pdf]

```
              *                    ********       * **** ***    * *  *   ** *  ********
MeLaF         GGGGGGTGGGGGTGTGTTGCAGTTAGGGTATGGAAGTAAAAAATTAAAACTGTAAGTGTTTTATAGTAAATATGT
MeLaNC_016174 G--------------------GTTAGGGT------TTTAAAACTTAGTTTTATGGGCAGTTAAAGGTAAATATAC

              ************* *** ** ****** * * ***  **
MeLaF         TTTTAGTTTAGTGGGTTCGGCAGTAAA-ATAGAGGCTGG------------
MeLaNC_016174 TTTTAGTTTAGTGAGTTAGGTAGTAAATAGAAAGGGAGGAATGCTGTTAGG
```
