# Supplementary material for: The Complete Female- and Male-Transmitted Mitochondrial Genome of Meretrix lamarckii
Source: PLoS One. 2016 Apr 15;11(4):e0153631. doi: 10.1371/journal.pone.0153631 (PMC4833323; doi:10.1371/journal.pone.0153631)
Supplement: S1 Script — The script is called 4F; example files and a tutorial are also provided. The same script can be downloaded at the GitHub repository https://github.com/mozoo/4F.git. (GZ) [file pone.0153631.s012.gz › tutorial_v1.0.pdf]

4F

Federico Plazzi

January 15, 2016

Chapter 1

License

Copyright © 2015 Federico Plazzi

This program is free software: you can redistribute it and/or modify it under the terms of the GNU General Public License as published by the Free Software Foundation, either version 3 of the License, or (at your option) any later version.

This program is distributed in the hope that it will be useful, but WITHOUT ANY WARRANTY; without even the implied warranty of MERCHANTABILITY or FITNESS FOR A PARTICULAR PURPOSE. See the GNU General Public License for more details.

You should have received a copy of the GNU General Public License along with this program. If not, see <`http://www.gnu.org/licenses/`>.

Chapter 2

Introduction

2.1 Background: the Theory of Everything

4F is a simple R script to perform sliding window-based analyses on four-fold degenerate codons of a mitochondrial genome. Basically, two statistics are computed: nucleotide composition and A-T content.
The nucleotide composition at fourfold degenerate sites is related with single-strand state duration during mitochondrial DNA (mtDNA) replication. As thoroughly detailed and referenced in [1, 2, 3, 4, 5], the more the heavy (H) strand remains unpaired, the more the spontaneous hydrolytic deamination of C to U and A to hX (hypoxanthine) takes place. Such an increase of T and hX in the H strand leads to a corresponding increase in the percentages of A and C in the complementary lagging (L) strand where the H strand remains for longer time in the single-stranded condition, i.e. near to the Origin of Replication (OR). Moreover, single-stranded-guanine may spontaneously oxidize to 8-hydroxyguanine, which basepairs with adenine: thus, in this case, G decreases and T increases on the H strand. In a nutshell, T will only tend to accumulate near to the OR of the H strand, while the opposite is true for A and C; finally, G may behave in either way [1, 4]. This asymmetrical composition can leave a neutral signature in fourfold degenerate sites, being them under no or weak selection.
The A-T skew at four-fold degenerate sites is known to be correlated with the position of the ORs as well: extreme (i.e., closer to ± 1) values are associated with Protein Coding Genes (PCGs) located near to the OR of the H strand, while balanced (i.e., closer to 0) values are associated with PCGs located near to the OR of the L strand [1, 3, 6].

2.2 What is 4F for

4F is an R script that scans mitochondrial genomes for neutral sites, i.e. fourfold ("4F") degenerate sites. The nucleotide composition and the A-T skew are computed on these sites over a customizable sliding window. Trends in nucleotide composition are detected using linear interpolation models. Two main outputs are produced for each analyzed genome: the nucleotide composition/A-T skew plot and a plain text tabular file with statistics related to linear models.

2.3 What is 4F not for

4F is conceived in order to locate the OR of invertebrate mitochondrial genomes: as a consequence, two strong assumptions are made over the data:

- that the mtDNA is complete and circular;
- that the genetic code is the standard invertebrate mitochondrial code (click here for the relative NCBI code table).

If this is not your case, you should browse the code to set it up for your own needs.

2.4 Autocorrelation: the Phantom Menace

The use of linear regression models to test for nucleotide trends would require independent observations, which is actually not the case when overlapping sliding windows are used. Just consider a sliding window spanning over a given region of the genome. A high score in %A (for example) will influence the possibility that the same statistics computed over the following sliding window will also be high, because of nucleotides which are shared between the two regions. The correlation between members of a series of numbers is generally called *autocorrelation*.
A simple way to detect autocorrelation in data is the *lagged scatterplot*: the series of numbers is plotted against itself with an offset k. Let the series of numbers of length N be $x_i, \quad i = 1, \ldots, N$. The lagged scatterplot for lag k is the plot of the last $N - k$ observations against the first $N - k$ ones. For example, for lag 1, observations $x_2, x_3, \ldots, x_{N-1}, x_N$ are plotted against observations $x_1, x_2, \ldots, x_{N-2}, x_{N-1}$.
A significant trend in the lagged scatterplot is an evidence of autocorrelation: namely, a positive trend indicates positive autocorrelation, i.e. a deviation from the mean of an observation influences the following one in the same direction.
The correlation coefficient r_k of the lagged scatterplot for lag k summarizes the strength of the linear relationship between the numbers of the series. It is possible to directly compute r_k using a fonction known as the *autocorrelation function* ("*acf*"). The plot of acf for different lags is called the *correlogram*.
In order to estimate the degree of autocorrelation in data, 4F will produce a

correlogram for each genome and for each nucleotide, using the large-lag standard error to compute confidence intervals [7]. A significant r_k will be shown as a point (typically) above the confidence interval. Generally speaking, the autocorrelation level is a function of the overlap between sliding windows: the larger the window size and the smaller the window step, the higher the degree of autocorrelation.
It is common that autocorrelation is significant for the first w/s lags, where w is the window size and s is the window step, because this formula estimates the number of sliding windows that span over a given site. In case of a high degree of autocorrelation in data, the standard linear interpolation may be misleading, as it relies on the independence of observations. Generally, it is possible to reduce autocorrelation levels by fine-tuning sliding window features: namely, by decreasing window size and increasing window step.
Normally, it is possible to reduce autocorrelation at least to lag 1. If it still holds, first order autocorrelation method can be solved iteratively using the Cochrane-Orcutt method [8]; for this reason, 4F exploits the `cochrane.orcutt()` function from the package `orcutt` to correct the significance of correlation and to compute the population first-order autocorrelation coefficient ρ.

2.5 Citing 4F

If you include 4F analyses in your publication, please cite [9]:

Bettinazzi S, Plazzi F, Passamonti M. The Complete Female- and Male-Transmitted Mitochondrial Genome of *Meretrix lamarckii*: a New Step towards the Characterization of Doubly Uniparental Inheritance in Venerid Bivalves. *Submitted to PLoS One.*

As 4F is an R script, you should also cite R itself [10].

2.6 Who wrote 4F

4F was written by Federico Plazzi at the Department of Biological, Geological and Environmental Sciences of the University of Bologna. Please report any bug to

`federico [dot] plazzi [at] unibo [dot] it`

Chapter 3

Running 4F

3.1 Requirements

4F is an R script. To download and install R [10], please refer to the R project site. The additional package `orcutt` is required in order to use the function `cochrane.orcutt()`. To install it, just type

```
install.packages("orcutt")
```

at the R prompt and follow on-screen instructions. You may need root privileges.

3.2 Call the script

It is suggested to call the script from within R with the function `source()`.
It is also possible to call it directly from a shell using the command `Rscript`. The script should work fine: it will probably complain about graphical output, though saving it to the working directory as `Rplots.pdf`. Conversely, you will regularly get the result file with linear regression statistics.
The script must be run from a child directory, typically called `R`, where R will save the history: this means that the script will look into the parent directory for all the necessary files (see 3.3). Main output files will also be produced there.

3.3 Input files

4F needs three kinds of input file: the configuration file (see 3.4), the input file and annotation files. As said, all these files must be in the parent directory. The input file is a FASTA alignment (with any extension) with each sequence on a single line (i.e., no newlines are allowed within a single sequence). Sequence names must exactly match those listed in the configuration file (with exclusion

of the >); however, it is possible to include more files in the input file than those listed in the configuration one.
An example input file taken from [9] is included with the script.
For each genome, an annotation file must be included: this must be called `<genome>_annotation.conf` and must be in tabular format, with headers. Headers are not hard-coded, but they must be present: afterwards, each line must list a gene name, the starting base and the ending base, separated by blanks.
If the gene is encoded on the opposite strand, starting base and ending base must simply be inverted, so that the starting base will end up in the third column and the ending base will end up in the second column.
Two example annotation files taken from [9] are included with the script.

3.4 The configuration file

The configuration file must be called `4F.conf` and, again, must be placed in the parent directory with respect to that where the script is called from.
The configuration file can list many user-defined parameters to override default values: a minimal configuration file lists only the genome names (under the `###genome###` line; see 3.5.2) and ends with the `###end###` line.
An example configuration file taken from [9] is included with the script.

3.5 Parameters

Parameters can be entered in the configuration file in any order, with the following rules:

1. the `genome` parameter is mandatory;
2. all parameters are declared through a line with the parameter name between triple octothorps (`###`);
3. the parameter value must immediately follow the declaration line on the following line;
4. only the `genome` and the `genes` parameters are allowed to have multiple values, listed on multiple, consecutive lines;
5. The configuration file must close with a `###end###` line.

Specific details about available parameter are given below, with the default value between square brackets.

3.5.1 gene

`[cox1]` The `gene` parameter sets the starting gene for the nucleotide composition plot and the A-T content plot. It is possible to set a single gene to be used for

all genomes, or a different starting gene for each analyzed genome.
In the latter case, it is sufficient to list the starting genes on separate lines after the `###gene###` header, in the same order in which respective genomes are listed. Pay attention: R will complain when the number of starting genes is different from either 1 or the number of genomes!

3.5.2 genome

`[`*`no default`*`]` The `genome` parameter is followed by a list of genomes to be analyzed, each on a different line. The script will browse the input FASTA file looking for a sequence name exactly matching each element of the genome parameter: the following line will be assumed to be the complete sequence of that genome.

3.5.3 infile

`[infile]` The `infile` parameter is the name of the input file. This must be a FASTA file with mtDNAs on single lines. It will be looked for in the parent directory.

3.5.4 ncol

`[`*`no default`*`]` The main output of the script is a multi-panel picture with two plots (nucleotide composition and A-T content) for each given genome. The script will try to determine how many plots it is better to place on the same line, and therefore how many plot columns should be produced. 4F will try to determine the number of rows and columns of the output picture trying to make it as balanced as possible (i.e., trying to put as many plots on the last row as in previous rows and trying to produce a squared picture); however, it is possible to override automatically-determined values. The two plots referring to the same genome will always be placed on the same line. The `ncol` parameter specifies the user-defined number of columns in the final picture.

3.5.5 nrow

`[`*`no default`*`]` The `nrow` parameter specifies the user-defined number of columns in the final picture. See 3.5.4.

3.5.6 outfile

`[outfile]` The `outfile` parameter is the name of the outfile file, where linear regression statistics will be saved in a tabular format for each genome. It will be produced in the parent directory.

3.5.7 plotfile

`[plot.pdf]` The `plotfile` parameter is the name of the plot file, a PDF file where plots of nucleotide composition/A-T content will be saved. A single file with different plots will be produced if more than one genome is indicated. It will be produced in the parent directory.

3.5.8 wsize

`[200]` The `wsize` parameter sets the size of the sliding window. If `wsize` is even, the sliding window size will be set to `wsize` + 1 to allow exact centering on sites.

3.5.9 wstep

`[50]` The `wstep` parameter sets the distances between centers of sliding window. The first sliding window will be centered at site `wstep`.

Chapter 4

Output

Basically, two output files are produced in the parent directory.

- A tabular file with linear regression statistics for each genome for each nucleotide, starting at the specified PCG. Linear regression statistics are the intercept, the slope, the R^2, the adjusted R^2, and the p-value. The `lm()` function of R is used to produce these statistics. Moreover, the `cochrane.orcutt()` function is used to compute regression significance and ρ for lag 1, in case autocorrelation is detected (see 2.4 for more details). An example result file taken from [9] is included with the script.

- A multi-panel PDF file is produced with two plots for each genome: the nucleotide composition at four-fold degenerate sites and the A-T content, both starting at the specified gene. Each nucleotide is shown in a different colour: A, green; C, blue, G, black, T, red. Under each plot, the starting gene and the sliding window features are printed.
  An example plot taken from [9] is included with the script.

Furthermore, correlograms for each genome are produced in the current directory: they are named `<genome>_acf.pdf`. Colours are as above; again, sliding window features are printed below each plot.
Two example correlograms taken from [9] are included with the script.

Bibliography

[1] Reyes A, Gissi C, Pesole G, Saccone C. Asymmetrical directional mutation pressure in the mitochondrial genome of mammals. Mol Biol Evol. 1998;15: 957-966.

[2] Saccone C, Gissi C, Reyes A, Larizza A, Sbisà E, Pesole G. Mitochondrial DNA in metazoa: degree of freedom in a frozen event. Gene 2002;286: 3-12.

[3] Faith JJ, Pollock DD. Likelihood analysis of asymmetrical mutation bias gradients in vertebrate mitochondrial genomes. Genetics 2003;165: 735-745.

[4] Rodakis GC, Cao L, Mizi A, Kenchington EL, Zouros E. Nucleotide content gradients in maternally and paternally inherited mitochondrial genomes of the mussel Mytilus. J Mol Evol. 2007;65: 124-136.

[5] Breton S, Doucet-Beaupré H, Stewart DT, Piontkivska H, Karmakar M, Bogan AE, Blier PU, Hoeh WR. Comparative mitochondrial genomics of freshwater mussels (Bivalvia: Unionida) with Doubly Uniparental Inheritance of mtDNA: gender-specific Open Reading Frames and putative origins of replication. Genetics. 2009;183: 1575-1589.

[6] Fonseca MM, Posada D, Harris JD. Inverted replication of vertebrate mitochondria. Mol Biol Evol. 2008;25: 805-808.

[7] Anderson O. Time series analysis and forecasting: the Box-Jenkins approach. 1976; Butterworths, London.

[8] Cochrane D, Orcutt GH. Application of Least Square Regression to Relationships Containing Auto-Correlated Error Terms. J Am Stat Assoc. 1949;44: 32-61.

[9] Bettinazzi S, Plazzi F, Passamonti M. The Complete Female- and Male-Transmitted Mitochondrial Genome of *Meretrix lamarckii*: a New Step towards the Characterization of Doubly Uniparental Inheritance in Venerid Bivalves. *Submitted to PLoS One.*

[10] R Development Core Team. R: A language and environment for statistical computing. Vienna: R Foundation for Statistical Computing; 2013. Available: `http://www.R-project.org/`.
