# Supplementary material for: The Complete Female- and Male-Transmitted Mitochondrial Genome of Meretrix lamarckii
Source: PLoS One. 2016 Apr 15;11(4):e0153631. doi: 10.1371/journal.pone.0153631 (PMC4833323; doi:10.1371/journal.pone.0153631)
Supplement: S1 Script — The script is called 4F; example files and a tutorial are also provided. The same script can be downloaded at the GitHub repository https://github.com/mozoo/4F.git. (GZ) [file pone.0153631.s012.gz › MeLaM_acf.pdf]

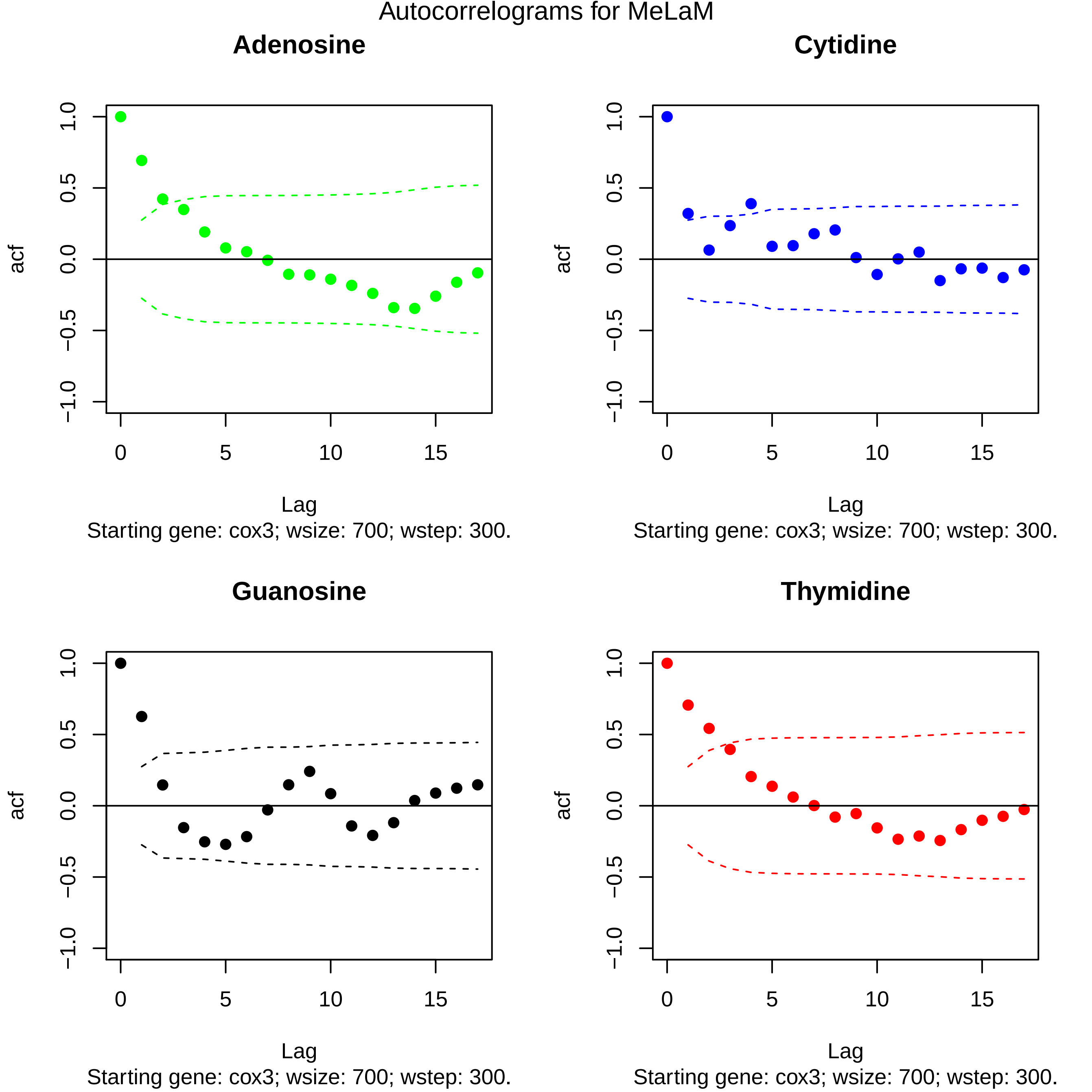
Autocorrelograms for MeLaM
Adenosine
Cytidine
Guanosine
Thymidine
acf
Lag
1.0
0.5
0.0
−0.5
−1.0
0
5
10
15
Starting gene: cox3; wsize: 700; wstep: 300.
