# Supplementary material for: The Complete Female- and Male-Transmitted Mitochondrial Genome of Meretrix lamarckii
Source: PLoS One. 2016 Apr 15;11(4):e0153631. doi: 10.1371/journal.pone.0153631 (PMC4833323; doi:10.1371/journal.pone.0153631)
Supplement: S1 Table — Primers are listed by pairs, showing for each pair the forward (F) and the reverse (R) primers in the column "Strand". In the column "Sex" it is specified if a given pair was used for the female genome (F), for the male genome (M), or for both (both). For amplicons > 2,000 bp the Herculase enzyme was used (see text for details). Where two annealing temperatures are listed, the first one refers to the female genome and the second one to the male genome. (PDF) [file pone.0153631.s013.pdf]

Primer name	Strand	Sex	Sequence 5'-3'	Annealing temperature (°C)	time	Amplicon (bp)	Reference
M-cox1-cytb-F	F	both	TGGTGCTTCTTCTATTATGTCTGGTATT	50-51.5	20''	>7,000	[18]
cox1-rnl-R2	R		TGCGTCCAGCCATTCATACTATCCCTC				[18]
rnl-nad5-F2	F	both	TTGAGGGATAGTATGAATGGCTGGACGC	53-50.5	20''	>4,500	[18]
rnl-nad5-R2	R		CCTTCTTCAAATCTACAAAGCTCAACGCC				[18]
nad5-cox1-F2	F	both	TTACGGCTTTATCTAATCGTGTTGGTGA	56-53	30''	>9,000	[18]
nad5-cox1-R	R		AGCAAAAAACCAGTCACAGCAATACACC				[18]
MeLaM_P1_424F	F	M	ACTTTGATTATTGCTATTCCTACT	54	30''	6,256	this study
MeLaM_P2-6256R	R		TAAATTCTGATATTACACACACCT				this study
MeLaM_P3_913F	F	M	AGTTTTCCTATCTTTTGGTAGTTA	54	30''	2,797	this study
MeLaM_P4_3710R	R		ATAAACGCCTATCTTACTTATTTC				this study
MeLaM_Pbuco2_F	F	M	TTTAGTATTGGTTTGTTAATTC	48	30''	< 300	this study
MeLaM_Pbuco3_R	R		ACCAAATTATTCTGATTTTT				this study
MeLaM_P5_F	F	M	ATTTTATTAAGGGTTTTGTAGTTG	50	30''	9,591	this study
MeLaM_P6_R	R		ACCAATAGAAAGAATAGCATAAAT				this study
MeLaF_P1_F	F	F	ACTATTTATCCTCCTTTATCAAAT	48	30''	8,273	this study
MeLaF_P2_R	R		CAAAACAAACTAAAGAAATAATCA				this study
MeLaF_P3_953F	F	F	GAGGTGTAAAGAGTTGAAGTTAAT	54	30''	3,175	this study
MeLaF_P4_4128R	R		TATAATAAACAACCAACAAGAAAG				this study
MeLaF_P5_794F	F	F	ATCTAAATGGTTCTCTTTTATTGA	50	30''	7,259	this study
MeLaF_P6_8053R	R		TTATTAACTATAACCCCAATATCC				this study
Primer M 1_1196	F	M	TAGTTAGAGTTTTCATAATGGTTT	54	30''	3,600	this study
Primer M 2_5210	R		ATTAACATCTCGTATAATATGCAC				this study
Primer M 3_8426	F	M	CTTATTTCTTTAGGGTTTTTAGTT	54	1'	1,021	this study
Primer M 4_9896	R		CCATAACCATAGCATTAGGTA				this study
Primer M 5 bis F	F	M	CTCCTACTCCTGTTTCTAGTT	54	30''	9,000	this study

Primer M 5 bis(A) R	R		TATAACCATACTGACCTAAAAGT				this study
Primer M 5 bis(B) R	R		TTACTACAGACCTATCACAATTC				this study
Primer F 1_855	F	F	TTAGTCTATGTAGTTTGTTTCATT	54	30"	6,000	this study
Primer F 2_7144	R		AAAGTAAATTCTCACATTATACAC				this study
Primer F 3_9925	F	F	GGTTGTTACTCGTTGATCTTA	54	1'	1,710	this study
Primer F 4_11921	R		CTAATAAATACACAAGAAGTGAAAT				this study
Primer F 5_13967	F	F	TATTTTACTGAGGATTATTCTGTT	54	30"	5,043	this study
Primer F 6_19311	R		AAATCTTTTTACGACCTACAC				this study
Primer M 1_2082	F	M	ATAAGTTGTTTTAGGTTTAGTCTG	54	30"	2,193	this study
Primer M 2_4275	R		GCACATTTTTACTATAATCTTTAAT				this study
Primer M 5_12051	F	M	GTTATGCTGGTAGAGAGTTTTA	54	30"	8,089	this study
Primer M 6_20140	R		AAAATATAAAGTACCAATATCCTT				this study
Primer F 1_1803	F	F	TTAGCTGAATATAGAAATATAATG	54	30"	4,455	this study
Primer F 2_6258	R		TAAAACAATAAACAAAGTACAAC				this study
Primer F 3_10728	F	F	GTCAGAGGTTATTAGATTTTTAG	54	1'	1,189	this study
Primer F 4_11917	R		ATAAATACACAAGAAGTGAAATC				this study
Primer F 5_14787	F	F	ATAGTATTTCTGTCTTCCAAAC	54	30"	3,662	this study
Primer F 6_18449	R		CTCTAATCCTTGTTTTTAGTGTA				this study
Primer M 1_2199	F	M	TATAGTTTGTTGTATAGGATTTGT	54	1'	1,049	this study
Primer M 2_3247	R		ACATTAAAACCAAAACTGAA				this study
Primer M 5_12618	F	M	ATTAGAGTGTAGTGTTTGAGGTAT	54	30"	6,596	this study
Primer M 6_19213	R		TAAAACGCTTAATATCTCTTTTAT				this study
Primer F 1_2428	F	F	GAGTTTTAGAATAATAAGGTTAGC	54	30"	2,879	this study
Primer F 2_5306	R		TACTTAAAATATCAATCTCCTTCT				this study
Primer F 3_11495	F	F	TTTGAAAATAAGAGTCCTTAGA	54	1'	517	this study
Primer F 4_11917	R		ATAAATACACAAGAAGTGAAATC				this study
Primer F 5_15396	F	F	ACGTAAATGAGCTGTATTCTAT	56	30"	2,032	this study

Primer F 6_17427	R		TTTTACTTCCATACCCTAACT				this study
M P5_14347	F	M	TGAGAATATAGTTTTGAAGTAGC	54	30''	4,469	this study
M P6_18815	R		TTTACACCAGTAGACAACTAACT				this study
F P1_3474	F	F	TTATTTTATGTTCTCGTCTTAGT	54	1'	883	this study
F P2_4356	R		TCTATTAAAGTCTGGAAAATGT				this study
F P3 STFPA F	F	F	TGGTAGGAAGAAAAAGTGTG	54	1'	79	this study
F P4 STFPA R	R		AAGAATAGGCCCCACTCATA				this study
M P5_15023	F	M	ATAAACCGTATTTTATGATTGT	54	30''	2,836	this study
M P6_17859	R		AACTAACTAAGCCTGTTTCTAA				this study
F P5_14931	F	F	GTAGTGTGTTAATTTTGGTATTT	54	30''	2,133	this study
F P6_17064	R		CTCTATTTTCCCTCTATTTTAC				this study
Fem ND4_T 15737 F	F	F	TAGTTTAGTTTTTGTTTTAATAGG	54	1'	392	this study
Fem ND4_T 16128 R	R		CAGCAAGTAAAGGTAAAGAA				this study
M COX2_K F	F	M	ATATTGATGACGTTGACACT	54	1'	477	this study
M COX2_K R	R		AAGGCACATTTTTACTATAATC				this study
Mela Mas P5_15719	F	M	CTCTATGTTTTCGGTAATATGT	54	1'	1,321	this study
Mela Mas P6_17039	R		CTATTAAAAATTCAACTCTAACAA				this study
Mela Fem P5_15959	F	F	TAATAAGTTAGGTCAAAGTCAGAT	54	1'	1,418	this study
Mela Fem P6_17376	R		AAAAGTACACTAAGACCAGAATAC				this study
MeLa Mas P5_15794	F	M	CTAGACATACCGTGTAATGG	54	1'	479	this study
MeLa Mas P6_16272	R		AAGCAATTTTTAGTTAGTTAGATT				this study
MeLa Fem P5_16089	F	F	TAAATTATTGGTTTCCACAA	54	1'	213	this study
MeLa Fem P6_16301	R		TAAAAACACAGCTCTAACAACTA				this study
MelaMas tRNA-Asn (gtt)_17007 F	F	M	GGAGTAAGAGTTTACCTCACAT	54	1'	<100	this study
MelaMas tRNA-Asn (gtt)_17007 R	R		GACTCAAAACCATCATTTAAC				this study
