# Supplementary material for: The Complete Female- and Male-Transmitted Mitochondrial Genome of Meretrix lamarckii
Source: PLoS One. 2016 Apr 15;11(4):e0153631. doi: 10.1371/journal.pone.0153631 (PMC4833323; doi:10.1371/journal.pone.0153631)
Supplement: S2 Table — Sequences in boldface were obtained for this study. Taxonomy is taken from GenBank. (PDF) [file pone.0153631.s014.pdf]

Accession Number	Latin name	Sex	Taxonomy Order	Superfamily	Family
NC_008452	*Acanthocardia tuberculata*		Veneroida	Cardioidea	Cardiidae
NC_021375	*Coelomactra antiquata*		Veneroida	Mactroidea	Mactridae
NC_008451	*Hiatella arctica*		Myoida	Hiatelloidea	Hiatellidae
**KP244451**	***Meretrix lamarckii***	**F**	**Veneroida**	**Veneroidea**	**Veneridae**
NC_016174	*Meretrix lamarckii*	F	Veneroida	Veneroidea	Veneridae
**KP244452**	***Meretrix lamarckii***	**M**	**Veneroida**	**Veneroidea**	**Veneridae**
NC_014809	*Meretrix lusoria*		Veneroida	Veneroidea	Veneridae
NC_022924	*Meretrix lyrata*		Veneroida	Veneroidea	Veneridae
NC_013188	*Meretrix meretrix*		Veneroida	Veneroidea	Veneridae
NC_012767	*Meretrix petechialis*		Veneroida	Veneroidea	Veneridae
NC_016889	*Paphia amabilis*		Veneroida	Veneroidea	Veneridae
NC_014579	*Paphia euglypta*		Veneroida	Veneroidea	Veneridae
NC_016890	*Paphia textile*		Veneroida	Veneroidea	Veneridae
NC_016891	*Paphia undulata*		Veneroida	Veneroidea	Veneridae
KP089983	*Ruditapes decussatus*		Veneroida	Veneroidea	Veneridae
AB065374	*Ruditapes philippinarum*	M	Veneroida	Veneroidea	Veneridae
NC_003354	*Ruditapes philippinarum*	F	Veneroida	Veneroidea	Veneridae
