# Supplementary material for: The Complete Female- and Male-Transmitted Mitochondrial Genome of Meretrix lamarckii
Source: PLoS One. 2016 Apr 15;11(4):e0153631. doi: 10.1371/journal.pone.0153631 (PMC4833323; doi:10.1371/journal.pone.0153631)
Supplement: S3 Table — Meretrix lamarckii F (A) and M (B) Unassigned Regions (URs). (PDF) [file pone.0153631.s015.pdf]

A ♀

UR	start	stop	Lenght
UR1	1,825	1,856	32
UR2	2,822	2,898	77
UR3	3,955	3,996	42
UR4	4,312	4,459	148
UR5	4,524	4,574	51
UR6	4,637	4,704	68
UR7	6,091	6,161	71
UR8	9,118	9,145	28
UR9	10,481	10,489	9
UR10	11,532	11,562	31
UR11	11,998	12,062	65
UR12	13,788	13,863	76
UR13	14,386	14,419	34
UR14	14,488	14,489	2
UR15	14,560	14,629	70
UR16	14,697	14,786	90
UR17	14,857	14,895	39
UR18	14,970	14,976	7
UR19	15,041	15,099	59
LUR	15,164	17,018	1,855
UR21	17,087	17,211	125
UR22	17,278	17,334	57
UR23	17,401	17,419	19
UR24	18,748	18,766	19
UR25	18,835	18,872	38
UR26	19,849	19,860	12
UR27	19,934	20,025	92

B ♂

UR	start	stop	Lenght
UR1	1,852	1,897	46
UR2	2,863	2,881	19
UR3	3,996	4,010	15
UR4	4,305	4,367	63
UR5	4,431	4,468	38
UR6	4,534	4,537	4
UR7	6,277	6,346	70
UR8	6,415	6,443	29
UR9	9,321	9,342	22
UR10	10,684	10,690	7
UR11	11,733	11,763	31
UR12	12,199	12,244	46
UR13	13,982	14,053	72
UR14	14,585	14,615	31
UR15	14,683	14,684	2
UR16	14,755	14,818	64
UR17	14,884	14,969	86
UR18	15,039	15,068	30
UR19	15,141	15,146	6
UR20	15,211	15,241	31
LUR1	15,306	15,999	694
LUR2	16,067	16,756	690
UR23	16,826	16,860	35
UR24	16,927	16,971	45
UR25	17,038	17,057	20
UR26	18,379	18,399	21
UR27	18,470	18,506	37
UR28	19,498	19,508	11
UR29	19,580	19,688	109
