# Supplementary material for: The Complete Female- and Male-Transmitted Mitochondrial Genome of Meretrix lamarckii
Source: PLoS One. 2016 Apr 15;11(4):e0153631. doi: 10.1371/journal.pone.0153631 (PMC4833323; doi:10.1371/journal.pone.0153631)
Supplement: S4 Table — The canonical 13 PCGs (bold) and all other Open Reading Frames (ORFs) are reported along with their start base, stop base, frame, and Glimmer score. F_ORF141 and M_ORF138 are shown in bold as well. (PDF) [file pone.0153631.s016.pdf]

F

Name	Start	Stop	Frame	Score
cox1	**1**	**1,824**	**1**	**13.55**
orf00019	1,757	1,849	2	2.54
orf00021	1,876	1,938	1	1.04
nad1	**1,919**	**2,821**	**2**	**14.37**
orf00033	2,791	2,856	1	2.87
nad2	**2,902**	**3,954**	**1**	**14.73**
nad4L	**3,997**	**4,311**	**1**	**15.19**
orf00057	4,336	4,368	1	0.05
orf00058	4,368	4,409	3	6.57
orf00059	4,406	4,450	2	9.71
orf00061	4,447	4,518	1	9.28
orf00062	4,500	4,526	3	15.76
orf00065	4,523	4,558	2	20.43
orf00067	4,617	4,664	3	6.71
cox2	**4,750**	**6,090**	**1**	**11.73**
orf00081	6,056	6,124	2	16.65
orf00083	6,121	6,147	1	39.21
orf00084	6,153	6,176	3	21.90
cytb	**6,227**	**7,492**	**2**	**12.28**
orf00106	7,510	7,575	1	7.42
orf00108	7,601	7,714	2	7.61
orf00111	7,730	7,822	2	4.19
orf00112	7,907	7,758	−3	4.20
orf00113	7,861	7,914	1	7.39
orf00114	7,957	8,019	1	4.03
orf00116	7,983	7,921	−1	0.55
orf00117	8,024	8,059	2	18.86
orf00119	8,095	8,145	1	12.07
orf00121	8,202	8,134	−1	2.73
orf00122	8,225	8,269	2	7.90
orf00124	8,324	8,202	−3	0.86
orf00125	8,365	8,442	1	1.43
orf00126	8,343	8,287	−1	0.82
orf00127	8,329	8,312	−2	−6.80
orf00128	8,451	8,468	3	32.65
orf00130	8,564	8,596	2	17.51
orf00131	8,593	8,610	1	21.86
orf00132	8,614	8,700	1	4.18
orf00133	8,639	8,752	2	6.98

M

Name	Start	Stop	Frame	Score
orf00001	19,687	25	2	22.54
orf00002	21	53	3	19.10
orf00003	50	88	2	24.61
cox1	**46**	**1,851**	**1**	**13.78**
orf00028	1,820	1,936	2	11.92
orf00031	2,055	1,972	−1	1.55
orf00032	2,096	2,007	−3	0.05
nad1	**2,041**	**2,862**	**1**	**15.80**
orf00044	2,802	2,891	3	2.95
orf00046	2,897	2,923	2	10.33
nad2	**2,940**	**3,995**	**3**	**17.31**
nad4L	**4,011**	**4,304**	**3**	**15.17**
orf00071	4,316	4,333	2	20.86
orf00073	4,374	4,433	3	7.47
orf00075	4,364	4,450	2	0.80
orf00076	4,457	4,483	2	5.95
orf00077	4,492	4,542	1	9.29
orf00078	4,514	4,558	2	7.96
orf00079	4,558	4,602	1	9.23
orf00080	4,625	4,681	2	13.27
orf00081	4,663	4,574	−2	0.05
orf00082	4,611	4,745	3	10.72
cox2	**4,702**	**6,276**	**1**	**10.47**
orf00102	6,276	6,317	3	6.99
orf00105	6,326	6,367	2	4.32
orf00107	6,413	6,433	2	12.30
orf00108	6,442	6,501	1	7.08
orf00109	6,522	6,457	−1	2.21
orf00110	6,619	6,482	−2	3.55
orf00111	6,653	6,688	2	34.16
orf00112	6,676	6,629	−2	−2.70
orf00113	6,671	6,615	−3	2.02
orf00114	6,628	6,795	1	15.08
cytb	**6,759**	**7,712**	**3**	**14.06**
orf00134	7,709	7,789	2	1.31
orf00135	7,872	7,732	−1	1.64
orf00138	7,857	7,952	3	8.68
orf00139	7,928	7,836	−3	0.92
orf00140	7,978	8,025	1	15.12

orf00135	8,797	8,678	−2	4.05	*orf00143*	8,004	8,117	3	4.65
orf00136	8,867	8,896	2	6.76	*orf00145*	8,175	8,222	3	3.76
orf00137	8,907	8,978	3	0.92	*orf00148*	8,238	8,270	3	3.87
orf00138	8,927	8,877	−3	6.46	*orf00150*	8,297	8,341	2	21.21
atp8	**8,965**	**9,117**	**1**	**13.63**	*orf00152*	8,348	8,401	2	2.87
nad4	9,119	10,480	2	14.20	*orf00154*	8,448	8,465	3	16.83
orf00165	10,483	10,509	1	26.99	*orf00155*	8,402	8,500	2	4.60
orf00168	10,595	10,500	−3	0.24	*orf00156*	8,526	8,509	−1	−14.09
orf00169	10,521	10,649	3	5.31	*orf00158*	8,595	8,639	3	3.11
atp6	**10,680**	**11,531**	**3**	**11.16**	*orf00159*	8,567	8,484	−3	0.69
orf00179	11,524	11,556	1	8.80	*orf00160*	8,566	8,670	1	6.43
nad3	11,563	11,997	1	8.71	*orf00161*	8,674	8,697	1	24.39
orf00188	11,994	11,953	−1	−0.77	*orf00163*	8,691	8,711	3	7.33
orf00189	12,047	12,142	2	9.75	*orf00164*	8,717	8,743	2	3.43
orf00190	12,142	12,165	1	26.42	*orf00165*	8,766	8,798	3	22.88
nad5	**12,144**	**13,787**	**3**	**14.84**	*orf00167*	8,849	8,947	2	6.00
orf00210	13,763	13,816	2	8.93	*orf00168*	8,873	8,838	−3	12.81
nad6	**13,864**	**14,385**	**1**	**16.34**	*orf00169*	8,871	8,788	−1	7.00
orf00225	14,417	14,491	2	1.57	*orf00170*	8,955	9,044	3	3.13
orf00226	14,592	14,645	3	6.02	*orf00171*	8,974	9,084	1	5.98
orf00227	14,615	14,595	−3	−2.23	*orf00172*	9,158	9,181	2	2.76
orf00228	14,617	14,438	−2	2.17	*orf00173*	9,168	9,185	3	0.03
orf00229	14,643	14,587	−1	1.23	*orf00174*	9,007	8,933	−2	0.40
orf00230	14,651	14,746	2	8.72	***atp8***	**9,186**	**9,320**	**3**	**18.08**
orf00231	14,756	14,779	2	23.77	***nad4***	**9,322**	**10,683**	**1**	**12.89**
orf00233	14,873	14,911	2	11.77	*orf00198*	10,712	10,741	2	12.64
orf00236	14,920	15,069	1	4.70	*orf00201*	10,707	10,850	3	4.70
orf00238	15,062	15,100	2	7.07	*orf00203*	10,831	10,887	1	1.98
orf00241	15,169	15,189	1	5.02	***atp6***	**10,881**	**11,732**	**3**	**12.09**
orf00242	15,216	15,245	3	12.45	*orf00214*	11,725	11,757	1	14.93
orf00243	15,304	15,321	1	36.33	***nad3***	**11,764**	**12,198**	**1**	**12.49**
orf00244	15,285	15,338	3	8.83	*orf00223*	12,170	12,205	2	4.39
orf00245	15,328	15,351	1	12.67	*orf00224*	12,192	12,245	3	8.07
orf00246	15,405	15,494	3	11.47	*orf00225*	12,235	12,264	1	7.18
orf00248	15,560	15,474	−3	3.49	***nad5***	**12,245**	**13,981**	**2**	**16.39**
F_ORF141	**15,627**	**15,487**	**−1**	**2.34**	*orf00252*	13,957	13,992	1	7.37
orf00250	15,637	15,699	1	3.54	*orf00254*	13,998	14,033	3	18.82
orf00252	15,651	15,797	3	6.92	*orf00256*	14,037	14,054	3	18.53
orf00253	15,676	15,596	−2	1.12	***nad6***	**14,054**	**14,584**	**2**	**17.86**
orf00254	15,784	15,912	1	1.11	*orf00263*	14,562	14,723	3	0.54
orf00255	15,981	16,004	3	5.57	*orf00264*	14,772	14,828	3	10.24
orf00257	16,096	16,055	−2	3.33	*orf00265*	14,798	14,833	2	8.64

orf00258	16,149	16,232	3	2.00	*orf00268*	14,833	14,886	1	6.87
orf00259	16,163	16,252	2	6.89	*orf00269*	14,886	14,936	3	23.45
orf00260	16,217	16,086	−3	3.42	*orf00270*	14,936	14,953	2	31.95
orf00264	16,339	16,578	1	16.70	*orf00272*	14,956	15,009	1	9.63
orf00268	16,505	16,684	2	12.34	*orf00273*	14,967	15,041	3	4.43
orf00270	16,709	16,759	2	29.29	*orf00274*	15,055	15,084	1	37.28
orf00273	16,829	16,852	2	14.64	*orf00277*	15,144	15,251	3	6.91
orf00274	16,852	16,872	1	2.15	*orf00279*	15,300	15,317	3	25.71
orf00275	16,869	16,934	3	14.25	*orf00282*	15,305	15,442	2	12.51
orf00276	16,942	16,965	1	14.64	*orf00283*	15,430	15,456	1	21.61
orf00279	17,090	16,962	−3	1.83	*orf00286*	15,637	15,512	−2	1.48
orf00281	17,020	17,145	1	8.94	*orf00287*	15,618	15,659	3	16.56
orf00282	17,145	17,201	3	22.23	*orf00288*	15,623	15,706	2	4.01
orf00284	17,214	17,261	3	8.84	***M_ORF138***	**15,793**	**15,656**	**−2**	**3.36**
orf00285	17,236	17,301	1	4.92	*orf00291*	15,788	15,913	2	4.49
orf00286	17,440	17,457	1	28.96	*orf00293*	15,879	15,929	3	19.85
orf00287	17,506	17,535	1	28.11	*orf00300*	16,085	16,050	−3	8.68
orf00288	17,541	17,573	3	19.54	*orf00305*	16,199	16,089	−3	3.27
orf00289	17,571	17,509	−1	6.91	*orf00306*	16,141	16,263	1	6.88
orf00290	17,543	17,629	2	5.26	*orf00311*	16,304	16,435	2	9.28
orf00293	17,747	17,568	−3	1.55	*orf00313*	16,438	16,476	1	28.09
orf00294	17,710	17,781	1	17.75	*orf00317*	16,443	16,601	3	13.20
orf00295	17,791	17,865	1	1.65	*orf00318*	16,621	16,653	1	13.03
orf00296	17,840	17,869	2	10.81	*orf00319*	16,650	16,673	3	8.73
orf00298	17,836	17,735	−2	1.52	*orf00322*	16,701	16,745	3	13.74
orf00299	17,974	18,000	1	18.96	*orf00323*	16,742	16,759	2	49.69
orf00300	18,046	17,972	−2	2.99	*orf00325*	16,759	16,812	1	8.92
orf00301	17,976	18,050	3	5.29	*orf00326*	16,767	16,829	3	2.53
orf00302	18,069	18,137	3	4.35	*orf00327*	16,834	16,875	1	16.32
orf00303	18,229	18,134	−2	3.75	*orf00330*	16,863	16,910	3	8.98
orf00304	18,159	18,248	3	17.26	*orf00331*	16,885	16,944	1	5.43
orf00307	18,263	18,298	2	9.33	*orf00334*	16,941	17,009	3	4.96
orf00308	18,335	18,352	2	11.56	*orf00337*	17,134	17,006	−2	1.52
orf00310	18,349	18,372	1	10.11	*orf00338*	17,133	17,162	3	11.87
orf00312	18,400	18,480	1	6.09	*orf00339*	17,156	17,185	2	1.31
orf00314	18,455	18,535	2	2.90	*orf00340*	17,189	17,230	2	1.41
orf00316	18,543	18,638	3	1.97	*orf00344*	17,287	17,337	1	8.56
orf00318	18,647	18,682	2	26.42	*orf00349*	17,523	17,585	3	7.54
orf00320	18,842	18,865	2	31.32	*orf00351*	17,569	17,423	−2	1.70
cox3	**18,868**	**19,848**	**1**	**14.46**	*orf00352*	17,592	17,621	3	6.08
orf00340	19,848	19,889	3	7.74	*orf00353*	17,481	17,380	−1	0.41
orf00341	19,932	19,967	3	9.89	*orf00354*	17,603	17,629	2	9.75

orf00343	19,974	20,024	3	20.17
orf00356	17,659	17,679	1	16.28
orf00357	17,717	17,767	2	5.08
orf00358	17,743	17,784	1	5.61
orf00359	17,815	17,726	−2	4.10
orf00360	17,814	17,858	3	1.04
orf00361	17,826	17,764	−1	1.04
orf00362	17,789	17,878	2	22.36
orf00363	17,879	17,908	2	9.65
orf00364	17,911	17,928	1	17.00
orf00366	17,895	17,999	3	3.69
orf00368	17,984	18,073	2	8.16
orf00369	18,078	18,110	3	2.67
orf00370	18,070	18,165	1	4.19
orf00372	18,195	18,230	3	26.78
orf00374	18,241	18,267	1	24.22
orf00376	18,271	18,312	1	16.20
orf00379	18,335	18,382	2	5.06
orf00380	18,442	18,483	1	0.93
orf00381	18,483	18,500	3	43.81
orf00382	18,500	18,517	2	15.29
cox3	**18,574**	**19,497**	**1**	**16.90**
orf00397	19,490	19,507	2	13.72
orf00399	19,497	19,523	3	13.57
orf00402	19,551	19,640	3	8.86
orf00403	19,619	19,687	2	8.32
orf00405	19,652	19,605	−3	−2.70
