# Supplementary material for: The Complete Female- and Male-Transmitted Mitochondrial Genome of Meretrix lamarckii
Source: PLoS One. 2016 Apr 15;11(4):e0153631. doi: 10.1371/journal.pone.0153631 (PMC4833323; doi:10.1371/journal.pone.0153631)
Supplement: S5 Table — Homologies of F_ORF141 with ORFs in other Meretrix mitochondrial genomes; the first entry is F_ORF141 itself. (PDF) [file pone.0153631.s017.pdf]

	Accession Number	Start	Stop	Length	Frame	Prob	e-value	p-value
M. lamarckii ♀	KP244451	15,627	15,490	138	-3	100.0	7.9E-42	5.3E-46
M. lusoria	NC_014809	15,051	14,992	60	-3	97.9	1.7E-06	1.2E-10
M. petechialis	NC_012767	14,969	14,910	60	-2	96.6	0.00042	2.8E-08
M. lamarckii ♂	KP244452	15,793	15,659	135	-1	95.9	0.00079	5.4E-08
M. lyrata	NC_022924	15,053	15,009	45	-2	92.9	0.014	9.2E-07
