# Supplementary material for: The Complete Female- and Male-Transmitted Mitochondrial Genome of Meretrix lamarckii
Source: PLoS One. 2016 Apr 15;11(4):e0153631. doi: 10.1371/journal.pone.0153631 (PMC4833323; doi:10.1371/journal.pone.0153631)
Supplement: S6 Table — (PDF) [file pone.0153631.s018.pdf]

tRNA	Jin-Nei distance
trnA	36.15
trnR	14.99
trnN	26.37
trnD	28.22
trnC	36.31
trnQ	24.04
trnE	26.32
trnG	67.68
trnH	19.72
trnI	10.66
trnL(NAA)	39.69
trnL(NAG)	39.65
trnK	19.72
trnM	28.66
trnF	33.49
trnP	12.70
trnS(NCT)	4.84
trnS(NGA)	10.15
trnT	58.64
trnW	20.11
trnY	20.03
trnV	26.76
