# Supplementary material for: The Complete Female- and Male-Transmitted Mitochondrial Genome of Meretrix lamarckii
Source: PLoS One. 2016 Apr 15;11(4):e0153631. doi: 10.1371/journal.pone.0153631 (PMC4833323; doi:10.1371/journal.pone.0153631)
Supplement: S7 Table — The Kimura aminoacid distance is listed for each PCG. F, female genome; M, male genome; NC_016174, published Meretrix lamarckii mitochondrial genome. The F-atp8 gene has 11 aminoacids at the 5' end that are lacking in the published atp8 gene; as the remaining part of the peptide sequence is identical, the pairwise deletion led to a Kimura distance of 0. (PDF) [file pone.0153631.s019.pdf]

	F vs NC_016174	M vs NC_016174
atp6	4.76	14.03
atp8	0.00	8.13
cox1	2.80	8.13
cox2	19.28	39.86
cox3	5.50	17.91
cytb	2.70	18.17
nad1	0.67	4.12
nad2	6.25	14.44
nad3	8.85	18.90
nad4	1.36	17.94
nad4L	4.20	11.12
nad5	8.49	20.63
nad6	2.35	32.16
