# Supplementary material for: The Complete Female- and Male-Transmitted Mitochondrial Genome of Meretrix lamarckii
Source: PLoS One. 2016 Apr 15;11(4):e0153631. doi: 10.1371/journal.pone.0153631 (PMC4833323; doi:10.1371/journal.pone.0153631)

♀

A

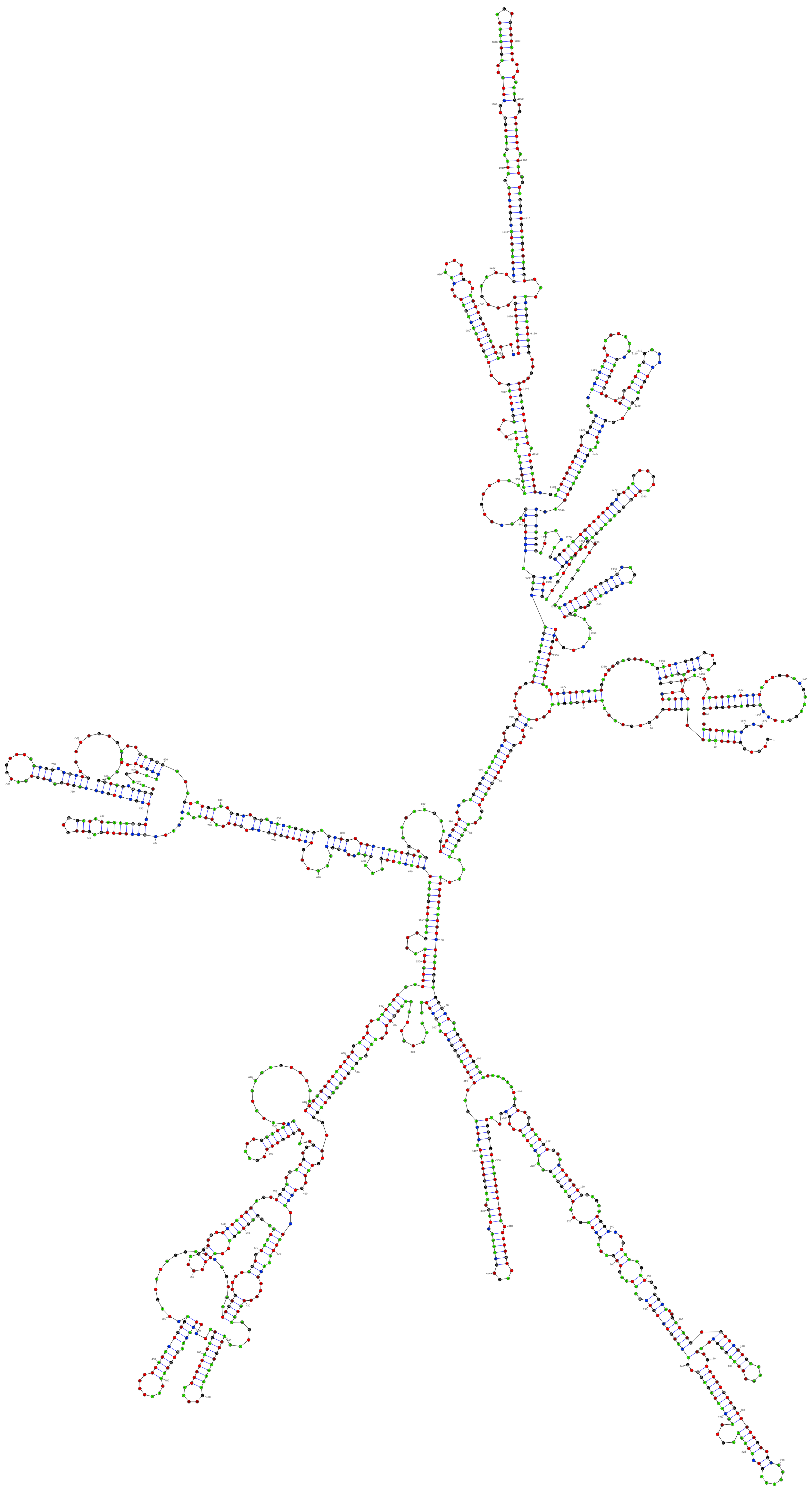

B

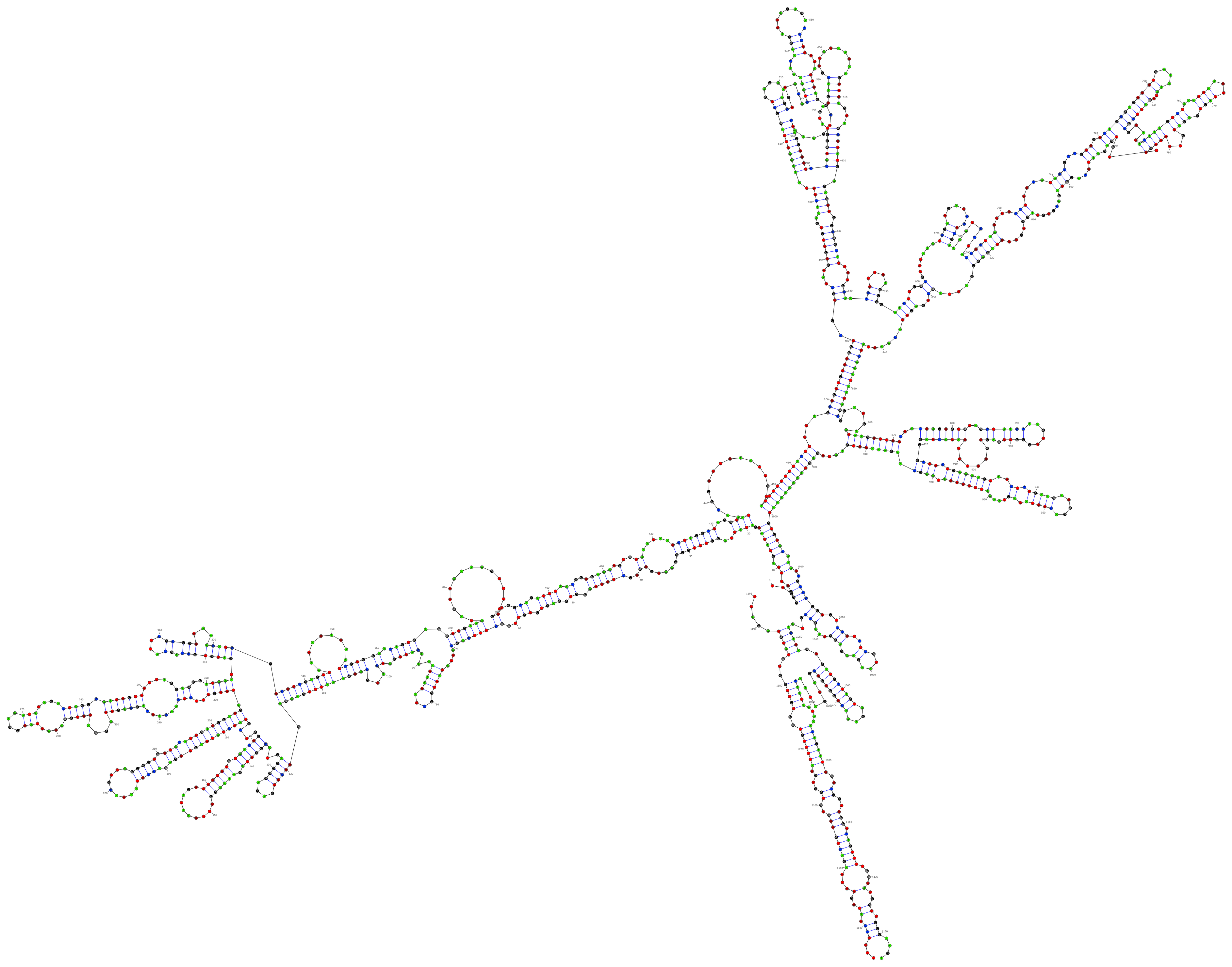

♂

C

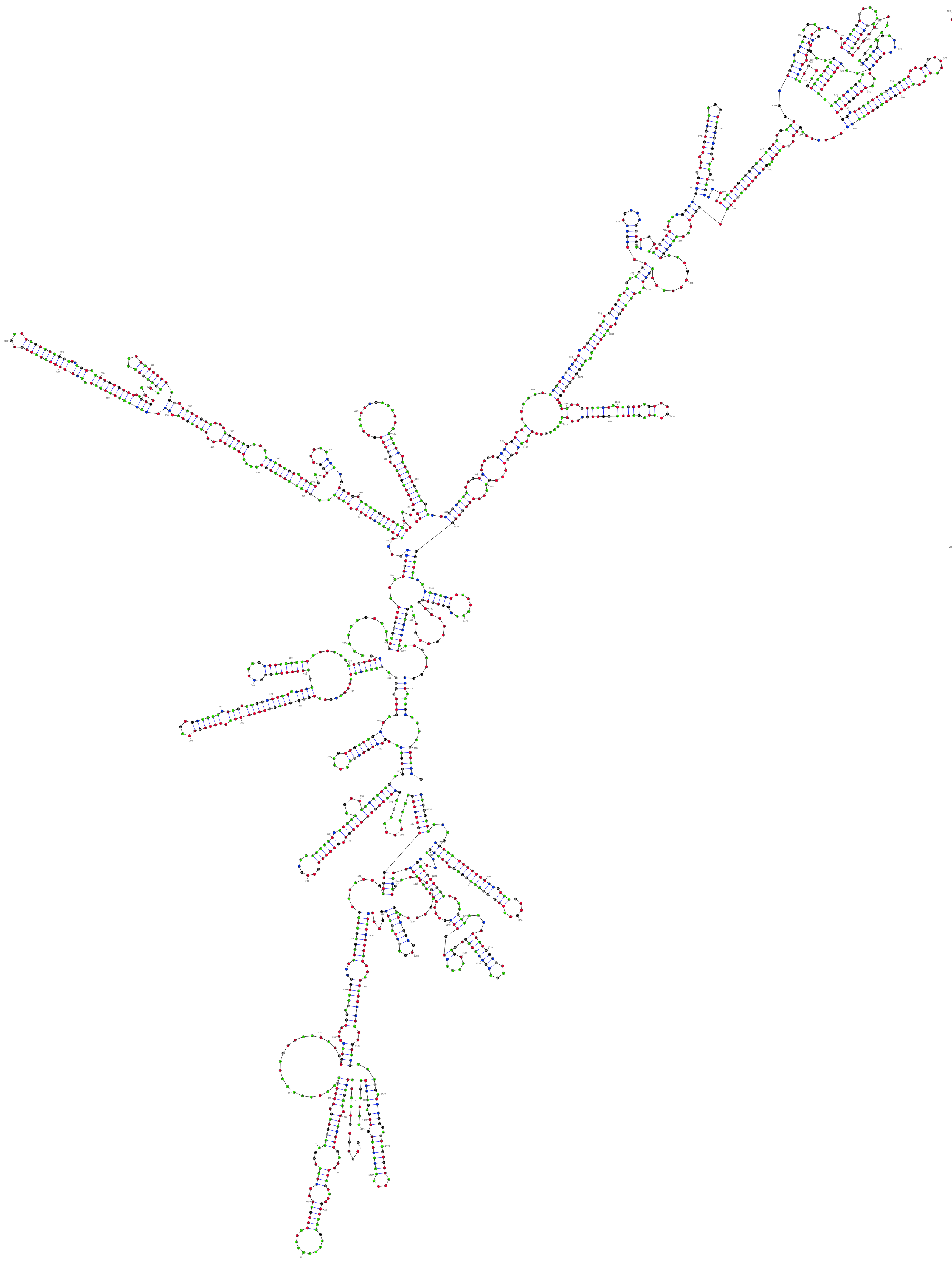

D

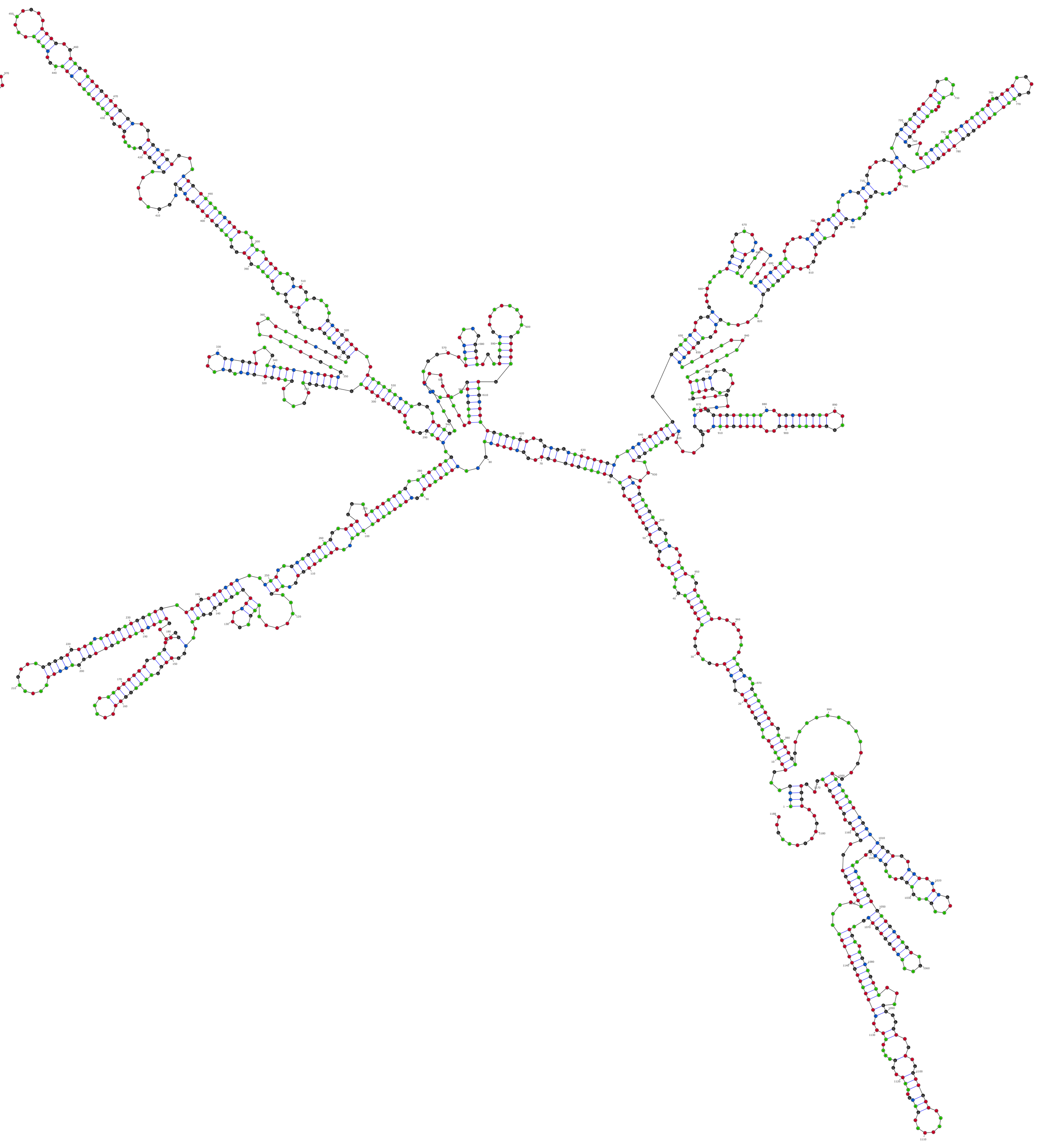

Supplement: S2 Fig — A, F-rrnL; B, F-rrnS; C, M-rrnL; D, M-rrnS. (PDF) [file pone.0153631.s002.pdf]
