# Supplementary material for: The Complete Female- and Male-Transmitted Mitochondrial Genome of Meretrix lamarckii
Source: PLoS One. 2016 Apr 15;11(4):e0153631. doi: 10.1371/journal.pone.0153631 (PMC4833323; doi:10.1371/journal.pone.0153631)
Supplement: S11 Fig — Phobius predictions of cox2 residue locations for all DUI species used for this work. The cox2 length is reported in the x axis; the y axis refers to the posterior probability of a given position to be part of a TMH (gray), cytoplasmic (green), non-cytoplasmic (blue), or part of a signal peptide (red). (PDF) [file pone.0153631.s011.pdf]

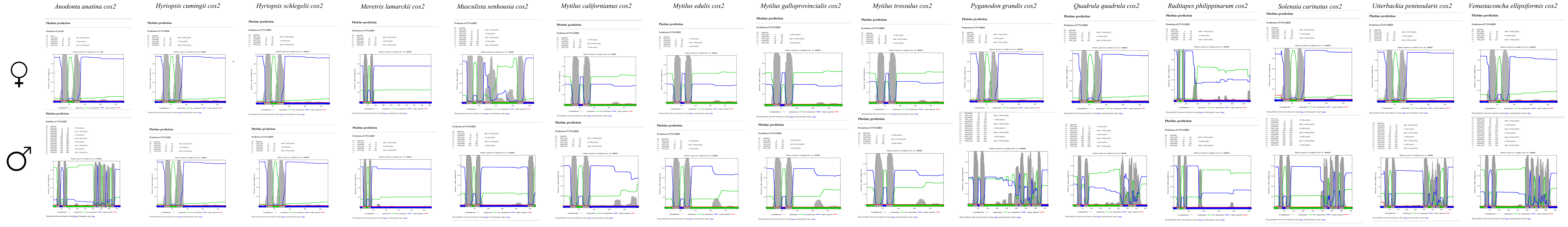
Anodonta anatina cox2
Hyriopsis cumingii cox2
Hyriopsis schlegelii cox2
Meretrix lamarckii cox2
Musculista senhousia cox2
Mytilus californianus cox2
Mytilus edulis cox2
Mytilus galloprovincialis cox2
Mytilus trossulus cox2
Pyganodon grandis cox2
Quadrula quadrula cox2
Ruditapes philippinarum cox2
Solenaia carinatus cox2
Utterbackia peninsularis cox2
Venustaconcha ellipsiformis cox2
♀
♂
